# Context-specific impact of antimicrobial stewardship on antibiotic use and antibiotic resistance in hospitals in a lower-middle-income country: results from an implementation study with a controlled interrupted time series design in Viet Nam

**DOI:** 10.1093/jacamr/dlag147

**Published:** 2026-07-22

**Authors:** Quynh Trang Le, Tien Viet Dung Vu, Minh Quang Le, Thi Thu Huyen Nguyen, Hai Vinh Vu, Minh Duc Chau, Thi Hoang Dung Em Vo, Thi Cam Tu Nguyen, Anh Quan Truong, Hong Khanh Nguyen, Nguyen Minh Hoa Le, Thomas Kesteman, Elizabeth Dodds Ashley, Deverick J Anderson, Hugo C Turner, Ngoc Thach Pham, Ben S Cooper, Marc Choisy, H Rogier van Doorn, Huong Thi Lan Vu

**Affiliations:** Oxford University Clinical Research Unit - Ha Noi Group, Bau Hamlet, Thien Loc, Ha Noi, Viet Nam; Oxford University Clinical Research Unit - Ha Noi Group, Bau Hamlet, Thien Loc, Ha Noi, Viet Nam; Viet Tiep Hospital, 1 Nha Thuong, Le Chan, Hai Phong, Viet Nam; Viet Tiep Hospital, 1 Nha Thuong, Le Chan, Hai Phong, Viet Nam; Viet Tiep Hospital, 1 Nha Thuong, Le Chan, Hai Phong, Viet Nam; Dong Thap Hospital, 39 Nguyen Van Tre, My Ngai, Dong Thap, Viet Nam; Dong Thap Hospital, 39 Nguyen Van Tre, My Ngai, Dong Thap, Viet Nam; Oxford University Clinical Research Unit - Ha Noi Group, Bau Hamlet, Thien Loc, Ha Noi, Viet Nam; Oxford University Clinical Research Unit - Ha Noi Group, Bau Hamlet, Thien Loc, Ha Noi, Viet Nam; Oxford University Clinical Research Unit - Ha Noi Group, Bau Hamlet, Thien Loc, Ha Noi, Viet Nam; National Hospital for Tropical Diseases, Bau Hamlet, Thien Loc, Ha Noi, Viet Nam; Oxford University Clinical Research Unit - Ha Noi Group, Bau Hamlet, Thien Loc, Ha Noi, Viet Nam; Duke Antimicrobial Stewardship Outreach Network, Duke Center for Antimicrobial Stewardship and Infection Prevention, Duke University, Durham, NC 27710, USA; Duke Antimicrobial Stewardship Outreach Network, Duke Center for Antimicrobial Stewardship and Infection Prevention, Duke University, Durham, NC 27710, USA; MRC Centre for Global Infectious Disease Analysis, School of Public Health, Imperial College London, London, UK; National Hospital for Tropical Diseases, Bau Hamlet, Thien Loc, Ha Noi, Viet Nam; NDM Centre for Global Health Research, Nuffield Department of Medicine, University of Oxford, Oxford, UK; NDM Centre for Global Health Research, Nuffield Department of Medicine, University of Oxford, Oxford, UK; Oxford University Clinical Research Unit - Modelling Group, Ho Chi Minh City, Viet Nam; Oxford University Clinical Research Unit - Ha Noi Group, Bau Hamlet, Thien Loc, Ha Noi, Viet Nam; NDM Centre for Global Health Research, Nuffield Department of Medicine, University of Oxford, Oxford, UK; Oxford University Clinical Research Unit - Ha Noi Group, Bau Hamlet, Thien Loc, Ha Noi, Viet Nam

## Abstract

**Objectives:**

We aimed to determine the effects of a 12-month antimicrobial stewardship (AMS) programme in two provincial-level general hospitals in Viet Nam, a lower-middle-income country.

**Methods:**

We performed controlled interrupted time-series analyses to evaluate the intervention effects on antibiotic use in days of therapy (DOT) per 1000 patient-days, antibiotic non-susceptibility percentage and patient outcomes. In each hospital, four wards received the intervention, and four wards acted as controls. Pre-intervention periods began in January 2019 and continued to May 2020 (Hospital 1) and July 2020 (Hospital 2).

**Results:**

Regarding antibiotic use, the intervention was associated with an immediate absolute reduction of 95.9 DOT/1000 patient-days (95%CI 10.9–180.8) in Hospital 1, while Hospital 2 showed no overall change but declining slopes in selected antibiotics including aminoglycosides and penicillin/beta-lactamase inhibitors. *Escherichia coli* showed decreasing non-susceptibility to aminoglycosides and third-generation cephalosporins in both hospitals. Carbapenem non-susceptibility increased for both *E. coli* and *Klebsiella* spp. in Hospital 2 but decreased for *Klebsiella* spp. in Hospital 1. Non-susceptibility of *P. aeruginosa* increased to carbapenems (Hospital 1), aminoglycosides, ciprofloxacin and ceftazidime (Hospital 2). *Acinetobacter* spp. showed increased non-susceptibility to aminoglycosides and piperacillin–tazobactam in Hospital 1 but decreased to carbapenems, ciprofloxacin and piperacillin–tazobactam in Hospital 2. No evidence of changes in mortality or hospitalization costs were observed in both hospitals.

**Conclusions:**

The impact of AMS varied between the two hospitals, highlighting the context-specific nature of implementation. Sustained monitoring of antibiotic use and resistance is needed to support locally tailored interventions that respond to evolving resistance epidemiology.

## Introduction

Widespread use of antibiotics in humans, food production animals and spillover into the environment has accelerated the emergence and transmission of drug-resistant bacteria.^1^ Antimicrobial stewardship (AMS) interventions are designed to target inappropriate antibiotic use to reduce selective pressure on resistant bacteria.^2^ A recent meta-analysis of the global impact of AMS programmes reported that such interventions were associated with an estimated mean reduction in the proportion of patients receiving antibiotic prescriptions by 10% (95% CI: 4%–15%).^3^ Importantly, AMS programmes need to monitor the impact on mortality to ensure interventions do not harm patients as well as the impact on antibiotic use and resistance.^4,5^ Previous studies reported no significant changes in patient outcomes, including mortality risks as a result of AMS interventions.^4,6^

The two study designs considered appropriate for evaluating the impact of AMS interventions are randomized controlled trials and quasi-experimental studies (non-randomized controlled trials, controlled before-and-after designs and interrupted time series).^4^ Recent evidence of the effect of AMS interventions on resistance outcomes comes from studies with weak designs. For example, between 2012 and 2017, only 8 of 26 studies used interrupted time series, and none included a control group in their analysis.^5^ In recent studies, only one study in Canada used a control group and demonstrated the impact of a comprehensive AMS programme with a sustained reduction of hospital-acquired antibiotic-resistant organisms.^7^ In 2022, a cluster randomized trial was conducted in 16 district-level hospitals in Viet Nam, a lower-middle-income country in Asia, reporting a reduction of 6.3% in inappropriate antibiotic prescriptions after 4 months of implementation of a multi-modal AMS programme.^8^ However, a short implementation follow-up period and the use of two point-prevalence audits in evaluating the impact of intervention limited the study to observe meaningful changes in prescribing behaviour and control for temporal variation in the analysis.

In this study, we aimed to evaluate the impact of an AMS intervention with a 1-year follow-up time period, through a controlled interrupted time-series design, in two provincial general hospitals in Viet Nam. Viet Nam developed its first national action plan for antimicrobial resistance and initiated AMS implementation in a local hospital network in 2013.^9^ Since then, national guidelines for antibiotic treatment and AMS implementation in hospitals have been issued, and local hospitals initiated their AMS programmes following these guidelines.^10^ We previously described our implementation research to assess the feasibility of AMS interventions in these two provincial hospitals in collaboration with the Duke Antimicrobial Stewardship Outreach Network.^11^ The theoretical framework for AMS implementation in this research was based on the assumption that hospitals are complex adaptive systems and that AMS teams could leverage their unique characteristics and interconnections to develop a locally feasible and sustainable programme. Prospective audit and feedback (PAF) was chosen as the core AMS intervention implemented at these two hospitals based on evidence from a previous systematic review on effective behaviour change interventions for antibiotic prescribing in hospitals.^4^

## Methods

### Study setting and population

**Figure 1.**
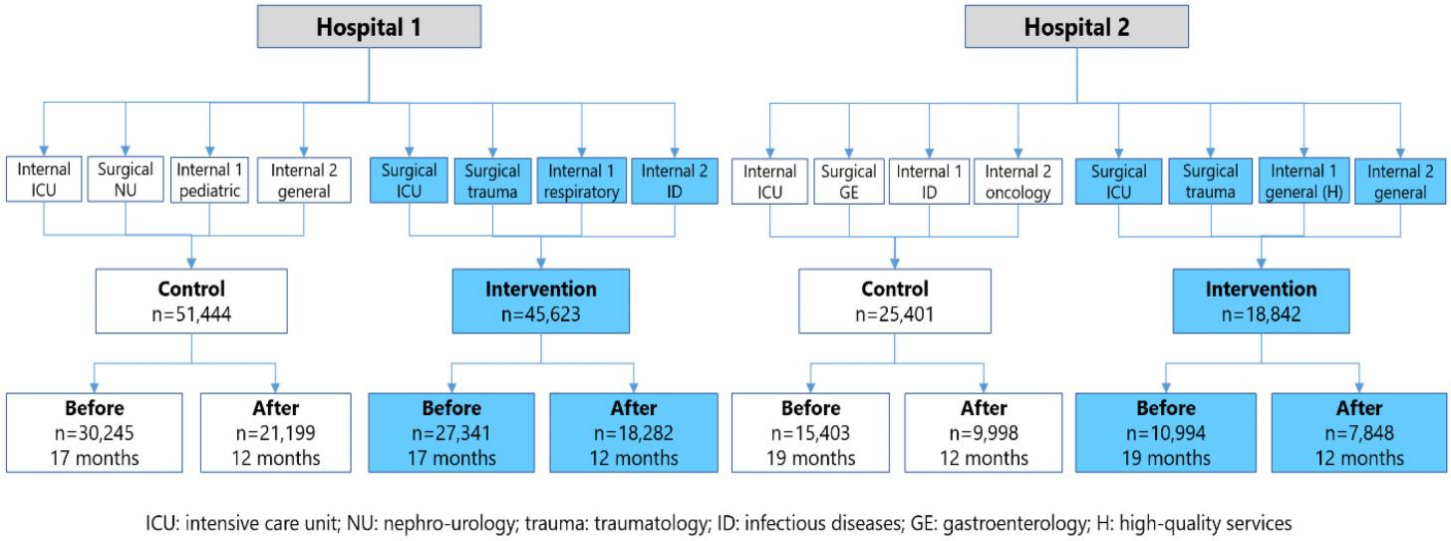
Patient flow before and after the AMS implementation started at Hospital 1 (Jan 2019–May 2021) and Hospital 2 (Jan 2019–Jul 2021). n: number of patients. Four ward pairs were included in each hospital: (1) ICU pair: surgical ICU versus internal ICU in both hospitals; (2) surgical pair: traumatology versus nephro-urology in Hospital 1 and traumatology versus gastroenterology in Hospital 2; (3) internal pair 1: respiratory versus paediatrics in Hospital 1 and general internal medicine (high-quality services) versus infectious diseases in Hospital 2 and (4) internal pair 2: infectious diseases versus general internal medicine in Hospital 1 and general internal medicine (normal services) versus oncology in Hospital 2. Control wards were included as contemporaneous comparison groups to account for secular trends and other background changes during the study period.

The study was implemented in two provincial hospitals in Viet Nam: Hospital 1 (1000 beds) and Hospital 2 (2000 beds). We selected 8/26 clinical wards in Hospital 1 and 8/27 clinical wards in Hospital 2, equally divided between intervention and control groups. Ward selection and assignment were described previously.^11^ Briefly, wards were selected based on two criteria: (1) higher-than-average antibiotic use in the hospital, defined in consultation with the hospital management team based on their local interpretation of pharmacy-reported data, and (2) willingness of the ward head to participate. All inpatients in the study wards during evaluation periods were included. Allocations of the wards in the two hospitals were similar in terms of clinical specialties, with four intervention versus control ward pairs (Figure 1). Detailed hospital characteristics are presented in Supplementary Data (available as Supplementary data at *JAC-AMR* Online) (Section A).

Before the intervention, each hospital had formally established an AMS committee and implemented the pre-authorization policy (which required doctors to obtain approval from the ward head and director board before using restricted antibiotics) following the 2016 national AMS guidelines.^12^ National guidelines for infection prevention and control (IPC) have been issued since 2009 (18/2009/TT-BYT), with documents and training provided by the Ministry of Health (MoH) to the hospitals since then.^13^ The 2016 AMS guidelines included the role of IPC staff in the AMS committee and described the set of responsibilities in implementing protocols for the isolation of patients with multidrug-resistant organisms, hand hygiene, use of personal protective equipment, sterilization of medical equipment, enhanced monitoring and outbreak investigations. Although there were cases of COVID-19 in 2020 in some specific areas of Viet Nam, the COVID-19 pandemic did not affect the two hospitals until late April 2021 in Hospital 1 and July 2021 in Hospital 2, towards the end of the intervention periods.^14^

### Interventions

Prior to the intervention, an AMS team was established to collect baseline data for assessments of needs, gaps, strengths and weaknesses to inform intervention planning.^11^ PAF was implemented by clinical pharmacists at the intervention wards, with support from the AMS doctor and other team members. The AMS doctor provided consultative input to the pharmacists for complicated cases and other situations requiring specialist advice. During the intervention period, clinical pharmacists made weekly visits to the intervention wards to review patients’ antibiotic prescriptions and provide recommendations for improvement where needed. Both hospitals continued the pre-authorization policy and routine IPC activities in all clinical wards as usual, including the study wards. Figure S1 outlines the timeline of project activities implemented before, during and after the intervention period, with PAF started on 1 June 2020 in Hospital 1 and 29 July 2020 in Hospital 2.

During the 1-year intervention period, the PAF activity at Hospital 1 was led by two clinical pharmacists who conducted a total of 1890 PAF reviews, while Hospital 2 assigned four clinical pharmacists conducting a total of 1628 PAF reviews.^11^ Pharmacists documented 82 actionable recommendations following PAF reviews at Hospital 1 (75% accepted by the treating doctors) and 128 at Hospital 2 (33% accepted). Common recommendations included de-escalation of antibiotics, microbiology and additional testing, medication switches and documentation of antibiotic indications in patient charts. Recommendations were communicated through face-to-face discussions, notes attached to patient charts or phone calls. AMS teams also prepared a monthly summary PAF report for each intervention ward in both hospitals, and clinical pharmacists presented this report in ward meetings and attended routine clinical ward rounds.

PAF was the primary intervention and training was provided as an implementation support component to facilitate its uptake (Figure S1). Training was delivered by experts from leading clinical and academic partners in Viet Nam and Duke University, focusing on antibiotic treatment for common infections, surgical prophylaxis, antibiotics and diagnostic stewardship. In addition, doctors in the intervention wards also participated in evaluation activities at baseline and during the intervention period conducted by AMS teams to inform planning and monitoring, including retrospective review of antibiotic prescriptions^15^ and repeated surveys on knowledge, attitudes and practices (KAP) related to antibiotic use, AMR and AMS; KAP surveys were also completed by doctors in the control wards.

Although PAF was applied to selected prescriptions, outcomes were assessed at the ward level using all inpatient prescriptions to capture both direct and indirect effects of the intervention.

### Patient and public involvement

Patients and/or the public were not involved in the design, conduct or reporting, or dissemination plans of this research.

### Outcome measures

#### Primary outcomes

##### Antibiotic use

The primary outcome is the amount of antibiotic use in days of therapy (DOT) per 1000 patient-days on a weekly time interval. Raw patient-level antibiotic prescription data from hospital information systems (HIS) were extracted together with patient administrative, diagnosis, discharge outcomes and bed day information. We calculated the antibiotic use proportion and DOT for all antibiotics and by subgroups (Table S3), including by Anatomical Therapeutic Chemical (ATC) classification and by AWaRe (Access, Watch, Reserve and Other) groups (2021 version) defined by the World Health Organization (WHO).^16^

##### Antibiotic non-susceptibility among hospital-acquired isolates

Microbiology data were deduplicated, i.e. if a patient had several specimens collected within 30 days, then the duplicate results were excluded. Hospital-acquired isolates were defined as those from specimens sampled at least 48 hours after hospital admission (counted for the first positive sample of the same specimen type and bacterium only). We measured the proportions of antibiotic non-susceptibility in five common organisms: *Escherichia coli, Klebsiella* spp., *Acinetobacter* spp*., Pseudomonas aeruginosa* and *Staphylococcus aureus*. Raw susceptibility data were extracted from the WHONET database of each hospital and interpreted using the AMR R package^17^ (interpretation using CLSI guidelines 2023). We assessed the following pathogen-drug combinations, which were considered relevant in the local epidemiological context:

- *E.coli* and *Klebsiella* spp.: third-generation cephalosporin (ceftriaxone or ceftazidime), aminoglycoside (gentamicin and one of amikacin or tobramycin), fluoroquinolone (ciprofloxacin), carbapenem (one of ertapenem, imipenem, meropenem or doripenem);
- *P. aeruginosa* and *Acinetobacter* spp.: third-generation cephalosporin (ceftazidime), aminoglycoside (one of amikacin or tobramycin), fluoroquinolone (ciprofloxacin), carbapenem (one of imipenem, meropenem or doripenem), piperacillin–tazobactam, aztreonam, colistin;
- *S. aureus:* Methicillin-resistant (MRSA) (oxacillin or cefoxitin).

#### Secondary outcomes

##### In-hospital mortality

In-hospital mortality per 1000 admitted patients on a weekly time interval, including patients who died in hospital and those who were discharged to die at home.

##### Cost of hospitalization

Includes all medical direct costs incurred during hospital admission as recorded in patients’ medical records at hospital discharge. All costs were converted from Viet Nam Dong to US Dollar in 2021 values, with costs incurred in 2019 and 2020 adjusted to the equivalent values in 2021 using Gross Domestic Product deflation rates.^18^

For antibiotic use, mortality and hospitalization costs, before-intevention time series were available from 1 Jan 2019 to 1 Jun 2021 in Hospital 1 and from 1 Jan 2019 to 29 Jul 2021 in Hospital 2. Antibiotic non-susceptibility data were available from 1 Jan 2014 to 31 Dec 2021 in Hospital 1, except for *S. aureus* from 1 Jan 2017 to 31 Dec 2021 and from 1 Dec 2017 to 31 Dec 2021 in Hospital 2. Further details of the outcomes can be found in Supplementary Data (Section B).

### Statistical methods

All analyses were conducted separately for each hospital. The proportion of patients with any antibiotic use during their hospital stay, DOT per 1000 patient-days, DOT percentage by AWaRe classification, in-hospital mortality per 1000 admitted patients and cost of hospitalization were summarized by pre- and post-intervention periods for all study wards and for each ward pair. Outcome variables were visualized and decomposed to examine potential patterns, trends and seasonality. Based on the available observations and periodic patterns, the aggregate unit, i.e. week or month, for each outcome was determined.

We used segmented regression within interrupted time-series (ITS) and controlled interrupted time-series (CITS) frameworks to estimate changes in level and trend in DOT per 1000 patient-days, antibiotic non-susceptibility, mortality and hospitalization costs after the AMS intervention. This approach was chosen to account for pre-existing trends, autocorrelation and potential seasonal patterns in the repeated weekly or monthly outcome data. The control wards provide a comparison group observed at the same time, allowing changes in the intervention wards to be distinguished from secular trends and other background influences affecting study outcomes. For the CITS analyses, the adjusted intervention effect was interpreted as the difference between the observed intervention trend and the counterfactual trend estimated from the control group. Detailed information on data analysis is presented in Supplementary Data (Section B). All analyses were conducted in R (v.4.3.1; R Core Team 2022).

This study report follows the criteria described for nonrandomized evaluations of behavioral and public health interventions^19^ (TREND Statement Checklist Table S4) and the Microbiology Investigation Criteria for Reporting Objectively (MICRO) framework^20^ (Table S5).

## Results

In total, there were 45 623 patients in the intervention group and 51 444 patients in the control group in Hospital 1 and 18 842 and 25 401 patients, respectively, in Hospital 2 (Figure 1). A summary of patient characteristics and outcomes is available in Supplementary Data (Section C), and the key contextual and implementation factors that may have contributed to the observed differences in the effects between the two hospitals are summarized in Table S2. Based on the frequency of observations for each outcome, in the ITS/CITS analysis, we applied a weekly interval for antibiotic use, mortality and hospitalization costs, and a monthly interval for antibiotic non-susceptibility (as the number of antibiotic non-susceptible isolates is relatively low).

### Antibiotic use: descriptive patterns

The proportion of patients receiving any antibiotic is presented descriptively in Table 1. In Hospital 1, it decreased slightly in the post-intervention period in both the intervention (from 71.4% to 70.1%) and control group (from 67.0% to 66.4%) in Hospital 1. In Hospital 2, antibiotic use decreased in the intervention (from 68.3% to 63.9%) but increased in the control group (from 72.0% to 76.3%).

**Table 1.** Study participants, antibiotic use, mortality, costs of hospitalization and antibiotic non-susceptibility among hospital-acquired isolates before and after the start of AMS intervention

Study variables	Hospital 1				Hospital 2			
	Intervention		Control		Intervention		Control	
	Before	After	Before	After	Before	After	Before	After
**Number of patients**								
All study wards	27 341	18 282	30 245	21 199	10 994	7848	15 403	9998
ICU pair	15 569	10 644	8359	6126	2256	1444	2799	2125
Surgical pair	4979	3462	2081	1574	3700	2730	4575	3190
Internal pair 1	3130	2954	13 146	8933	3472	2322	4289	2917
Internal pair 2	6602	3418	9074	6156	1806	1450	4220	2040
**Antibiotic use (%)**								
All study wards	71.4	70.1	67.0	66.4	68.3	63.9	72.0	76.3
ICU pair	71.1	63.9	52.0	49.5	93.7	91.6	88.6	94.3
Surgical pair	79.1	77.5	92.0	90.0	82.5	80.7	88.7	89.8
Internal pair 1	79.2	77.1	80.7	82.5	37.4	26.8	50.1	50.1
Internal pair 2	49.2	57.9	49.7	47.8	68.2	65.7	63.5	72.6
**DOT1000**								
All study wards	804	875	907	934	551	490	361	427
ICU pair	957	968	964	945	979	861	941	1178
Surgical pair	866	778	1361	1497	587	531	591	693
Internal pair 1	1100	1219	978	1016	215	190	117	122
Internal pair 2	491	571	635	604	561	527	472	581
**AWaRe group (% DOT) for all study wards**								
Access	11.3	19.1	14.4	19.7	21.9	14.0	21.0	15.6
Watch	88.1	80.0	84.6	78.9	60.1	58.7	58.6	57.8
Reserve	0.4	0.5	0.7	0.9	1.0	1.1	3.5	4.2
Other^a^	0.1	0.4	0.3	0.4	16.9	26.2	16.9	22.4
**Mortality** ^b^								
All study wards	6.7	9.6	45.2	58.8	43.8	41.7	92.3	81.5
ICU pair	5.0	8.2	160.2	199.8	194.1	213.3	368.3	382.1
Surgical pair	1.8	0.6	2.4	1.3	6.5	3.3	7.4	11.6
Internal pair 1	27.2	28.1	0.5	0.4	7.8	4.7	36.4	19.5
Internal pair 2	2.0	2.0	16.2	16.6	8.9	6.9	26.1	27.0
**Hospitalization cost** ^c^								
All study wards	183 (69–263)	192 (82–277)	97 (54–198)	106 (61–207)	491 (240–1027)	470 (249–922)	402 (193–835)	520 (265–979)
ICU pair	233 (186–324)	237 (186–323)	206 (112–382)	206 (111–382)	1575 (829–2571)	1453 (771–2341)	1575 (829–2571)	1210 (599–2127)
Surgical pair	146 (81–310)	142 (78–279)	176 (82–387)	178 (82–410)	521 (275–1003)	507 (266–908)	434 (237–760)	560 (326–909)
Internal pair 1	145 (82–250)	150 (80–264)	67 (41–106)	79 (48–121)	291 (165–494)	284 (166–454)	300 (187–529)	287 (180–521)
Internal pair 2	49 (35–71)	58 (42–87)	116 (65–234)	114 (66–222)	508 (256–880)	519 (282–812)	255 (123–504)	357 (198–633)
**Antibiotic non-susceptibility among hospital-acquired isolates** ^d^								
*E. coli*								
Aminoglycosides	242/657 (37)	65/145 (45)	395/806 (49)	104/176 (59)	12/48 (25)	3/14 (21)	27/74 (36)	7/26 (27)
Carbapenems	78/657 (12)	23/145 (16)	216/806 (27)	54/176 (31)	14/144 (10)	0/14 (0)	5/74 (7)	2/26 (8)
Ciprofloxacin	335/476 (70)	122/144 (85)	515/590 (87)	157/167 (94)	91/113 (81)	27/34 (79)	44/55 (80)	16/18 (89)
Third-geneneration cephalosporins	311/657 (47)	123/145 (85)	508/806 (63)	166/176 (94)	89/144 (62)	27/45 (60)	43/74 (58)	13/26 (50)
*Klebsiella spp.*								
Aminoglycosides	33/141 (23)	26/49 (53)	106/455 (23)	73/96 (76)	150/249 (60)	31/67 (46)	51/119 (43)	21/41 (51)
Carbapenems	32/141 (23)	23/49 (47)	95/455 (21)	68/96 (71)	130/249 (52)	21/67 (31)	50/119 (42)	19/41 (46)
Ciprofloxacin	46/105 (44)	34/45 (76)	154/268 57	81/96 (84)	166/205 (81)	35/56 (62)	69/91 (76)	23/30 (77)
Third-geneneration cephalosporins	60/141 (43)	43/49 (88)	164/455 (36)	86/96 (90)	174/249 (70)	36/67 (54)	70/119 (59)	23/41 (56)
*P. aeruginosa*								
Aminoglycosides	15/50 (30)	11/20 (55)	69/150 (46)	35/41 (85)	206/348 (59)	44/87 (51)	45/92 (49)	13/22 (59)
Carbapenems	21/50 (42)	5/20 (25)	74/150 (49)	28/41 (68)	174/348 (50)	42/87 (48)	49/92 (53)	12/22 (55)
Ciprofloxacin	22/42 (52)	13/20 (65)	63/109 (58)	28/40 (70)	193/293 (66)	46/75 (61)	47/83 (57)	12/19 (63)
Ceftazidime	12/43 (28)	8/20 (40)	53/108 (49)	25/40 (62)	91/316 (29)	14/61 (23)	44/86 (51)	4/15 (27)
Piperacillin– tazobactam	11/43 (26)	6/20 (30)	38/110 (35)	20/41 (49)	30/309 (10)	7/77 (9)	6/83 (7)	1/20 (5)
*Acinetobacter spp.*								
Aminoglycosides	120/140 (86)	50/60 (83)	172/310 (55)	65/70 (93)	244/310 (79)	88/103 (85)	153/198 (77)	47/60 (78)
Carbapenems	78/140 (56)	39/60 (65)	153/310 (49)	49/70 (70)	251/310 (81)	93/103 (90)	165/198 (83)	52/60 (87)
Ciprofloxacin	118/124 (95)	57/58 (98)	162/179 (91)	60/67 (90)	245/282 (87)	90/97 (93)	160/177 (90)	51/56 (91)
Ceftazidime	112/122 (92)	53/58 (91)	153/183 (84)	63/69 (91)	267/289 (92)	84/87 (97)	160/179 (89)	50/54 (93)
Piperacillin– tazobactam	102/124 (82)	44/60 (73)	151/183 (83)	52/70 (74)	219/254 (86)	89/93 (96)	142/156 (91)	51/54 (94)
*S. aureus*								
MRSA	74/85 (87)	50/57 (88)	210/278 (76)	88/112 (79)	71/93 (76)	19/25 (76)	60/79 (76)	11/12 (92)

MRSA, methicillin-resistant *S. aureus*.

^a^Antimicrobials classified under this group following WHO AWARE’s 2021 version include cefoperazone and beta-lactamase inhibitor, ticarcillin and beta-lactamase inhibitor.

^b^In-hospital mortality was calculated as those with outcome recorded as ‘death’ and ‘going home to die’ out of the patients admitted to the corresponding study wards (expressed as per 1000 admissions).

^c^Costs are presented in USD median (interquartile range) per day of hospitalization, calculated for individual patients, and with costs in 2019 and 2020 adjusted to the costs of 2021 using GDP deflation rates.

^d^Antibiotic non-susceptibility data are presented as the proportion of number of non-susceptible isolates over all isolates (percentage).

Use of Access antibiotics increased in both the intervention (from 11.3% to 19.1%) and the control group (from 14.4% to 19.7%) in Hospital 1. Conversely, Access antibiotic use decreased in Hospital 2, from 21.9% to 14.9% in the intervention and from 21.0% to 15.6% in the control group. Hospital 2 also showed an increase in the use of antibiotic categories classified as ‘Not recommended’ or unclassified in the AWaRe system, including cefoperazone/beta-lactamase inhibitor and ticarcillin/beta-lactamase inhibitor, from 16.9% to 26.2% in the intervention and 16.9% to 22.4% in the control group.

In the intervention groups, use of second-generation cephalosporins decreased from 13.8% to 5.2% and glycopeptides from 3.7% to 2.3% at Hospital 1, while penicillin/beta-lactamase inhibitors decreased from 27.9% to 26.4%, fourth-generation cephalosporins from 5.6% to 2.5% and fluoroquinolones from 22.6% to 16.5% at Hospital 2 (Table S6).

The overall number of days of antibiotic therapy per 1000 patient-days (DOT/1000 patient-days) increased in the post-intervention period in both the intervention and control groups in Hospital 1. In Hospital 2, DOT/1000 patient-days decreased in the intervention group in the post-intervention period, while the control group showed an increase (Table 1).

### Impact of intervention on antibiotic use: model results

**Figure 2.**
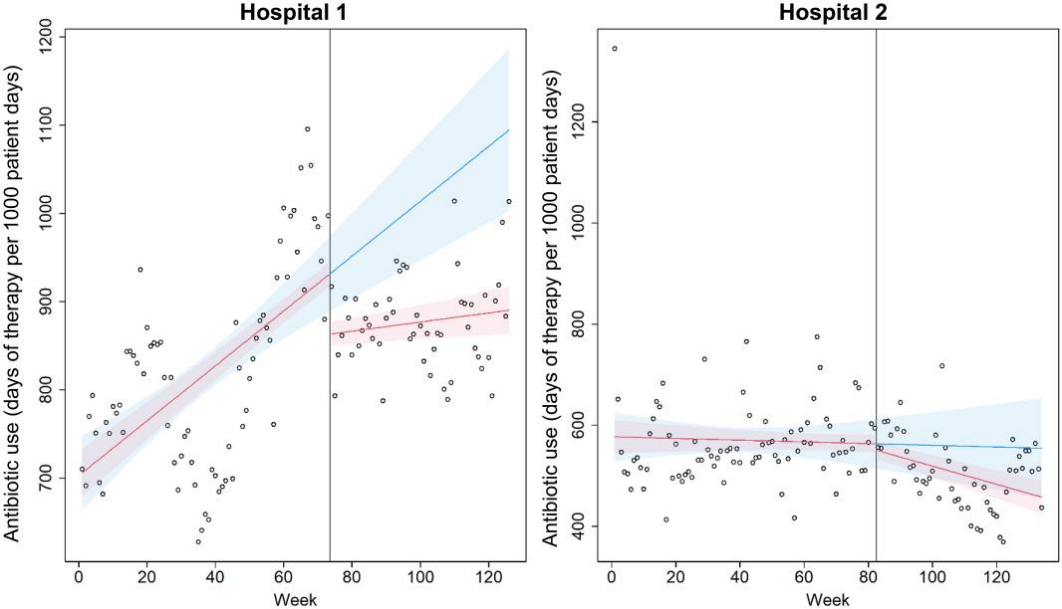
Antibiotic use before and after the start of AMS intervention for the intervention group in Hospital 1 and Hospital 2. Observed weekly DOT per 1000 patient-days are shown as points. Fitted trends (red lines) were estimated using segmented generalized linear regression. Counterfactual trends (blue lines) represent the projected pre-intervention trend in the absence of AMS implementation, estimated from the pre-intervention model and extrapolated into the post-intervention period in the ITS framework. Shaded areas represent 95% confidence intervals. Vertical lines indicate the start of AMS implementation, i.e. June 2020 for Hospital 1 and July 2020 for Hospital 2. Post-intervention observations begin from the first full week after AMS implementation. The *x*-axis shows time in months since January 2019 for both hospitals.

The ITS/CITS models showed changes in antibiotic use in Hospital 1 overall and for certain subgroups (Figure 2; Figure S6; Table S15). The intervention was associated with an immediate absolute reduction of 95.9 DOT/1000 patient-days in overall antibiotic use (95% CI 10.9–180.8). Reductions were also observed for glycopeptide antibacterials (level change 52.7 DOT/1000 patient-days, 95%CI 38.0–67.5, slope change 0.6, 95%CI 0.1–1.0), second-generation cephalosporins (slope change 1.3, 95%CI 0.5–2.0) and fluoroquinolones (slope change 2.7, 95%CI 0.4–5.0), while an increase was observed for beta-lactamase resistant penicillins (level change 7.3, 95%CI 0.2–14.3).

In Hospital 2, the CITS models did not show evidence of a change in the overall antibiotic use, but they showed decreases in aminoglycosides (slope change 0.6, 95%CI 0.4–0.8) and penicillin/beta-lactamase inhibitors (slope change 3.7, 95%CI 1.7–5.6); and an increase for beta-lactamase resistant penicillins (slope change 0.2, 95%CI 0.0–0.4). Detailed and further subgroup analysis results are provided in Table S15 and Figure S7.

### Impact of intervention on antibiotic non-susceptibility

The CITS models showed heterogeneous changes in non-susceptibility across organism-antibiotic combinations (Figure S6; Table S16). In *E. coli*, non-susceptibility decreased for aminoglycosides in Hospital 1 (slope change, odds ratio OR = 0.87, 95%CI 0.78–0.97) while in Hospital 2 it decreased for aminoglycosides (level change, OR = 0.81, 95%CI 0.35–1.00) and third-generation cephalosporins (level change, OR = 0.42, 95%CI 0.15–1.00) but increased for carbapenems (level change, OR = 2.21, 95%CI 1.00–8.31). In *Klebsiella* spp., non-susceptibility decreased for carbapenems (level change, OR = 0.77, 95%CI 0.33–1.00) and ciprofloxacin (level change, OR = 0.50, 95%CI 0.17–1.00) in Hospital 1, whereas increases were observed in Hospital 2 for carbapenems (level change OR = 1.06, 95%CI 1.00–1.95) and ciprofloxacin (level change OR = 1.36, 95%CI 1.00–2.50).

In *P. aeruginosa*, non-susceptibility increased for carbapenems (slope change OR = 1.11, 95%CI 1.00–1.22) in Hospital 1 and for aminoglycosides (slope change OR = 1.07, 95%CI 1.00–1.20), ciprofloxacin (slope change OR = 1.45, 95%CI 1.18–1.77) and ceftazidime (level change OR = 1.06, 95%CI 1.00–1.82; slope change OR = 1.03, 95%CI 1.00–1.19) in Hospital 2. In *Acinetobacter* spp., non-susceptibility increased for aminoglycosides (level change OR = 11.93, 95%CI 1.00–94.25; slope change OR = 1.07, 95%CI 1.00–1.27) and piperacillin/tazobactam (level change OR = 1.23, 95%CI 1.00–2.52) in Hospital 1 but decreased for carbapenems (slope change OR = 0.96, 95%CI 0.88–1.00), ciprofloxacin (slope change OR = 0.93, 95%CI 0.85–1.00) and piperacillin/tazobactam (slope change OR = 0.94, 95%CI 0.78–1.00) in Hospital 2.

The percentages of MRSA remained stable during the post-intervention period, with no evidence of change in the CITS models in both hospitals.

**Figure 3.**
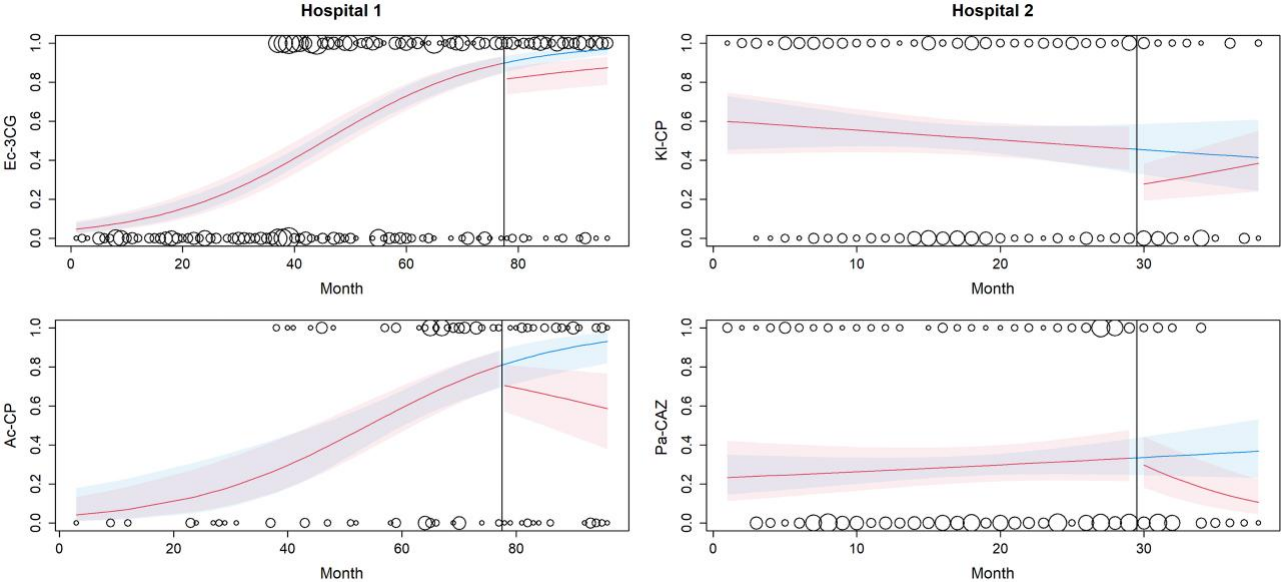
Hospital-acquired non-susceptibility among selected pathogen-drug combinations before and after the start of the AMS intervention in the intervention groups in Hospital 1 and Hospital 2. Observed monthly non-susceptibility outcomes are shown as points, with 1 indicating non-susceptibility and 0 indicating susceptibility; point size is proportional to the number of observations. Fitted trends (red lines) were estimated using segmented generalized linear regression with a binomial distribution and logit link. Counterfactual trends (blue lines) represent the projected pre-intervention trend in the absence of AMS implementation, estimated from the pre-intervention model and extrapolated into the post-intervention period in the ITS framework. Shaded areas represent 95% confidence intervals. Vertical lines indicate the start of AMS implementation, i.e. June 2020 for Hospital 1 and July 2020 for Hospital 2. Post-intervention observations begin from the first full month after AMS implementation. The *x*-axis shows time in months since January 2014 and January 2018 for Hospital 1 and Hospital 2, respectively. The four combinations were selected because of their clinical relevance, sufficient data and representation of common resistance patterns: *Escherichia coli*-third-generation cephalosporins (Ec-3GC) and Acinetobacter spp.-carbapenems (Ac-CP) in Hospital 1 and *Klebsiella* spp.-carbapenems (Kl-CP) and *Pseudomonas aeruginosa*-ceftazidime (Pa-CAZ) in Hospital 2.

Given the limited number of events for several pathogen-drug combinations, the corresponding estimates were imprecise. Figure 3 therefore highlights the most clinically relevant combinations for which sufficient data were available to support clearer presentation.

### In-hospital mortality and costs

**Figure 4.**
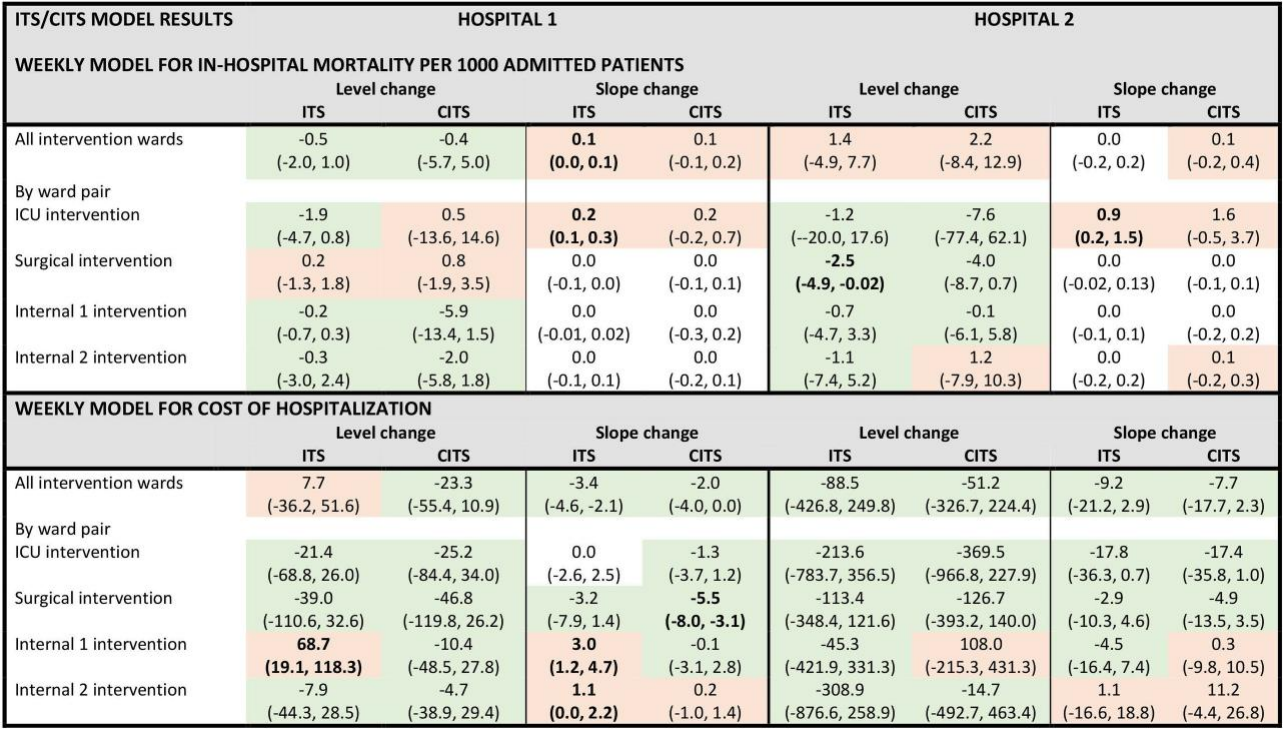
Results of ITS/CITS models for in-hospital mortality and cost of hospitalization in the intervention group at two hospitals. Bold values: statistically significant estimate; green shades: decreasing trends; red shades: increasing trends.

Baseline differences in mortality between intervention and control wards (Table 1) likely reflect underlying differences in case mix and severity rather than the effect of the intervention. The CITS models showed no evidence of a change in mortality in either hospital (Figure 4). Regarding hospitalization costs, the CITS model showed no evidence of an overall change across all intervention wards in either hospital; however, costs decreased in the intervention surgical ward in Hospital 1 (slope change 5.5 USD per week, 95%CI 3.1–8.0) (Figure 4).

Considering the costs for monthly AMS team activities (coordinator/pharmacists) and on-site training as routine components of the AMS programme at each hospital, the 1-year costs for AMS implementation were approximately 2809 USD for Hospital 1 and 2283 USD for Hospital 2 (Table S1).

## Discussion

We examined the impact of a PAF intervention on antibiotic use and resistance among common hospital-acquired pathogens. The effects differed between the two hospitals, underscoring the context-specific nature of AMS implementation. In Hospital 1, overall antibiotic consumption decreased in the intervention group with consistent effects on antibiotic non-susceptibility for *E. coli* and *Klebsiella* spp.; however, non-susceptibility increased for *Acinetobacter* spp. to aminoglycosides and piperacillin–tazobactam and *P. aeruginosa* to carbapenem, warranting further attention to local IPC practices and resistance dynamics. In Hospital 2, effects were more limited and mainly seen for selected antibiotic subgroups and some pathogen-drug combinations, with evidence of positive effects on *E.coli* (except for carbapenems) and *Acinetobacter* spp. but negative effects for *Klebsiella* spp. and *P. aeruginosa*. These heterogeneous findings likely reflect differences in baseline resistance, case mix, staffing, resource availability, staff engagement and doctor–pharmacist collaboration.

Literature supports the relationship between antibiotic use in hospitals and resistance prevalence among hospital-acquired isolates.^21^ Previous studies in Viet Nam, Malaysia, Uganda, India, China, Brazil, South Africa and Liberia have shown that AMS interventions can improve antibiotic use and, in some settings, influence resistance and cost outcomes, but the direction and magnitude of effect vary by local context and implementation model.^8,22–29^ For example, a systematic review of 13 studies from African countries, including South Africa, Kenya, Sudan, Tanzania and Egypt, found that most studies assessed antibiotic use and guideline compliance, while few reported clinical or financial outcomes and none assessed microbial outcomes.^29^ In Brazil, a broad-spectrum beta-lactam-sparing stewardship programme reduced broad-spectrum antibiotic use and expenditure while maintaining the susceptibility profile of Gram-negative bacteria.^26^ In a resource-limited community hospital in South Korea, an infectious disease-specialist-led AMS programme was associated with lower antibiotic consumption and reduced acquisition of multidrug-resistant organisms.^30^ Together, these studies suggest that AMS effects are context-dependent and that local implementation capacity strongly shapes outcomes.

A major strength of this study is the inclusion of a control group, which helped account for concurrent events, such as COVID-19 and IPC activities. In particular, AMS activities and staff attention to optimal antibiotic prescribing may have been negatively affected in the final weeks of the intervention in Hospital 1 by the early effects of Viet Nam’s fourth wave of COVID-19.^14^ We therefore interpret the estimated effects as the result of both the intervention and the broader implementation environment. The initial drop in antibiotic use in Hospital 1 may reflect heightened awareness at the start of pharmacist-led PAF and training, whereas the absence of a sustained slope change suggests incomplete uptake of PAF recommendations and the continued influence of contextual pressures. In Hospital 2, the increase in antibiotics classified as ‘Other’ under AWaRe may reflect broader system-level factors affecting antibiotic use outcomes, including centrally coordinated procurement, health insurance reimbursement requirements and antibiotic availability at the pharmacy department, alongside local prescribing practices.^11^

The study has several limitations. The pre- and post-intervention periods were relatively short, which restricted our ability to fully capture seasonal and historical trends. Antibiotic stock-outs, bidding cycles and insurance policies may also have influenced prescribing practices. In addition, spillover from intervention to control wards may have diluted the measured effect, and microbiology data quality, lack of molecular resistance data and potential misclassification of hospital-acquired infections limit inference about resistance dynamics. Finally, although ward pairs were matched within each hospital as closely as feasible, differences in case mix, baseline antibiotic use and ward-level clinical practices likely contributed to the observed variation in effects.

In conclusion, this study shows that AMS programmes, employing pharmacist-led prospective review of antibiotic prescriptions with feedback to doctors, can reduce antibiotic use and influence resistance patterns without evidence of harm to mortality or hospitalization costs, but the effects are not uniform across settings. In provincial hospitals in Viet Nam, the impact of AMS may depend on the alignment of local stewardship capacity, prescriber engagement, IPC practices, surveillance systems and other system-level factors such as procurement and availability, as differences in implementation conditions can shape both antibiotic use and resistance outcomes.

## Supplementary Material

dlag147_Supplementary_Data

## Data Availability

De-identified data may be obtained from the hospitals participating in this study when a data sharing agreement is in place.

## Supplementary data

Figure S1 and Tables S1 to S6 are available as Supplementary data at *JAC-AMR* Online.
