# Supplementary material for: Context-specific impact of antimicrobial stewardship on antibiotic use and antibiotic resistance in hospitals in a lower-middle-income country: results from an implementation study with a controlled interrupted time series design in Viet Nam
Source: JAC Antimicrob Resist. 2026 Jul 22;8(4):dlag147. doi: 10.1093/jacamr/dlag147 (PMC13390917; doi:10.1093/jacamr/dlag147)
Supplement: dlag147_Supplementary_Data [file dlag147_supplementary_data.docx]

**Supplementary Data**

**Manuscript title: Context-specific impact of antimicrobial stewardship on antibiotic use and antibiotic resistance in hospitals in a lower-middle income country - results from implementation study with a controlled interrupted time series design in Vietnam**

**Authors:** Le Quynh Trang^1^, Vu Tien Viet Dung^1^, Le Minh Quang^2^, Nguyen Thi Thu Huyen^2^, Vu Hai Vinh^2^, Chau Minh Duc^3^, Vo Thi Hoang Dung Em^3^, Nguyen Thi Cam Tu^1^, Truong Anh Quan^1^, Nguyen Hong Khanh^1^, Le Nguyen Minh Hoa^7^, Thomas Kesteman^1^, Elizabeth Dodds Ashley^5^, Deverick J. Anderson^5^, Hugo C Turner^6^, Pham Ngoc Thach^4^, Ben S Cooper^7^, Marc Choisy^1^, H Rogier van Doorn^1,7^, Vu Thi Lan Huong^1^

^1^Oxford University Clinical Research Unit, 78 Giai Phong, Hanoi, Viet Nam

^2^Viet Tiep Hospital, 1 Nha Thuong, Cat Dai, Le Chan, Hai Phong, Viet Nam

^3^Dong Thap Hospital, 144 Mai Van Khai, My Tan, Cao Lanh City, Đong Thap

^4^National Hospital for Tropical Diseases, 78 Giai Phong, Hanoi, Viet Nam

^5^Duke Antimicrobial Stewardship Outreach Network, Duke Center for Antimicrobial Stewardship and Infection Prevention, Duke University, Durham, NC 27710, United States

^6^ MRC Centre for Global Infectious Disease Analysis, School of Public Health, Imperial College London, London, UK.

^7^Centre for Tropical Medicine and Global Health, Nuffield Department of Medicine, University of Oxford, Oxford, UK

**Correspondence to** Vu Thi Lan Huong to [huongvtl@oucru.org](mailto:huongvtl@oucru.org)

Contents

[**A. Study setting and design** 4](#_Toc231728864)

[Table S1. Characteristics of two hospitals at the time of participating in the study 4](#_Toc231728865)

[Figure S1. The intervention period and timeline of study activities before and after start of intervention at each hospital 5](#_Toc231728866)

[Figure S2. COVID-19 Pandemic timeline in Vietnam in 2020-2021 6](#_Toc231728867)

[Table S2. Summary of contextual and implementation differences that may have contributed to heterogeneous AMS effects in Hospital 1 and Hospital 2 6](#_Toc231728868)

[**B. Study methods and reporting checklists** 7](#_Toc231728869)

[Outcome measures 7](#_Toc231728870)

[Table S3. List of antibiotic agents reported consumption during the evaluation periods at two hospitals 7](#_Toc231728871)

[Data analysis methods 8](#_Toc231728872)

[Table S4. TREND Statement Checklist 10](#_Toc231728873)

[Table S5. Assessment of criteria for clinical microbiology data reported in the study using the Microbiology Investigation Criteria for Reporting Objectively (MICRO) framework 14](#_Toc231728874)

[**C. Patient characteristics and summary outcome indicators** 20](#_Toc231728875)

[Figure S3. Number of patients by subgroups, mortality per 1000 admissions and number of microbiological isolates over time in each hospital in eight study wards at each hospital (vertical lines indicate the start of AMS intervention) 20](#_Toc231728876)

[Table S6. Summary of variables before and after the start of AMS intervention of the intervention and control group in two hospitals – more detailed results 24](#_Toc231728877)

[Table S7. Number of patients and proportions of patients with antibiotic use by main ICD10 diagnosis groups in intervention and control groups in two hospitals before and after the start of AMS implementation 28](#_Toc231728878)

[Table S8. Summary of bacterial isolates reported from the routine microbiological culture at two hospitals: all isolates and hospital-acquired (HA) isolates 29](#_Toc231728879)

[Figure S4. Summary of age and gender of patients with hospital acquired (HA) isolates, categorized by main pathogen in two hospitals 30](#_Toc231728880)

[Figure S5. Proportion of antibiotic non-susceptibility for main pathogen-drug combinations in intervention and control groups before and after the start of AMS intervention at two hospitals 31](#_Toc231728881)

[Table S9. Antibiotic non-susceptibility proportions (all isolates, blood/CSF isolates, ICU isolates) in study wards in Hospital 1 (Jan 2014 - Dec 2021) and Hospital 2 (Dec 2017 - Dec 2021) for common bacteria – *E. coli* 32](#_Toc231728882)

[Table S10. Antibiotic non-susceptibility proportions (all isolates, blood/CSF isolates, ICU isolates) in study wards in Hospital 1 (Jan 2014 - Dec 2021) and Hospital 2 (Dec 2017 - Dec 2021) for common bacteria – *Klebsiella* spp. 36](#_Toc231728883)

[Table S11. Antibiotic non-susceptibility proportions (all isolates, blood/CSF isolates, ICU isolates) in study wards in Hospital 1 (Jan 2014 - Dec 2021) and Hospital 2 (Dec 2017 - Dec 2021) for common bacteria – *P. aeruginosa* 40](#_Toc231728884)

[Table S12. Antibiotic non-susceptibility proportions (all isolates, blood/CSF isolates, ICU isolates) in study wards in Hospital 1 (Jan 2014 - Dec 2021) and Hospital 2 (Dec 2017 - Dec 2021) for common bacteria – *Acinetobacter* spp. 43](#_Toc231728885)

[Table S13. Antibiotic non-susceptibility proportions (all isolates, blood/CSF isolates, ICU isolates) in study wards in Hospital 1 (Jan 2014 - Dec 2021) and Hospital 2 (Dec 2017 - Dec 2021) for common bacteria *– S. aureus* 45](#_Toc231728886)

[Table S14. Antibiotic non-susceptibility for main pathogen-drug pairs in intervention and control groups before and after the start of AMS intervention at two hospitals: all isolates versus hospital acquired isolates 47](#_Toc231728887)

[**D. Model results** 51](#_Toc231728888)

[Figure S6. Results of ITS and CITS models for antibiotic use and antibiotic non-susceptibility among the hospital-acquired common pathogens identified from routine microbiology in the intervention group at two hospitals. 52](#_Toc231728889)

[Table S15. Results of ITS and controlled ITS models for antibiotic use in DOT per 1000 patient days 54](#_Toc231728890)

[Table S16. Results of ITS and controlled ITS models for monthly non-susceptibility proportions (for hospital-acquired isolates only) 56](#_Toc231728891)

[Table S17. Results of ITS and controlled ITS models for in-hospital mortality 59](#_Toc231728892)

[Table S18. Results of ITS and controlled ITS models for cost of hospitalization 60](#_Toc231728893)

[Table S19. Number of patient admissions to ICUs and ICU in-hospital mortality by main ICD10 diagnosis groups in intervention and control groups in two hospitals before and after the start of AMS implementation 61](#_Toc231728894)

[Figure S7. Weekly trends in antibiotic use (number of DOT per 1000 patient-days) in the intervention (blue line) and control group (black line) overall (left column) and stratified by study ward pair in each hospital (right column). 62](#_Toc231728895)

[**E. Model diagnostics** 63](#_Toc231728896)

[Table S20. Diagnostic plots for ITS/CITS ARIMA models for antibiotic use in DOT per 1000 patient days 63](#_Toc231728897)

[Table S21. Diagnostic plots for ITS/CITS ARIMA models for antibiotic non-susceptibility outcomes 77](#_Toc231728898)

[Table S22. Diagnostic plots for ITS/CITS ARIMA models for in-hospital mortality outcomes 92](#_Toc231728899)

[Table S23. Diagnostic plots for ITS/CITS ARIMA models for costs of hospitalization 96](#_Toc231728900)

[**References** 100](#_Toc231728901)

#

# **A. Study setting and design**

## Table S1. Characteristics of two hospitals at the time of participating in the study

| **Characteristic** | **Hospital 1** | **Hospital 2** |
| --- | --- | --- |
| Planned bed capacity | 1,000 | 2,000 |
| Annual outpatient visits | 710,000 visits per year | 400,000 visits per year |
| Annual inpatient admissions | 65,000 patients per year | 100,000 patients per year |
| Total number of clinical wards | 26 | 27 |
| Total number of doctors | 227 | 420 |
| Number of ID doctors | 9 | 12 |
| Number of pharmacists | 45 | 70 |
| Number of microbiologists | 10 | 20 |
| Number of staff in Infection Prevention and Control unit | 30 | 50 |
| AMS committee and program following the national AMS guideline in 2016 | Established as a sub-committee under the Drug and Therapy Committee in Oct 2018. Membership includes: team leader (Vice-director), 01 deputy team leader (Doctor from ICU-Poison Control), 01 secretary (Clinical Pharmacist) and 19 members who are the head of the clinical departments. Following Ministry of Health guidelines on antibiotic treatment (issued in 2015), IV-to-PO switch and pre-authorization of restricted antibiotics (drugs that required approval before prescribing for patients). | Established as a sub-committee under the Drug and Therapy Committee in Nov 2018. Membership includes: 2 vice-directors (as team leader and deputy team leader), 1 deputy-head of pharmacy (as secretary), 1 head of microbiology, 1 head of ICU, and 1 head of cardiology. Following Ministry of Health guidelines on antibiotic treatment (issued in 2015), IV-to-PO switch and pre-authorization of restricted antibiotics (drugs that required approval before prescribing for patients). |
| Electronic data systems | Hospital Information System (HIS), WHONET for antibiotic susceptibility results | Hospital information system (HIS),  Laboratory Information System (LIS), WHONET for antibiotic susceptibility results |
| Intervention group | surgical intensive care unit, traumatology, respiratory/musculoskelectal system (internal 1), and infectious diseases ward (internal 2) | surgical intensive care unit, traumatology and high-quality-general medicine (provide treatment services with better quality based on patient’s request) (internal 1), normal general internal medicine ward (internal 2) |
| Control group | internal intensive care unit, surgical nephro-urology, paediatrics (internal 1) and general internal medicine ward (internal 2) | internal intensive care unit, surgical gastroenterology, infectious diseases (internal 1), oncology ward (internal 2) |
| Time periods for antibiotic use, mortality, length of stay, hospitalization costs* | Before: 01 Jan 2019 – 30 May 2020  (17 months)  After: 01 Jun 2020 – 01 Jun 2021  (12 months) | Before: 01 Jan 2019 – 28 Jul 2020  (19 months)  After: 29 Jul 2020 – 29 Jul 2021  (12 months) |
| Time periods for antibiotic resistant proportions* | Before: Jan 2014-30 May 2020  (89 months)  After: 01 Jun 2020 -Dec 2021  (19 months) | Before: Dec 2017-28 Jul 2020  (32 months)  After: 29 Jul 2020 -Dec 2021  (17 months) |
| AMS intervention started in 2020 as part of the current project | Prospective audit with feedback to doctors (PAF) | Prospective audit with feedback to doctors (PAF) |
| Number of clinical pharmacists participating in AMS intervention | 2 | 4 |
| Number of patient records reviewed in prospective audit and feedback | 1,890 | 1,628 |
| Local program costs for the implementation of AMS program in one year | 2,809 USD for essential running and training costs (3 trainings), staff costs are not included as these would normally be part of the responsibilities of existing staff at the hospital | 2,283USD for essential running and training costs (2 trainings), staff costs are not included as these would normally be part of the responsibilities of existing staff at the hospital |
| Implementation cost components included | AMS team monthly activities, on-site training activities | AMS team monthly activities, on-site training |

*Before and after time periods were defined using the time point when prospective audit with feedback was started and maintained by clinical pharmacists on antibiotic prescriptions to doctors. For data on resistant proportions, longer data timeframes were available and therefore used for the analyses.

## Figure S1. The intervention period and timeline of study activities before and after start of intervention at each hospital^[[1]](#endnote-1)^


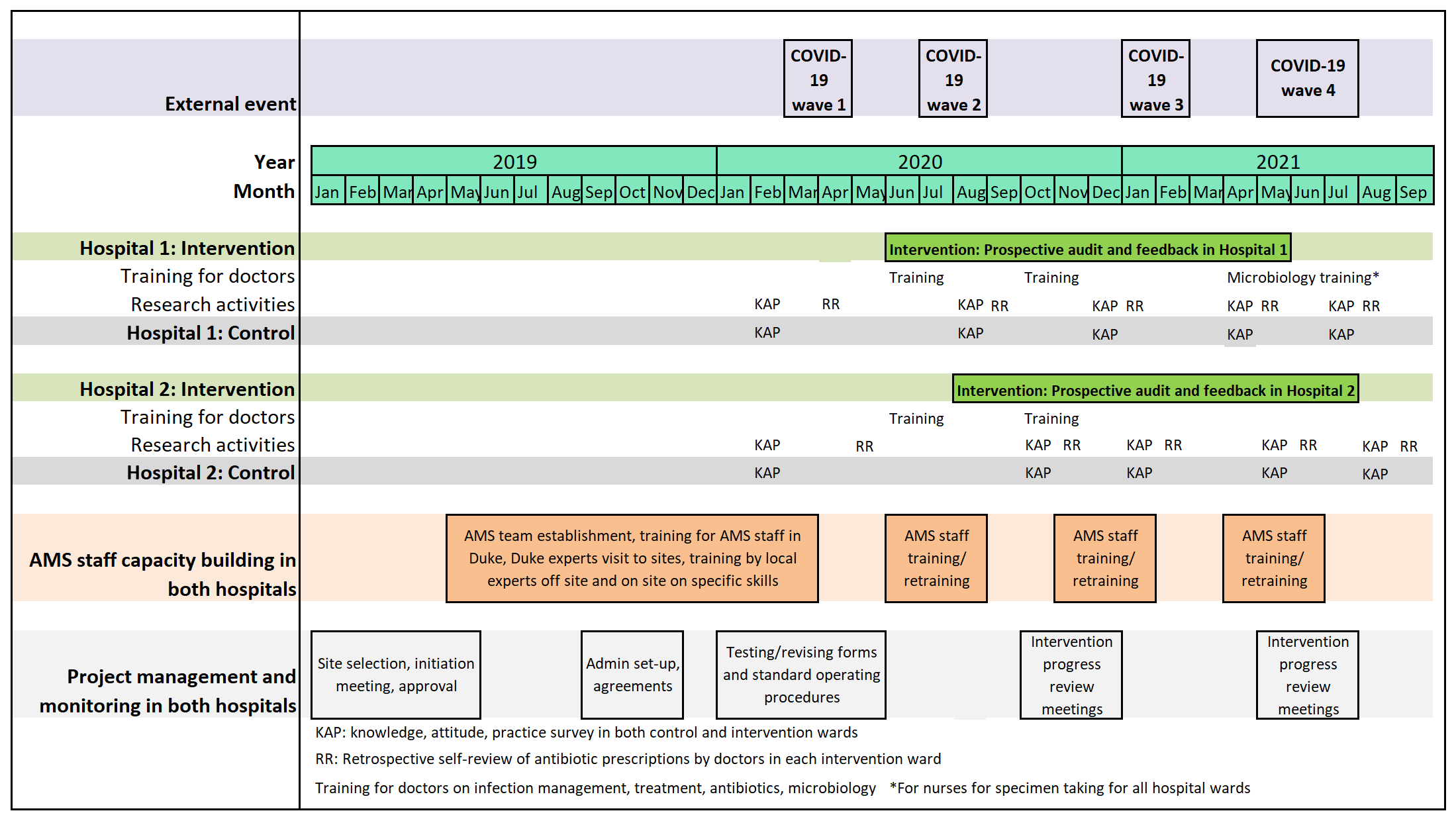


## Figure S2. COVID-19 Pandemic timeline in Vietnam in 2020-2021^[[2]](#endnote-2)^

## Table S2. Summary of contextual and implementation differences that may have contributed to heterogeneous AMS effects in Hospital 1 and Hospital 2

| **Contextual / implementation factor** | **Hospital 1** | **Hospital 2** | **Why it may matter** |
| --- | --- | --- | --- |
| Planned bed capacity / scale | 1,000 beds | 2,000 beds | Different scale may affect workflow, supervision, and AMS uptake |
| Clinical workforce | 227 doctors; 45 pharmacists; 10 microbiologists; 30 IPC staff | 420 doctors; 70 pharmacists; 20 microbiologists; 50 IPC staff | Different staff mix may affect feasibility and intensity of AMS support |
| Wards selected for the study | 8/26 clinical wards | 8/27 clinical wards | Wards were selected for high antibiotic use and ward-head willingness, which may shape engagement |
| Main intervention staffing for PAF | 2 clinical pharmacists | 4 clinical pharmacists | Different staffing likely affected number of reviews and depth of follow-up |
| PAF recommendation acceptance | 75% accepted | 33% accepted | Strongly suggests different uptake of AMS advice |
| Patient case mix in intervention wards | More respiratory, infectious, and injury/poisoning diagnoses | More injury/poisoning, cardiovascular, and oncology diagnoses | Different diagnosis profiles can drive different antibiotic prescribing needs and resistance ecology, making the intervention effects less directly comparable across hospitals. |
| Baseline antibiotic use | Higher baseline use in some wards; overall use differed by pair | Different baseline use across ward pairs | A different starting point can change the apparent intervention effect |
| Baseline resistance profile | Different pathogen-drug patterns across wards | Different pathogen-drug patterns across wards | Local resistance ecology may change which outcomes can improve |
| Data systems | HIS + WHONET | HIS + LIS + WHONET | Different electronic data environments may affect data quality and laboratory workflows |
| Local implementation context across hospital and wards | Same national AMS framework, but different ward-level uptake | Same national AMS framework, but different ward-level uptake | Same core AMS intervention was delivered under different real-world conditions which likely affected how much the intervention was accepted, sustained, and translated into measurable outcomes |

# **B. Study methods and reporting checklists**

## Outcome measures

Primary outcomes:

*Antibiotic use:* The primary outcome is the amount of antibiotic use in Days of Therapy (DOT) per 1000 patient-days on a weekly time interval. Raw patient-level antibiotic prescription data from hospital information systems (HIS) were extracted together with patient administrative, diagnosis, discharge outcomes and bed day information. From these data, we calculated the antibiotic use indicators overall and by Anatomical Therapeutic Chemical (ATC) classification of chemical therapeutic subgroup defined by the World Health Organization (WHO). Patients could move between study wards; each patient was counted only once under each grouping (and antibiotics used by the patient on a specific day were counted for the ward where the patient stayed on that day). We also described antibiotic use before and after the start of the AMS intervention by calculating the proportions of patients admitted to each study ward who used at least one antibiotic, and proportions of all using antibiotics by AWaRe (Access, Watch, Reserve, and Other) groups (2021 version) defined by WHO.

## Table S3. List of antibiotic agents reported consumption during the evaluation periods at two hospitals

| **Antibiotic Subgroup** | **Specific antibiotic agents reported** |
| --- | --- |
| Carbapenem | Biapenem, Doripenem, Ertapenem, Imipenem/cilastin, Meropenem, Meropenem/vaborbactam, Panipenem, Tebipenem |
| Cephalosporin – fourth generation | Cefepime, Cefozopran, Cefpirome |
| Cephalosporin –third generation | Cefetamet-pivoxil, Cefixime, Cefmenoxime, Cefodizime, Cefoperazone, Cefoperazone/ beta-lactamase inhibitors, Cefotaxime, Cefpiramide, Cefpodoxime-proxetil, Cefsulodin, Ceftazidime, Ceftazidime/avibactam, Cefteram-Pivoxil, Ceftibuten, Ceftizoxime, Ceftriaxone, Latamoxef |
| Cephalosporin – second generation | Cefmetazole, Cefminox, Cefonicid, Ceforanide, Cefotetan, Cefotiam, Cefoxitin, Cefprozil, Cefuroxime, Flomoxef, Loracarbef |
| Fluoroquinolone | Ciprofloxacin, Delafloxacin, Enoxacin, Fleroxacin, Garenoxacin, Gatifloxacin, Gemifloxacin, Grepafloxacin, Levofloxacin, Lomefloxacin, Norfloxacin, Ofloxacin, Pazufloxacin, Pefloxacin, Prulifloxacin, Rufloxacin, Sitafloxacin, Sparfloxacin, Temafloxacin, Tosufloxacin, Trovafloxacin |
| Penicillin/beta-lactamase inhibitor | Amoxicillin/clavulanic-acid, Ampicillin/sulbactam, Piperacillin/tazobactam, Sultamicillin, Ticarcillin/clavulanic-acid |
| Beta-lactamase resistant penicillin | Cloxacillin, Dicloxacillin, Flucloxacillin, Meticillin, Nafcillin, Oxacillin |
| Aminoglycoside | Dibekacin, Gentamicin, Isepamicin, Kanamycin, Neomycin, Netilmicin, Plazomicin, Ribostamycin, Sisomicin, Tobramycin |
| Glycopeptide antibacterial | Dalbavancin, Oritavacin, Teicoplanin, Telavancin, Vancomycin |
| Polymyxin | Colistin, Polymyxin |
| Imidazole derivative | Metronidazole, Ornidazole, Tinidazole |
| Macrolide | Clarithromycin, Dirithromycin, Erythromycin, Flurithromycin, Josamycin, Midecamycin, Miocamycin, Oleandomycin, Rokitamycin, Roxithromycin, Solithromycin, Spiramycin, Telithromycin, Troleandomycin |

*Antibiotic non-susceptibility among hospital-acquired isolates:* Microbiology data were deduplicated, i.e. if a patient had several specimens collected within 30-days, then the duplicate results were excluded . Hospital-acquired isolates were defined as those from specimens sampled at least 48 hours after hospital admission (counted for the first positive sample of the same specimen type and bacterium only). Hospital-acquired isolates were identified and analysed for the change in the non-susceptibility proportion after intervention. We measured the proportions of antibiotic non-susceptibility in five common organisms identified in routine clinical specimens from all bodily sites, excluding specimens for screening purposes: *Escherichia coli, Klebsiella* spp*.*, *Acinetobacter* spp*., Pseudomonas aeruginosa,* and *Staphylococcus aureus*. Raw susceptibility data for were extracted from the WHONET database of each hospital and interpreted using the AMR R package (interpretation using CLSI guidelines 2023). Non-susceptibility proportions were calculated as the ratio of the number of non-susceptible isolates to the number of tested isolates for a specific organism. We reported microbiology data following the MICRO framework recommendations for the following pathogen-drug combinations which were considered relevant in the local epidemiological context:

- *E.coli* and *Klebsiella* spp.: third-generation cephalosporin (ceftriaxone or ceftazidime), aminoglycoside (gentamicin and one of amikacin or tobramycin), fluoroquinolone (ciprofloxacin), carbapenem (one of ertapenem, imipenem, meropenem, or doripenem);
- *P. aeruginosa* and *Acinetobacter* spp.: third-generation cephalosporin (ceftazidime), aminoglycoside (one of amikacin or tobramycin), fluoroquinolone (ciprofloxacin), carbapenem (one of imipenem, meropenem, or doripenem), piperacillin-tazobactam, aztreonam, colistin;
- *S. aureus:* Methicillin-resistant (MRSA) (oxacillin or cefoxitin).

Secondary outcomes:

In-hospital mortality: In-hospital mortality per 1000 admitted patients on a weekly time interval, including patients who died in hospital and those who were discharged to die at home.

Cost of hospitalisation: Includes all costs incurred during hospital admission as recorded in patients’ medical records at hospital discharge. This is direct medical costs (including all types of costs: drugs, medical services, procedures, consumables, tests, bed and room services) paid to the hospital, either by the patient out of pocket or by a third-party payer (such as health insurance). Direct non-medical costs and indirect costs were not included. All costs were converted from Vietnam Dong to US Dollar in 2021 values, with costs incurred in 2019 and 2020 adjusted to the equivalent values in 2021 using Gross Domestic Product deflation rates.

## Data analysis methods

Interrupted time series (ITS) design was used to compare longitudinal changes in the post-intervention period to a hypothetical scenario in which the intervention did not occur (single group analyses). We then performed a controlled interrupted time series (CITS) analysis that incorporated both control and intervention groups into an ITS model (multiple group analyses). Segmented regression models were used to estimate the effects of the intervention for both ITS and CITS. Specifically, antibiotic non-susceptibility was modelled using logistic regression, while other outcomes were modelled using linear regressions.

Single group analyses: Perform the below steps separately for the intervention and the control time series

The ITS model is expressed in the following equation:
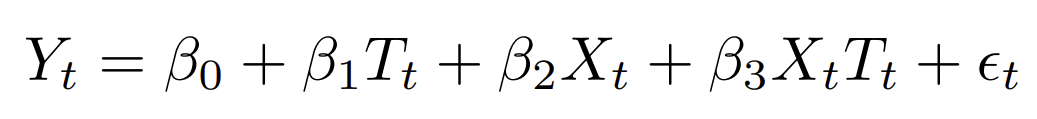
 (1)

- *Y_t_* is the aggregated outcome variable measured at each equally spaced time point *t*,
- *T_t_* is the time since the start of the study,
- *X_t_* is a dummy variable representing the intervention (pre-intervention periods 0, otherwise 1)
- *X_t_T_t_* is an interaction term
- β*_0_* is the intercept or starting level of the outcome variable
- β_1_ is the slope of the outcome variable until the introduction of the intervention
- β_2_ is the change in the level of the outcome that occurs in the period immediately following the introduction of the intervention (compared with the counterfactual)
- β3 is the difference between pre-intervention and post-intervention slopes of the outcome.

Controlled analyses (multiple group analyses): Incorporate the control time series (2).


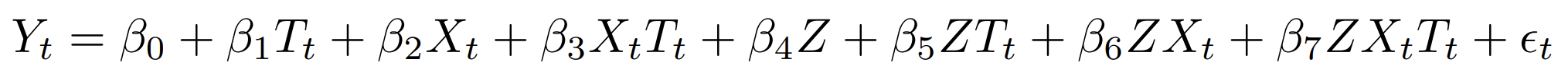
 (2)

- *Z* is a dummy variable to denote the group assignment (intervention or control),
- *ZT_t_*, *ZX_t_*, and *ZX_t_T_t_* are interaction terms among above described variables as in (1)
- β_0_ to β_3_ are for the control group as described in (1)
- β_4_ is the difference in the level (intercept) of the outcome variable between the intervention and control group prior to the intervention
- β_5_ is the difference in the slope (trend) of the outcome variable between intervention and control group prior to the intervention
- β_6_ is the difference between intervention and control group in the level of the outcome variable immediately following introduction of the intervention
- β_7_ is the difference between intervention and control group in the slope (trend) of the outcome variable after initiation of the intervention

The estimate for control group when Z=0:


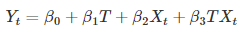
,

where coefficients of level and slope are β2 and β3.

The estimate for intervention group when Z=1:


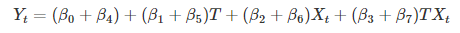
,

where coefficients of level and slope are (β2 + β6) and (β2 + β7).

Autoregressive Integrated Moving Average (ARIMA) models (*p, d, q*) for the time-series datasets the outcome variables (regressing the outcome at time *t* on the values measured at previous time points, not on time), with the following components^[[3]](#endnote-3)^:

- Autoregressive part (AR): regress the outcome on its lagged values, identified by *p* (the order of the AR part)
- Moving average part (MA): regress the outcome on the lagged values of the error of the outcome, identified by *q* (the order of the MA part)
- Integrated: if the time series does not meet the requirement for stationarity, differencing is required to ensure meaningful predictions; by calculating the difference between adjacent observations of the outcome, identified by d (the degree of non-seasonal differencing)
- Seasonal model: regress the outcome on its lagged values at a regular time interval (the season), required when there is variation in the time series of a fixed or known frequency, occurring at regular time intervals, such as time of year or day of the week; in this case, a separate ARIMA model for seasonality is required with the same above 3 components.

Segmented regression model assumptions were checked by examining the residuals, particularly temporal correlation using ACF/PACF plots and Ljung-Box test (as implemented in the checkresiduals() function from the forecast R package). For linear segmented regression, an ARIMA model was added to the regression model to adjust for non-stationarity, autocorrelation, and seasonality. ARIMA model selection was performed using an automated process in the R function auto.arima(). For logistic regression, the models were first run without any lags of antibiotic non-susceptibility proportions. In case of auto-correlation in the simulated residuals, the models were re-run after having added lags of antibiotic non-susceptibility proportion as suggested by the ACF plots. In case of presence of overdispersion in the simulated residuals (test implemented in the check_overdispersion() function of the performance R package^[[4]](#endnote-4)^), the logistic regression models were re-run replacing the binomial distribution by a quasibinomial. Finally, for all the models with less than 10 events (antibiotic use or antibiotic non-susceptibility) or non-events per co-variable, the model was re-run with L2 regularization (ridge) in order to avoid biases and large confidence intervals for the parameter estimates^[[5]](#endnote-5)^. The optimal value of the shrinkage parameter was searched by cross-validation as implemented by the cv.glmnet() function of the glmnet R package. All analyses were conducted in R (v.4.3.1; R Core Team 2022). Results of model checking are presented in Table S20-S23.

## Table S4. TREND Statement Checklist

| **Paper Section/ Topic** | **Item No** | **Descriptor** | **Reported?** | |
| --- | --- | --- | --- | --- |
|  |  |  | Yes/No | Page |
| **Title and Abstract** |  |  |  |  |
| Title and Abstract | 1 | Information on how unit were allocated to interventions | Yes | 3-4 |
|  |  | Structured abstract recommended | Yes | 3-4 |
|  |  | Information on target population or study sample | Yes | 3-4 |
| **Introduction** |  |  |  |  |
| Background | 2 | Scientific background and explanation of rationale | Yes | 5-6 |
|  |  | Theories used in designing behavioral interventions | Yes | 6 |
| **Methods** |  |  |  |  |
| Participants | 3 | Eligibility criteria for participants, including criteria at different levels in recruitment/sampling plan (e.g., cities, clinics, subjects) | Yes | 6-7 |
|  |  | Method of recruitment (e.g., referral, self-selection), including the sampling method if a systematic sampling plan was implemented | Yes | 7 |
|  |  | Recruitment setting | No |  |
|  |  | Settings and locations where the data were collected | Yes | 6-7 |
| Interventions | 4 | Details of the interventions intended for each study condition and how  and when they were actually administered, specifically including: | Yes | 8-9 |
|  |  | Content: what was given? | Yes, i.e. PAF activity, training | 8-9 |
|  |  | Delivery method: how was the content given? | Yes | 8 |
|  |  | Unit of delivery: how were the subjects grouped during delivery? | By study group as described in the methods | 8-9 |
|  |  | Deliverer: who delivered the intervention? | Yes | 8-9 |
|  |  | Setting: where was the intervention delivered? | Yes | 8-9 |
|  |  | Exposure quantity and duration: how many sessions or episodes or events were intended to be delivered? How long were they intended to last? | This was described in the Supplemental Data | Suppl Data |
|  |  | Time span: how long was it intended to take to deliver the  intervention to each unit? | Yes | 8-9 |
|  |  | Activities to increase compliance or adherence (e.g., incentives) | Yes | 8-9 |
| Objectives | 5 | Specific objectives and hypotheses | Yes | 6 |
| Outcomes | 6 | Clearly defined primary and secondary outcome measures | Yes | 9-11 |
|  |  | Methods used to collect data and any methods used to enhance the quality of measurements | Yes | 9-11 |
|  |  | Information on validated instruments such as psychometric and biometric properties | We used WHO defined indicators for antibiotic use and resistance, and commonly used indicators for mortality and length of stay | 9-11 |
| Sample size | 7 | How sample size was determined and, when applicable, explanation of any  interim analyses and stopping rules | All inpatients in the study wards during evaluation periods were included. | 7 |
| Assignment Methods | 8 | Unit of assignment (the unit being assigned to study condition, e.g.,  individual, group, community) | Yes | 7 |
|  |  | Method used to assign units to study conditions, including details of any restriction (e.g., blocking, stratification, minimization) | By cluster: all inpatients in each of the study ward were included. | 7 |
|  |  | Inclusion of aspects employed to help minimize potential bias induced due to non-randomization (e.g., matching) | Interrupted time-series, ARIMA method, including a control group | 11-12 |
| Blinding (masking) | 9 | Whether or not participants, those administering the interventions, and  those assessing the outcomes were blinded to study condition assignment; if so, statement regarding how the blinding was accomplished and how it was assessed. | No, it was not possible to apply masking in the clinical setting. |  |
| Unit of Analysis | 10 | Description of the smallest unit that is being analyzed to assess  intervention effects (e.g., individual, group, or community) | Individual | 11-12 |
|  |  | If the unit of analysis differs from the unit of assignment, the analytical  method used to account for this (e.g., adjusting the standard error  estimates by the design effect or using multilevel analysis) | NA |  |
| Statistical Methods | 11 | Statistical methods used to compare study groups for primary methods  outcome(s), including complex methods of correlated data | Controlled ITS/ARIMA | 11-12 |
|  |  | Statistical methods used for additional analyses, such as a subgroup  analyses and adjusted analysis | Descriptive summary of all data | 11-12 |
|  |  | Methods for imputing missing data, if used | Not used |  |
|  |  | Statistical software or programs used | Yes | 12 |
| **Results** |  |  |  |  |
| Participant flow  Recruitment  Baseline Data | 12 | Flow of participants through each stage of the study: enrollment,  assignment, allocation, and intervention exposure, follow-up, analysis (a  diagram is strongly recommended | Yes | 12-13, Figure 1 |
|  |  | Enrollment: the numbers of participants screened for eligibility,  found to be eligible or not eligible, declined to be enrolled, and  enrolled in the study | NA, we included all patients in the selected study wards in the analysis using routine data extracted from hospital information system. |  |
|  |  | Assignment: the numbers of participants assigned to a study  condition | Yes. Assigned to each study group | 12-13,  Figure 1 |
|  |  | Allocation and intervention exposure: the number of participants  assigned to each study condition and the number of participants  who received each intervention | Yes. Allocated to each study group | 12-13,  Figure 1 |
|  |  | Follow-up: the number of participants who completed the follow-up or did not complete the follow-up (i.e., lost to follow-up), by study condition | We extracted full hospitalization course of all inpatients from admission to discharge date |  |
|  |  | Analysis: the number of participants included in or excluded from  the main analysis, by study condition | All inpatients were included | 12-13,  Figure 1 |
|  |  | Description of protocol deviations from study as planned, along with reasons | None |  |
|  | 13 | Dates defining the periods of recruitment and follow-up | Specified dates of time series | 11, Figure 1 |
|  | 14 | Baseline demographic and clinical characteristics of participants in each study condition | Yes | Table 1 and Supplementary Data Section C |
|  |  | Baseline characteristics for each study condition relevant to specific  disease prevention research | Yes | Table 1 and Supplementary Data Section C |
|  |  | Baseline comparisons of those lost to follow-up and those retained, overall and by study condition | NA, all included |  |
|  |  | Comparison between study population at baseline and target population of interest | We compared baseline patient data by clinical wards included and not included | Table 1 and Supplementary Data Section C |
| Baseline  equivalence | 15 | Data on study group equivalence at baseline and statistical methods used to control for baseline differences | Yes,  Controlled ITS | Table 1 and Supplementary Data Section C |
| Numbers analyzed | 16 | Number of participants (denominator) included in each analysis for each study condition, particularly when the denominators change for different outcomes; statement of the results in absolute numbers when feasible | Yes | Table 1 and Supplementary Data Section C |
|  |  | Indication of whether the analysis strategy was “intention to treat” or, if not, description of how non-compliers were treated in the analyses | NA, all inpatients were included. |  |
| Outcomes and estimation | 17 | For each primary and secondary outcome, a summary of results for each estimation study condition, and the estimated effect size and a confidence interval to indicate the precision | Yes | 14-16 |
|  |  | Inclusion of null and negative findings | Yes | 14-16 |
|  |  | Inclusion of results from testing pre-specified causal pathways through which the intervention was intended to operate, if any | NA, we did not specifically test for causal pathways but use the control group to control for potential confounders in the interrupted time-series design |  |
| Ancillary analyses | 18 | Summary of other analyses performed, including subgroup or restricted analyses, indicating which are pre-specified or exploratory | Yes | 14-16 |
| Adverse events | 19 | Summary of all important adverse events or unintended effects in each study condition (including summary measures, effect size estimates, and confidence intervals) | Mortality and cost of hospitalization are considered | 16 |
| **Discussion** |  |  |  |  |
| Interpretation | 20 | Interpretation of the results, taking into account study hypotheses, sources of potential bias, imprecision of measures, multiplicative analyses, and other limitations or weaknesses of the study | Yes | 16-19 |
|  |  | Discussion of results taking into account the mechanism by which the intervention was intended to work (causal pathways) or alternative mechanisms or explanations | Yes | 16-19 |
|  |  | Discussion of the success of and barriers to implementing the intervention, fidelity of implementation | Yes | 16-19 |
|  |  | Discussion of research, programmatic, or policy implications | Yes | 19 |
| Generalizability |  | Generalizability (external validity) of the trial findings, taking into account the study population, the characteristics of the intervention, length of follow-up, incentives, compliance rates, specific sites/settings involved in the study, and other contextual issues | Discussed in the discussion about the study contribution | 19 |
| Overall Evidence | 22 | General interpretation of the results in the context of current evidence and current theory | Yes | 16-19 |

## Table S5. Assessment of criteria for clinical microbiology data reported in the study using the Microbiology Investigation Criteria for Reporting Objectively (MICRO) framework

| **Item** | **Number** | **Recommendation** | **Assessment of microbiology data reported in this manuscript** |
| --- | --- | --- | --- |
| Methods | | |  |
| Study design | 1* | Specimen types: Describe the types of specimen included, i.e. clinical (e.g. blood cultures) or non-diagnostic surveillance (e.g. admission and other screening swabs to diagnose carriage). If specimens were obtained for diagnostic reasons, clinical syndromes should be described where possible, and specimens/isolates stratified by clinical syndrome. | We used data from microbiology laboratory on the antimicrobial susceptibility testing results (from WHONET software) of routine clinical specimens collected for diagnostic reasons from any patients admitted to the hospital. Data were not recorded on the syndromes of patients tested. Screening samples for carriage is not common practice in these hospitals. |
|  | 2* | Sampling period: State the collection timeframe for specimens yielding isolates for which data is reported, e.g. from MM/YY to MM/YY to be able to identify variability between seasons. | Data reported in this manuscript were for patients admitted to the hospitals in from Jan 2014 to Dec 2021 for hospital 1 and from Dec 2017 to 31 Dec 2021 for hospital 2. |
|  | 3* | Sampling strategy: Describe the strategy for specimen collection, e.g. asymptomatic screening, sampling of all febrile patients, sampling at clinician discretion, sampling of specific patient groups and convenience sampling (e.g. use of isolates from an existing sample repository). Specify whether sampling followed routine clinical practice or was protocol driven. Classify specimens as from community-acquired (CAI) or hospital-acquired (HAI) infections. The definition of HAI used (e.g. HAI defined by specimen collection > 48 h after hospital admission) should be provided and should use ideally an international standard (e.g. US Centers for Disease Control). | Data were from routine sampling of specimens from any patients admitted at the hospital at clinician discretion. We used the definition of HAI as follow to classify the specimens: HAI defined by specimen collection > 48 h after hospital admission. |
|  | 4 | Target organisms: Explicitly state which organisms/organism groups were included in the report. Nomenclature should follow international standards (i.e. using approved genus/species names as summarised in the International Journal of Systematic and Evolutionary Microbiology). Lists of approved bacterial names can be downloaded from Prokaryotic Nomenclature Up-to-Date (<https://www.dsmz.de/bacterial-diversity/prokaryotic-nomenclature-up-to-date.html>) and the List of Prokaryotic Names with Standing in Nomenclature (<http://www.bacterio.net/>). Organisms considered contaminants should be listed, if appropriate (e.g. coagulase negative staphylococci or *Corynebacterium* spp.. | Data was collected for all organisms identified at the routine microbiology lab of each hospital. The 5 species most frequently isolated were kept in the analysis and the present report: *Escherichia coli*, *Staphylococcus aureus*, *Klebsiella* spp., *Acinetobacter* spp., and *Pseudomonas aeruginosa*. |
| Setting | 5* | Geographical setting: Describe the geographical distribution of specimens/patients from which isolates were obtained, at least to a country level, but preferably to a sub-national level or a geoposition. | Isolates reported were from patients in the catchment area of each hospital (in Hai Phong and Dong Thap province, Viet Nam). |
|  | 6* | Clinical setting: Describe the type and level of the healthcare facilities (e.g. primary, secondary, tertiary) from which specimens were obtained. If stating a microbiology laboratory, the centres served by the laboratory should be specified. | Microbiology data were from the routine microbiology laboratory at each of the two provincial hospitals, which are general hospitals in each province in the Viet Nam public healthcare system (consisting of national, provincial and district level hospitals). |
| Laboratory work | 7 | Specimen processing: If applicable, describe specimen collection and handling, processing and sub-culture methods for all types of specimen included. For example, if reporting AST results for blood culture and cerebrospinal fluid culture isolates, the processing of these specimens by the laboratory should be briefly explained, including how specimens are sub-cultured, the media used, incubation conditions and duration. A summary of specimen processing steps (e.g. pre-processing steps, nucleic acid extraction method (if applicable), amplification platform, contamination avoidance strategy) should be provided for molecular-only workflows (e.g. to detect *Mycobacterium tuberculosis* and rifampicin resistance using the Cepheid Xpert MTB/RIF system). | Specimen collection and handling, processing and sub-culture methods followed hospital Standard Operating Procedures developed based on the guidelines issued by the Ministry of Health (MoH) in 2017 (Number 1539), subsequent trainings from MoH and OUCRU, as well as locally published guidance books of medical publishing houses. Blood culture is performed with biphasic blood culture bottles (Nam Khoa Biotek, Vietnam) in hospital 1 and with BacT-Alert automate in hospital 2 (Biomerieux, France). Cultures are performed on homemade media (Oxoid dehydrated culture media, UK) or ready-to-use media (DEKA, Vietnam) for blood agar, chocolate agar, and Mueller-Hinton. |
|  | 8* | Target organism identification: Details of identification methodology should be reported briefly. Where identification databases were used (e.g. bioMerieux API/bioMerieux VITEK-MS/Bruker Biotyper), the version should be specified. In general, all pathogens should be identified to species level. In the case of *Salmonella* species, organisms should be identified to at least the *S. Typhi*, *S. Paratyphi*, or non-typhoidal Salmonella (NTS) level. Strain subtyping methods should be reported according to STROME-ID. | Identification was based on the conventional biochemical methods in both hospitals: API galleries (Biomerieux, France) and with manual phenotypic tests in hospital 1, and with Vitek II (Biomerieux, France; identification database version 9.02) in hospital 2. |
|  | 9* | Antimicrobial susceptibility testing: Describe the antimicrobial susceptibility testing methods used, internal quality control processes and their interpretation, with reference to a recognised international standard, e.g. CLSI, EUCAST. Where an international standard was followed, the specific edition(s) of guidelines used should be referenced. Deviations from standard methodology should be described, along with evidence of validation. Handling of any changes to interpretative criteria during the sampling period should be documented. State whether the raw AST data (zone diameters and/or minimum inhibitory concentrations) were re-categorised with updated breakpoints or left as-is. | Antimicrobial susceptibility testing methods: disc diffusion at both hospitals; Automated Vitek was also used by hospital 2;  Routine AST data at the microbiology labs was entered into WHONET by hospital technicians. Extracted files were converted to WHONET format using BacLink, a free tool included in WHONET. AST results were categorised in S/I/R according to CLSI 2022 guidelines for the present report. |
|  | 10 | Additional tests performed to identify resistance mechanisms: Describe the testing methods used for adjunctive/confirmatory antimicrobial susceptibility tests, such as enzymatic/molecular assays (e.g. Xpert MTB/RIF, mecA PCR) and inducible resistance assays, with reference to a recognised international standard, where available. Where an international standard was followed, the specific edition of guidelines used should be referenced. Deviations from standard methodology should be described, along with evidence of validation. | Not applicable. |
|  | 11* | Antimicrobial resistance definitions: Define resistance for each antimicrobial class (i.e. are isolates in the ‘intermediate’ category included within ‘susceptible’ or ‘resistant’ or analysed as a distinct category). If using the term, define MDR (e.g. ≥ 1 agent in ≥ 3 classes tested). For each organism type, an MDR test panel must be defined, consisting of the minimum panel of individual antimicrobial agents/classes against which an isolate must be tested for that isolate to be considered tested for MDR status. Antimicrobials to which an organism is intrinsically resistant cannot be part of the test panel or contribute to MDR status. | Definitions of resistance to specific antibiotic/antibiotic subgroups were as follows, after a consensus discussion among clinical microbiologists at OUCRU. We define MDR as non-susceptible to ≥1 agent in ≥3 antimicrobial categories following the criteria proposed by Magiorakos et al^[[6]](#endnote-6)^  ***E. coli or Klebsiella* spp.*:***  - 3GC (ceftriaxone or ceftazidime)  - aminoglycoside (gentamicin and one of amikacin / tobramycin)  - fluoroquinolones (ciprofloxacin),  - carbapenems (one of three: ertapenem/ imipenem/ meropenem; doripenem was not tested at these two hospitals)  ***P. aeruginosa or Acinetobacter* spp.*:***  - 3GC (ceftazidim)  - aminoglycosides (one of amikacin/tobramycin)  - fluoroquinolones (ciprofloxacin)  - carbapenems (one of two: imipenem/meropenem, doripenem was not tested at these two hospitals excluding ertapenem)  - piperacillin / tazobactam  - aztreonam  - colistin   (tigecycline was not tested in these two hospitals)    ***S. aureus:***  - MRSA (oxacillin or cefoxitin) |
| Quality assurance | 12* | External quality assurance: State whether the microbiology laboratory participates in an external quality control programme and, if so, provide scheme details. Examples include the UK National External Quality Assurance Scheme ([www.ukneqasmicro.org.uk](http://www.ukneqasmicro.org.uk/)) and the American College of Pathologists External Quality Assurance/Proficiency Testing Program (<https://www.cap.org/>) | Hospital 1: participated in two external quality control programs: UK NEQAS and Ho Chi Minh City quality assurance program  Hospital 2: participated in two external quality control programs: UK NEQAS and Hanoi Medical University – Viet Nam |
|  | 13 | Accreditation: State whether the laboratory is accredited through a national or international body (e.g. the International Standards Organisation, ISO) and specify which assays are covered in the accreditation. | No ISO certification during the study periods. Hospital 2 has participated in the Strengthening Laboratory Management Toward Accreditation (SLMTA) program. |
| Bias | 14* | Duplicate and sequential isolates: The strategy for accounting for duplicate and sequential isolates from the same patient should be clearly detailed. Duplicate isolates are multiple isolates of the same phenotypic organism (i.e. same species and same resistance profile) from the same patient on the same date cultured either from the same clinical specimen, or from two separate clinical specimens, such as blood and CSF. Sequential isolates are isolates of the same phenotypic organism from the same patient at different dates, such as blood cultures taken on different dates. Various strategies for the handling of duplicate and sequential isolates exist, and the strategy used should be transparent as it will bias pooled resistance results. For example, inclusion of all isolates (the ‘all isolate strategy’) has been shown to shift pooled resistance proportions toward greater resistance, whilst inclusion of only the first isolate per patient (the ‘first isolate strategy’) or only the first isolate per infection episode (the ‘episode-based strategy’) will shift pooled results toward susceptibility. | WHONET data were de-duplicated, one isolate representing one patient for each type of specimens. Only the first isolate per patient, per pathogen, per reporting period (i.e. for the whole year 2019) at each hospital was included. |
| Results | | |  |
|  | 15* | Population: Describe the demographics of the population from which clinical specimens and subsequent isolates have been obtained, disaggregating age and gender data. | Population: patients admitted to each hospital in the study calendar years and stratified by clinical wards. Data on the isolates were described with age and gender information in the Supplementary Figure 2S. |
|  | 16* | Denominators: Patient and isolate denominators should be used appropriately to ensure clarity regarding the numbers included in each analysis. Of particular importance is the reporting of resistance where first- and second-line AST panels were used (i.e. not all isolates of a particular species were tested against all agents). For drugs where only a subset of isolates were tested, reporting of a percentage without the numbers of isolates tested/resistant may be highly misleading. | Denominators were presented for each percentage described. |
|  | 17 | Site/place of acquisition: AST data from CAI and HAI should be reported and analysed separately. | We presented results for all isolates and for HAI isolates following the definition of HAI to classify the specimens: HAI defined by specimen collection > 48 h after hospital admission. |
|  | 18* | Reporting resistance proportions for single agent and class resistance: Proportions of resistant isolates should be reported as number of isolates susceptible or resistant to a given antimicrobial agent/class out of actual number of isolates tested for susceptibility to that agent/class. | We presented both resistance proportions for single agent and class resistance for each of the key pathogens either in the main text or in the Supplementary Tables. |
|  | 19 | Reporting multidrug resistance proportions: If defined, the proportion of MDR isolates should be expressed as the number of MDR isolates out of the number of isolates tested (i.e. the number undergoing the MDR test panel specific to that organism). Single agent/class resistance should be always be reported, regardless of MDR reporting. | We presented both resistance proportions for single agent and class resistance and MDR for each of the key pathogens either in the main text or in the Supplementary Tables. |
| Discussion | | |  |
| Limitations | 20 | Discuss any reasons why bias may have been introduced into the reported data, due to patient/specimen selection, isolation of organisms, or otherwise. Consider factors which may have either introduced bias into the types of organisms isolated or the antimicrobial susceptibility profiles, e.g. receipt of antimicrobials prior to specimen collection will reduce the yield of certain species and also select for more resistant organisms. | We reported the baseline AST data of the study wards in each hospital using the routine data collected for diagnostic reasons. No diagnostic stewardship program existed at baseline in either one of the 2 hospitals. Therefore, it is expected that the clinicians’ testing habits have impacted the selection of samples and isolates subsequently tested for antimicrobial susceptibility. Because reimbursement of laboratory test is based on strict clinical criteria, the isolates may not represent the population of pathogens, but this bias remains constant between wards, between hospitals, and with time. |
| *Core ‘must include’ items | | | |

# **C. Patient characteristics and summary outcome indicators**

## Figure S3. Number of patients by subgroups, mortality per 1000 admissions and number of microbiological isolates over time in each hospital in eight study wards at each hospital (vertical lines indicate the start of AMS intervention)

|  | Hospital 1 | Hospital 2 |
| --- | --- | --- |
| Number of patients by ICD10 diagnosis group | 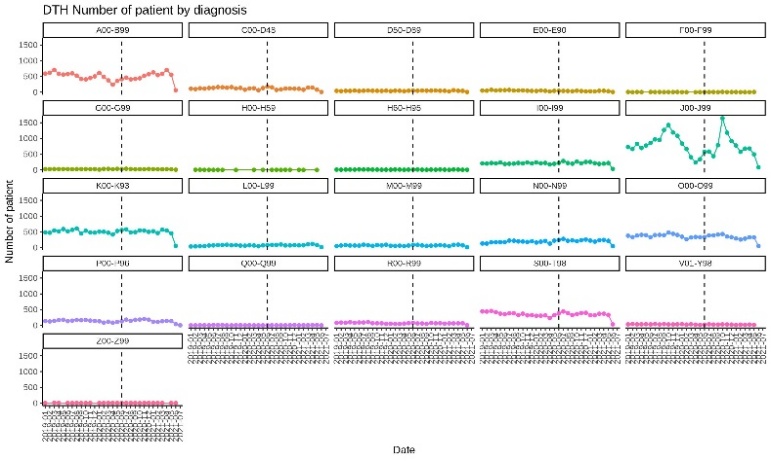 | 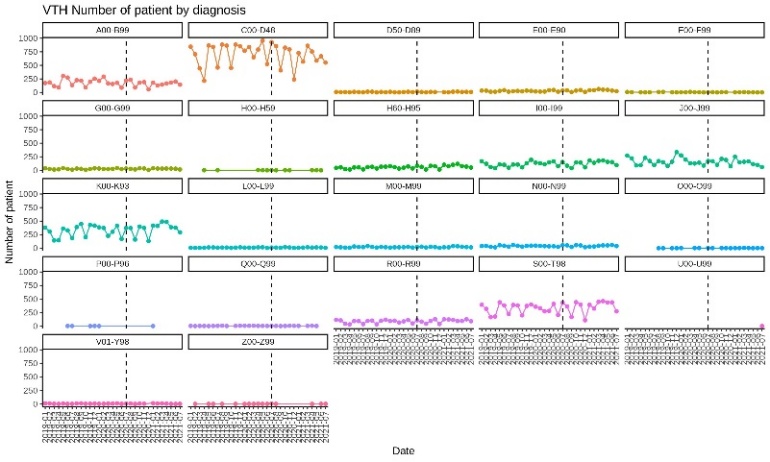 |
| Number of all patients by study ward | 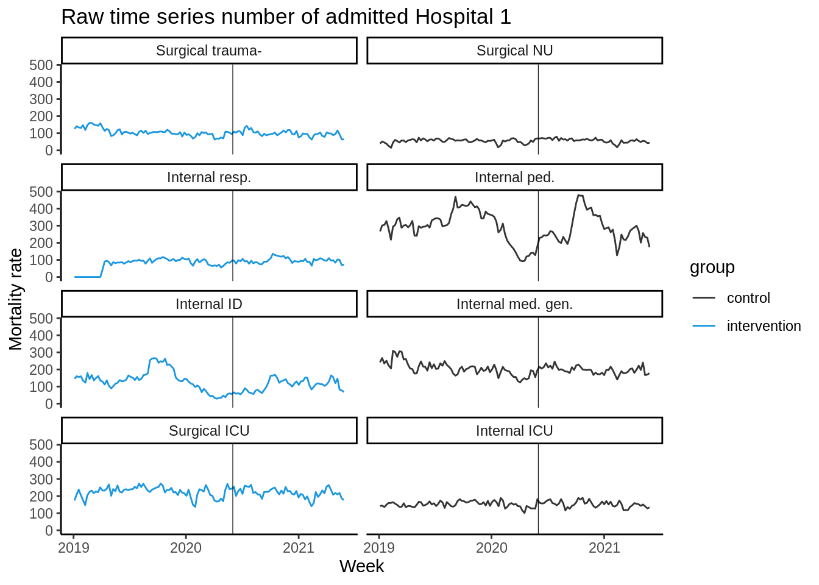 | 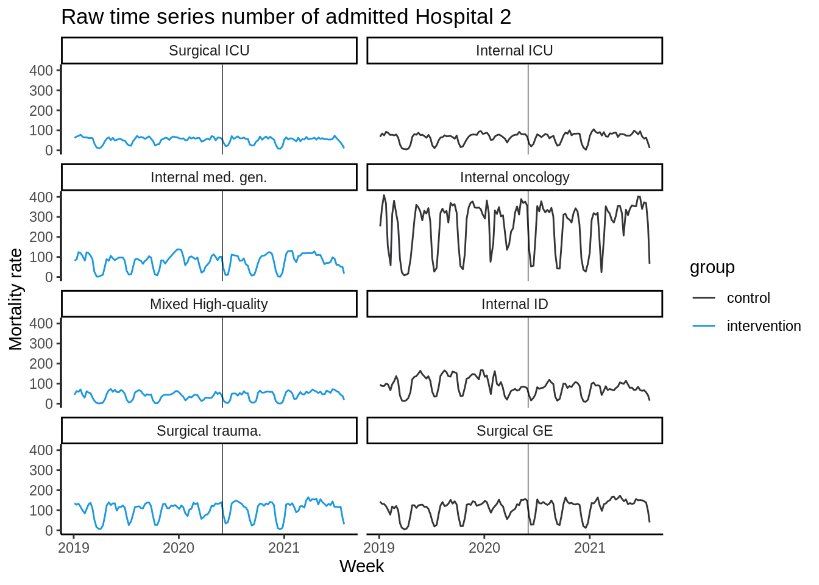 |
| Mortality per 1000 admissions | 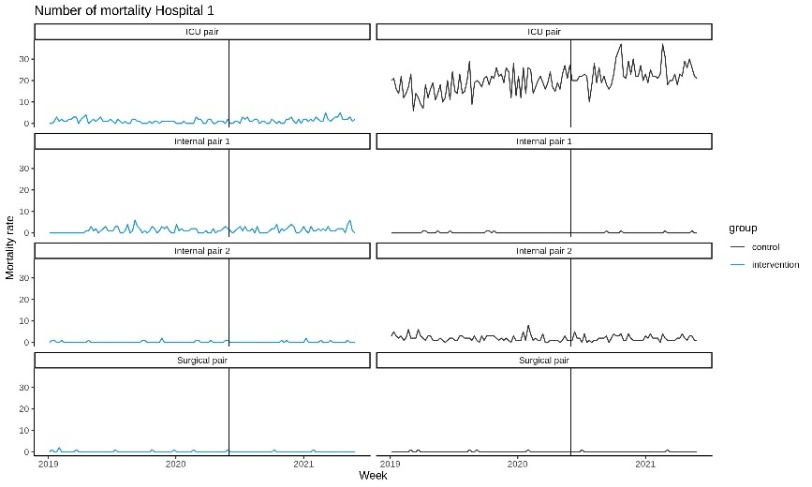 | 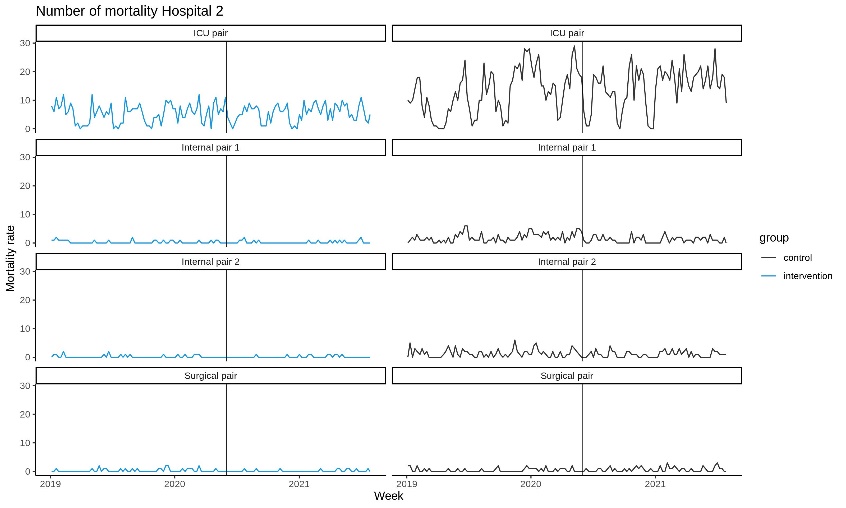 |
| Monthly distributions of isolates for common bacterial pathogens in routine microbiology | | |
| *E. coli*  *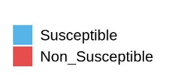* | 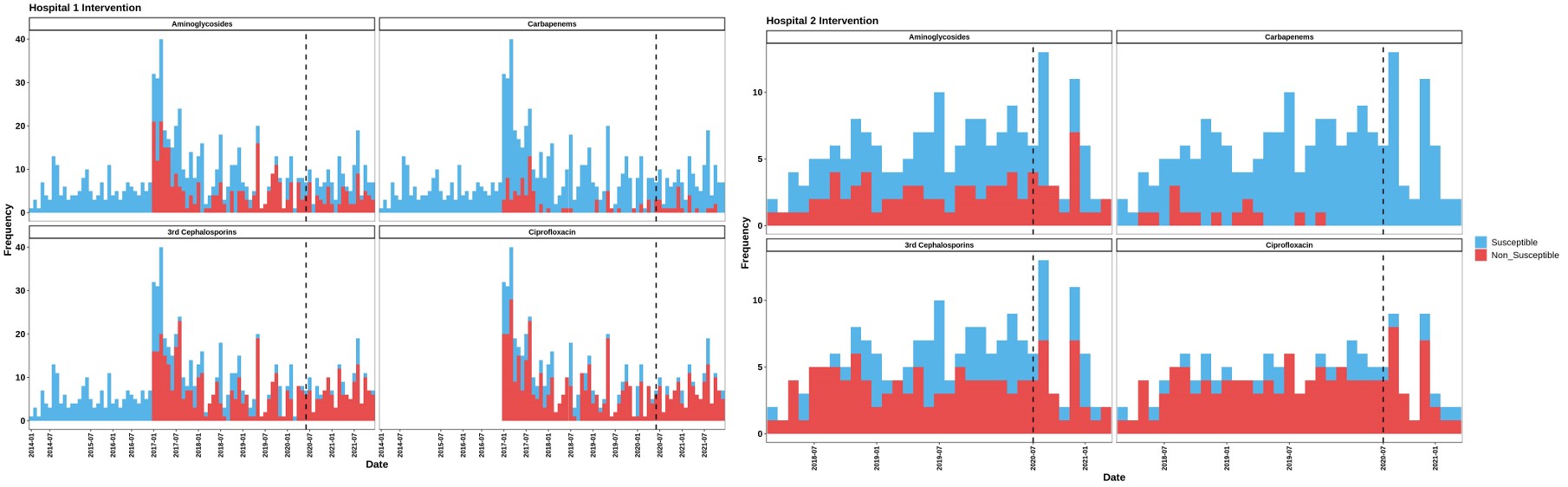 | |
| *Klebsiella* spp.  *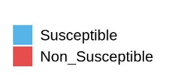* | 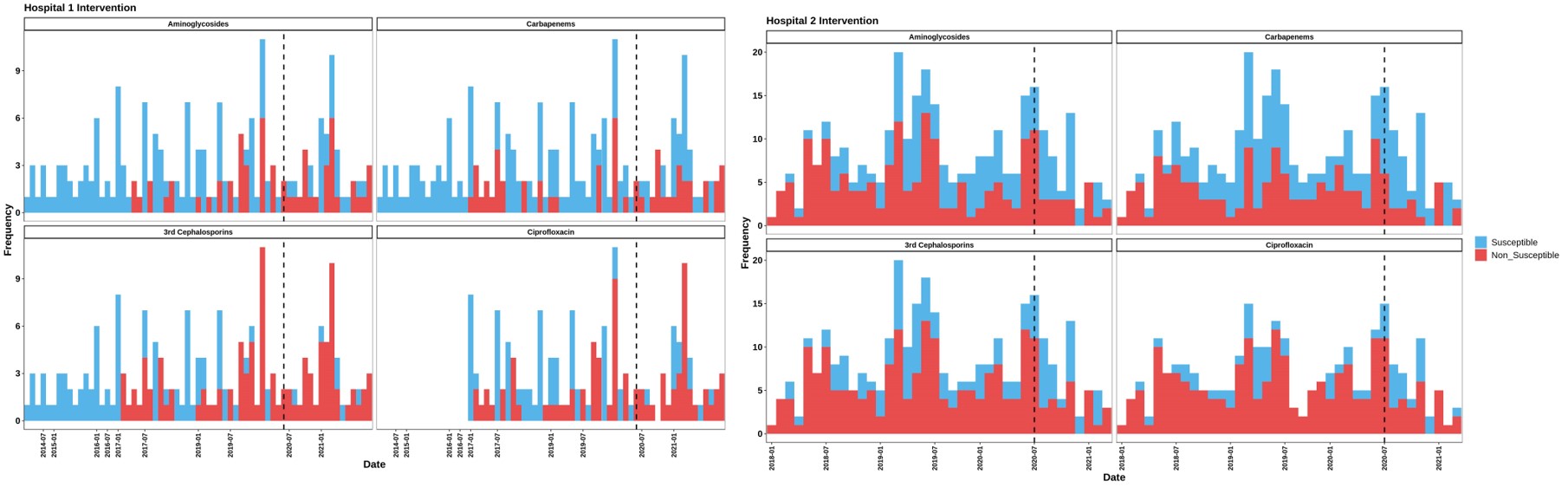 | |
| *Acinetobacter* spp.  *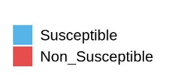* | 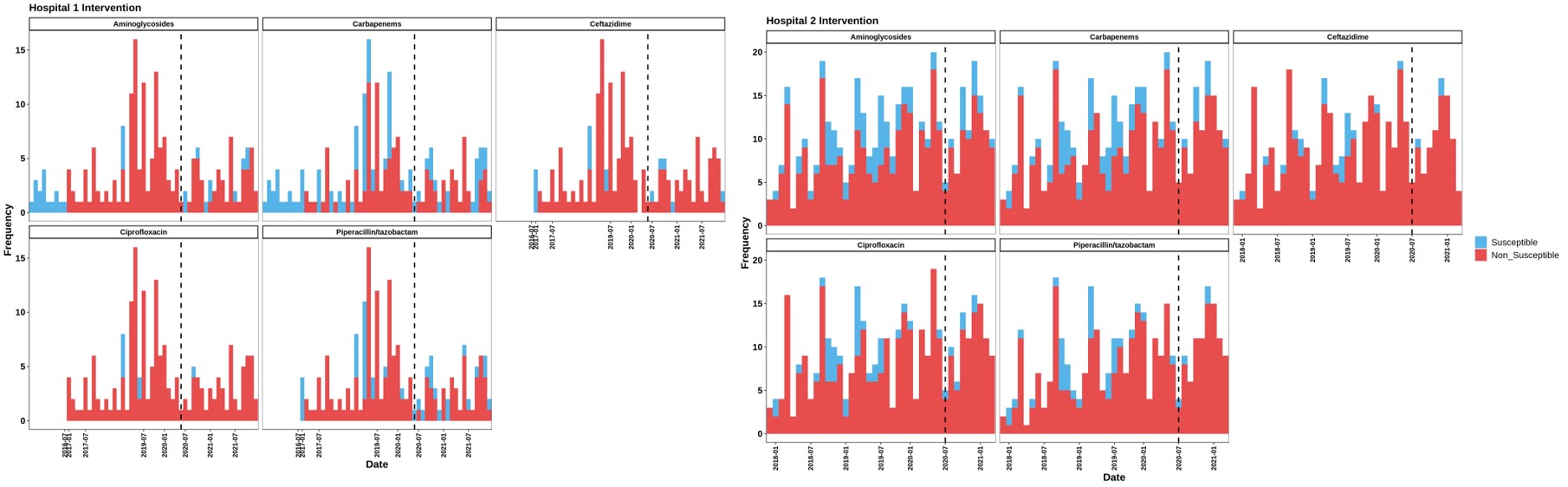 | |
| *P. aeruginosa*  *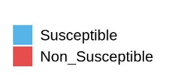* | 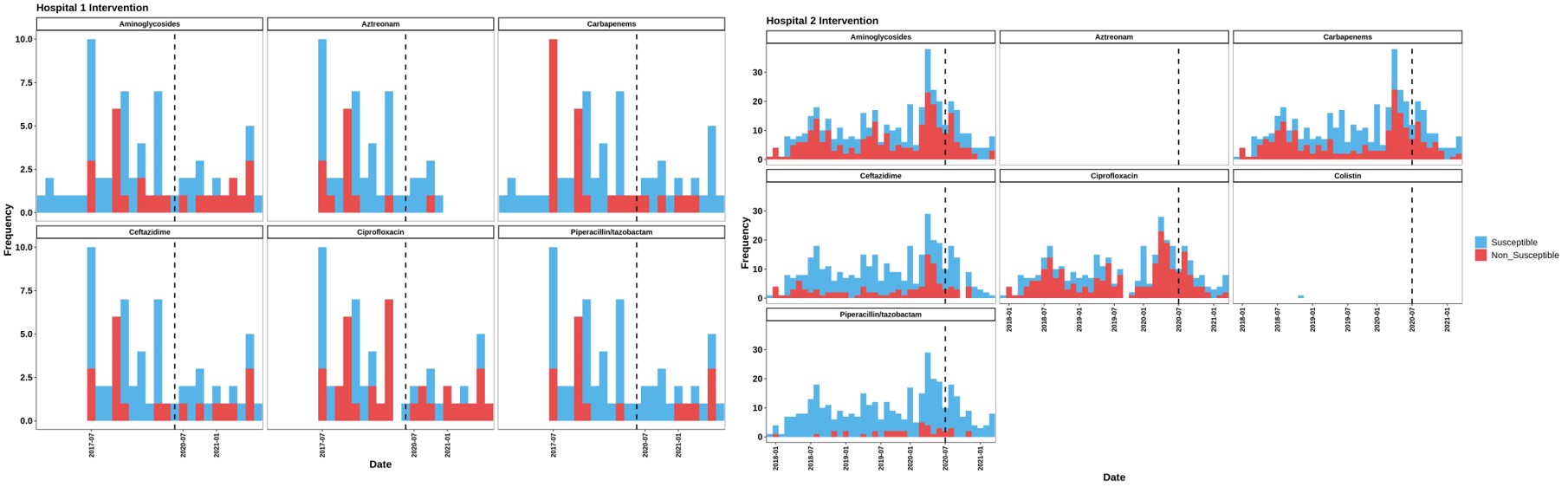 | |
| *S. aureus*  *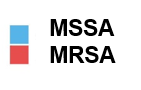* | 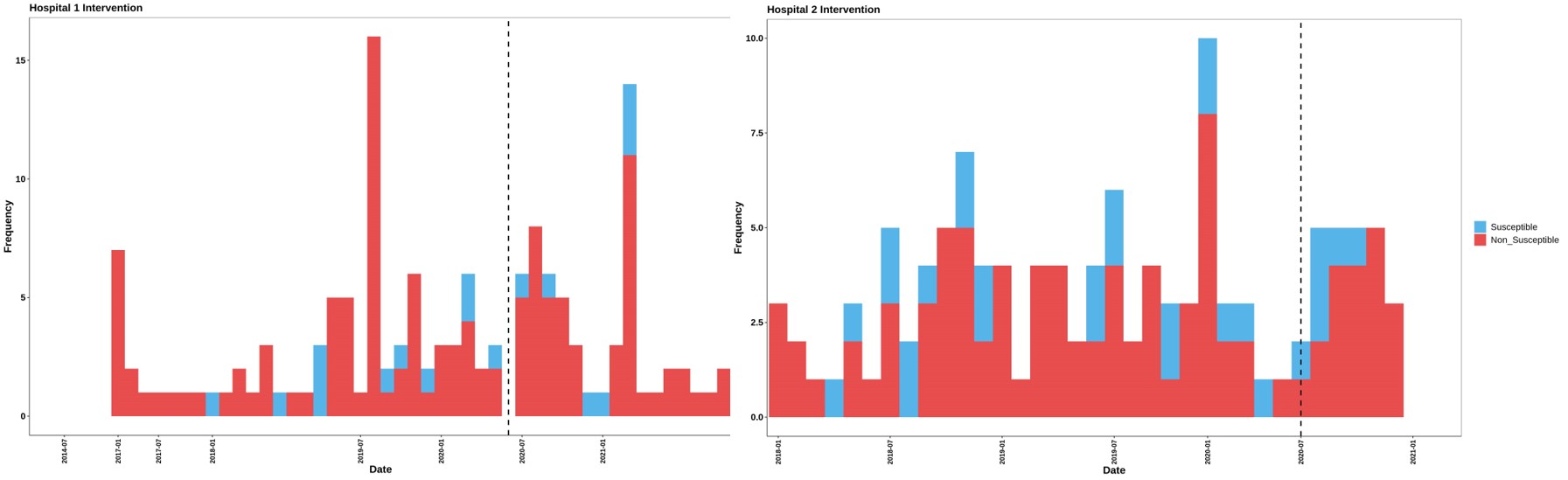 | |

## Table S6. Summary of variables before and after the start of AMS intervention of the intervention and control group in two hospitals – more detailed results

| **Study variables** | **Hospital 1** | | | | **Hospital 2** | | | |
| --- | --- | --- | --- | --- | --- | --- | --- | --- |
|  | **Intervention** | | **Control** | | **Intervention** | | **Control** | |
|  | **Before** | **After** | **Before** | **After** | **Before** | **After** | **Before** | **After** |
| ***Patient characteristics*** | | | | | | | | |
| **Total number of patients^*^** | 27,341 | 18,282 | 30,245 | 21,199 | 10,994 | 7,848 | 15,403 | 9,998 |
| By ward pair^†^ |  |  |  |  |  |  |  |  |
| ICU pair | 15,569 | 10,644 | 8,359 | 6,126 | 2,256 | 1,444 | 2,799 | 2,125 |
| Surgical pair | 4,979 | 3,462 | 2,081 | 1,574 | 3,700 | 2,730 | 4,575 | 3,190 |
| Internal pair 1 | 3,130 | 2,954 | 13,146 | 8,933 | 3,472 | 2,322 | 4,289 | 2,917 |
| Internal pair 2 | 6,602 | 3,418 | 9,074 | 6,156 | 1,806 | 1,450 | 4,220 | 2,040 |
| **Gender** (male, %) |  |  |  |  |  |  |  |  |
| ICU pair | 5638/15510 (36%) | 4052/10614 (38%) | 4197/7933 (53%) | 3099/5863 (53%) | 1471/2209  (67%) | 915/1444  (63%) | 1762/2755 (64%) | 1371/2125 (65%) |
| Surgical pair | 3202/4890 (65%) | 2175/3441 (63%) | 1330/2057 (65%) | 1011/1562 (65%) | 2335/3626  (64%) | 1776/2730 (65%) | 2595/4506 (58%) | 1765/3190 (55%) |
| Internal pair 1 | 1663/3116 (53%) | 1571/2937 (53%) | 7374/13012 (57%) | 5049/8888 (57%) | 1628/3432  (47%) | 1022/2322 (44%) | 2208/4264 (52%) | 1539/2917 (53%) |
| Internal pair 2 | 3614/6519 (55%) | 1865/3412 (55%) | 4329/8923 (49%) | 2898/6123 (47%) | 873/1787  (49%) | 686/1450  (47%) | 2403/4173 (58%) | 1285/2040 (63%) |
| **Age** (median, 1^st^ and 3^rd^ quantile) |  |  |  |  |  |  |  |  |
| ICU pair | 34 (26, 49) | 35 (27, 51) | 67 (52, 79) | 67 (54, 78) | 62 (48, 76) | 63 (50, 75) | 65 (52, 77) | 65 (54, 77) |
| Surgical pair | 40 (25, 55) | 40 (25, 57) | 52 (38, 65) | 54 (38, 64) | 47 (32, 62) | 48.5 (32, 63) | 57 (40, 69) | 58 (41, 70) |
| Internal pair 1 | 69 (58, 81) | 70 (59, 80) | 2 (1, 5) | 2 (1, 5) | 68 (53, 79) | 66 (52, 77) | 61 (53, 69) | 61 (53, 68) |
| Internal pair 2 | 6 (3, 17) | 4 (2, 23) | 59 (43, 72) | 58 (43, 70) | 56 (35, 71) | 57 (37, 71) | 43 (30, 59) | 50 (35, 63) |
| ***Antibiotic use and clinical outcomes*** | | | | | | | | |
| **Patients with antibiotic use** (%) |  |  |  |  |  |  |  |  |
| All study wards | 71.4% | 70.1% | 67.0% | 66.4% | 68.3% | 63.9% | 72.0% | 76.3% |
| ICU pair | 71.1% | 63.9% | 52.0% | 49.5% | 93.7% | 91.6% | 88.6% | 94.3% |
| Surgical pair | 79.1% | 77.5% | 92.0% | 90.0% | 82.5% | 80.7% | 88.7% | 89.8% |
| Internal pair 1 | 79.2% | 77.1% | 80.7% | 82.5% | 37.4% | 26.8% | 50.1% | 50.1% |
| Internal pair 2 | 49.2% | 57.9% | 49.7% | 47.8% | 68.2% | 65.7% | 63.5% | 72.6% |
| **Total number of patient days** |  |  |  |  |  |  |  |  |
| All study wards | 105,466 | 72,082 | 176,587 | 120,392 | 96,544 | 63,348 | 190,187 | 127,256 |
| ICU pair | 22,681 | 16,292 | 27,411 | 19,050 | 18,048 | 10,676 | 20,519 | 14,312 |
| Surgical pair | 28,302 | 17,785 | 15,623 | 12,192 | 38,808 | 25,842 | 38,194 | 28,443 |
| Internal pair 1 | 19,415 | 18,161 | 80,557 | 54,249 | 27,468 | 17,750 | 99,416 | 68,357 |
| Internal pair 2 | 35,068 | 19,844 | 52,996 | 34,901 | 12,220 | 9,080 | 32,058 | 16,144 |
| **Days of antibiotic therapy** (DOT) |  |  |  |  |  |  |  |  |
| All study wards | 84,793 | 63,140 | 160,166 | 112,577 | 53,244 | 31,056 | 68,676 | 54,305 |
| ICU pair | 21,701 | 15,826 | 26,418 | 18,002 | 17,676 | 9,195 | 19,301 | 16,857 |
| Surgical pair | 24,506 | 13,837 | 21,260 | 18,256 | 22,797 | 13,710 | 22,555 | 19,700 |
| Internal pair 1 | 21,362 | 22,148 | 78,815 | 55,133 | 5,914 | 3,369 | 11,681 | 8,370 |
| Internal pair 2 | 17,224 | 11,329 | 33,673 | 21,186 | 6,857 | 4,782 | 15,139 | 9,378 |
| **DOT per 1000 patient days** |  |  |  |  |  |  |  |  |
| All study wards | 804.0 | 875.9 | 907.0 | 935.1 | 551.5 | 490.2 | 361.1 | 426.7 |
| ICU pair | 957 | 971 | 964 | 945 | 979 | 861 | 941 | 1178 |
| Surgical pair | 866 | 778 | 1361 | 1497 | 587 | 531 | 591 | 693 |
| Internal pair 1 | 1100 | 1220 | 978 | 1016 | 215 | 190 | 117 | 122 |
| Internal pair 2 | 491 | 571 | 635 | 607 | 561 | 527 | 472 | 581 |
| **AWaRe classification** (% DOT) |  |  |  |  |  |  |  |  |
| Access | 11.3% | 19.1% | 14.4% | 19.7% | 21.9% | 14.0% | 21.0% | 15.6% |
| Watch | 88.1% | 80.0% | 84.6% | 78.9% | 60.1% | 58.7% | 58.6% | 57.8% |
| Reserve | 0.4% | 0.5% | 0.7% | 0.9% | 1.0% | 1.1% | 3.5% | 4.2% |
| Other | 0.1% | 0.4% | 0.3% | 0.4% | 16.9% | 26.2% | 16.9% | 22.4% |
| **Chemical subgroups** (% DOT)^‡^ |  |  |  |  |  |  |  |  |
| Beta-lactamase resistant penicillin | 0.1% | 1.1% | 1.1% | 2.9% | 1.7% | 1.6% | 1.2% | 1.0% |
| Beta-lactamase sensitive penicillin | - | - | - | - | - | - | 0.0% | 0.0% |
| Carbapenem | 2.0% | 3.8% | 3.2% | 4.9% | 1.2% | 2.4% | 4.2% | 6.7% |
| Combinations of antibacterials | 0.0% | 0.0% | 0.0% | 0.0% | - | - | - | - |
| Combinations of penicillin, incl. beta-lactamase inhibitors | 6.6% | 9.1% | 7.2% | 7.4% | 27.9% | 26.4% | 21.8% | 32.8% |
| Combinations of sulfonamides and trimethoprim, incl. derivatives | 0.3% | 0.5% | 0.0% | 0.1% | 0.9% | 0.6% | 1.6% | 1.1% |
| First-generation cephalosporin | - | - | - | - | - | - | - | 0.0% |
| Fluoroquinolones | 18.7% | 18.5% | 18.4% | 14.8% | 22.6% | 16.5% | 30.2% | 26.5% |
| Fourth-generation cephalosporin | 0.5% | 1.4% | 4.7% | 5.5% | 5.6% | 2.5% | 0.9% | 0.3% |
| Glycopeptide antibacterial | 3.7% | 2.3% | 5.3% | 1.4% | 1.5% | 1.9% | 2.5% | 1.7% |
| Imidazole derivatives | 3.9% | 5.5% | 1.4% | 1.6% | 0.6% | 0.6% | 0.8% | 0.9% |
| Lincosamide | 0.0% | 0.0% | 0.2% | 0.1% | 0.1% | 0.1% | 0.1% | 0.2% |
| Macrolides | 1.4% | 1.4% | 2.6% | 3.9% | 0.1% | 0.3% | 0.9% | 0.8% |
| Aminoglycoside | 1.8% | 3.9% | 6.7% | 9.2% | 1.8% | 2.0% | 1.5% | 2.1% |
| Other antibacterial | 0.0% | 0.0% | 0.1% | 0.2% | 0.5% | 0.7% | 0.2% | 2.4% |
| Penicillin with extended spectrum | 0.0% | 0.0% | 0.0% | 0.1% | 0.8% | 0.6% | 0.5% | 0.4% |
| Polymyxin | 0.4% | 0.5% | 0.6% | 0.8% | 0.8% | 0.7% | 3.4% | 1.8% |
| Second-generation cephalosporin | 13.8% | 5.2% | 5.5% | 5.0% | 6.6% | 16.6% | 4.0% | 6.2% |
| Tetracycline | 0.0% | 0.1% | 0.0% | 0.0% | - | - | - | - |
| Third-generation cephalosporin | 46.6% | 46.7% | 42.9% | 42.1% | 27.3% | 26.4% | 26.2% | 15.1% |
| **In-hospital mortality per 1000**^∏^ |  |  |  |  |  |  |  |  |
| All study wards | 6.7 | 9.6 | 45.2 | 58.8 | 43.8 | 41.7 | 92.3 | 81.5 |
| ICU pair | 5.0 | 8.2 | 160.2 | 199.8 | 194.1 | 213.3 | 368.3 | 382.1 |
| Surgical pair | 1.8 | 0.6 | 2.4 | 1.3 | 6.5 | 3.3 | 7.4 | 11.6 |
| Internal pair 1 | 27.2 | 28.1 | 0.5 | 0.4 | 7.8 | 4.7 | 36.4 | 19.5 |
| Internal pair 2 | 2.0 | 2.0 | 16.2 | 16.6 | 8.9 | 6.9 | 26.1 | 27.0 |
| **Cost of hospitalization**^#^ |  |  |  |  |  |  |  |  |
| All study wards | 238 (311)  190 (72-273) | 252 (332)  196 (84-282) | 194 (302)  101 (56-206) | 202 (330)  107(63-211) | 941 (1286)  509 (249-1064) | 811 (1001)  476 (242-934) | 772 (1089)  417 (201-866) | 826 (988)  527 (269-993) |
| ICU pair | 324 (347)  242 (194-337) | 322 (347)  241 (190-328) | 346 (424)  214(117-396) | 348 (471)  209(113-386) | 2087 (1873)  1626 (850-2652) | 1751 (1404)  1456(769-2337) | 1930 (1883)  1348(607-2568) | 1680 (1590)  1210(597-2146) |
| Surgical pair | 251 (343)  150 (85-321) | 222 (300)  143 (78-282) | 335 (474)  181 (85-400) | 335 (485)  179 (82-412) | 911 (1121)  536 (284-1037) | 794 (941)  511 (267-910) | 641 (682)  448 (245-783) | 713 (635)  564 (325-912) |
| Internal pair 1 | 230 (301)  149 (84-258) | 248 (369)  152 (82-267) | 104 (155)  69 (43-109) | 115 (175)  81 (49-124) | 459 (825)  300 (170-510) | 400 (555)  286 (166-462) | 456 (437)  311 (194-548) | 439 (439)  289 (182-523) |
| Internal pair 2 | 80 (165)  51 (37-74) | 106 (226)  59 (43-87) | 205 (265)  120 (67-242) | 195 (268)  115 (68-226) | 730 (762)  530 (263-913) | 663 (656)  523 (285-811) | 458 (688)  261 (127-515) | 547 (672)  357 (198-639) |

*Total number of study patients in each study group (consisting of 4 corresponding wards) has been de-duplicated for the same patients moving between corresponding wards in the same admission.

†Ward pair = intervention ward versus control ward, including the following pairs:

- ICU pair: surgical versus internal ICU (in both hospitals)
- Surgical pair: Hospital 1: traumatology versus nephron-urology; Hospital 2: traumatology versus gastroenterology
- Internal pair 1: Hospital 1: respiratory versus paediatrics; Hospital 2: general internal medicine (high-quality services) versus infectious diseases;
- Internal pair 2: Hospital 1: infectious diseases versus general internal medicine; Hospital 2: general internal medicine (normal services) versus oncology

‡ Cells with 0.0% indicate those with very small non-zero amount; cells with “-“ indicate those with zero amount

^∏^ In-hospital mortality was calculated as those with outcome recorded as “death” and “going home to die” out of the patients admitted to the corresponding study wards (expressed as per 1000 admissions), regardless of the wards the patients were transferred from or transferred to.

# Costs are presented in US Dollar mean (standard deviation) and median (interquartile range) of hospitalization, calculated for individual patients. For converting from Vietnam Dong to US Dollar, we used the average exchange rate of 2019 to 2021 (2019=23050.24, 2020= 23208.37, 2021=23159.78) available at: <https://data.worldbank.org/indicator/PA.NUS.FCRF?locations=VN&name_desc=true>

## Table S7. Number of patients and proportions of patients with antibiotic use by main ICD10 diagnosis groups in intervention and control groups in two hospitals before and after the start of AMS implementation

| **ICD10 Diagnosis Group** | **Time period** | **Hospital 1** | | | | **Hospital 2** | | | |
| --- | --- | --- | --- | --- | --- | --- | --- | --- | --- |
|  |  | **Intervention** | | **Control** | | **Intervention** | | **Control** | |
|  |  | Number of patients | Proportion of antibiotic use | Number of patients | Proportion of antibiotic use | Number of patients | Proportion of antibiotic use | Number of patients | Proportion of antibiotic use |
| Certain infectious and parasitic diseases | Before | 2990 | 70.5% | 4714 | 84.0% | 429 | 79.3% | 2406 | 68.4% |
|  | After | 2700 | 61.9% | 2910 | 89.9% | 293 | 78.2% | 1260 | 73.5% |
| Diseases of the circulatory system | Before | 213 | 73.7% | 2811 | 34.5% | 1314 | 39.0% | 345 | 83.5% |
|  | After | 208 | 76.0% | 2241 | 31.1% | 976 | 34.8% | 294 | 88.1% |
| Diseases of the digestive system | Before | 3695 | 88.3% | 3872 | 26.2% | 1021 | 74.6% | 3845 | 86.4% |
|  | After | 2530 | 89.6% | 3029 | 24.4% | 774 | 72.5% | 2806 | 88.9% |
| Diseases of the genitourinary system | Before | 1196 | 64.7% | 2115 | 84.0% | 345 | 90.4% | 222 | 82.9% |
|  | After | 945 | 61.1% | 2014 | 76.3% | 229 | 85.2% | 169 | 89.3% |
| Diseases of the respiratory system | Before | 2880 | 81.0% | 9032 | 95.1% | 1090 | 91.8% | 1387 | 91.0% |
|  | After | 2073 | 86.7% | 6052 | 95.6% | 455 | 91.6% | 935 | 96.3% |
| Diseases of the skin and subcutaneous tissue | Before | 319 | 84.6% | 617 | 65.6% | 82 | 85.4% | 53 | 84.9% |
|  | After | 323 | 75.9% | 513 | 57.9% | 72 | 83.3% | 36 | 86.1% |
| Injury, poisoning and certain other consequences of external causes | Before | 4802 | 79.9% | 367 | 28.6% | 4149 | 83.8% | 279 | 72.8% |
|  | After | 3531 | 77.7% | 343 | 27.4% | 3072 | 80.8% | 214 | 81.8% |
| Neoplasms | Before | 1223 | 81.2% | 644 | 58.9% | 420 | 70.5% | 4608 | 55.1% |
|  | After | 804 | 79.0% | 426 | 59.2% | 254 | 70.5% | 2934 | 53.2% |
| Symptoms, signs and abnormal clinical and laboratory findings, not elsewhere classified | Before | 267 | 85.4% | 965 | 73.9% | 318 | 74.5% | 987 | 90.1% |
|  | After | 164 | 82.9% | 569 | 70.8% | 239 | 72.0% | 748 | 95.3% |

## Table S8. Summary of bacterial isolates reported from the routine microbiological culture at two hospitals: all isolates and hospital-acquired (HA) isolates

|  | **Hospital 1** | | | | | | **Hospital 2** | | | | | |
| --- | --- | --- | --- | --- | --- | --- | --- | --- | --- | --- | --- | --- |
|  | **All hospital wards** | | **Intervention** | | **Control** | | **All hospital wards** | | **Intervention** | | **Control** | |
|  | **All isolates** | **HA isolates** | **All isolates** | **HA isolates** | **All isolates** | **HA isolates** | **All isolates** | **HA isolates** | **All isolates** | **HA isolates** | **All isolates** | **HA isolates** |
| **All isolates** | **11610** | **8779** | **4277** | **1755** | **7333** | **3495** | **14316** | **7062** | **3128** | **1938** | **2666** | **1079** |
| Blood/CSF isolates | 2154 | 1360 | 237 | 155 | 1708 | 844 | 3803 | 828 | 648 | 346 | 1093 | 301 |
| ICU isolates | 7601 | 3152 | 3570 | 1219 | 3286 | 1924 | 5950 | 3563 | 2345 | 1613 | 1766 | 800 |
| ***E.coli* isolates** | **7224** | **2942** | **2393** | **802** | **1819** | **982** | **2815** | **989** | **472** | **189** | **414** | **100** |
| Blood/CSF isolates | 335 | 241 | 23 | 23 | 264 | 138 | 1121 | 282 | 206 | 62 | 266 | 48 |
| ICU isolates | 3388 | 1221 | 2268 | 713 | 930 | 507 | 703 | 257 | 264 | 134 | 202 | 38 |
| ***Klebsiella* spp. isolates** | **2737** | **1269** | **437** | **190** | **1119** | **551** | **2004** | **1051** | **471** | **316** | **410** | **160** |
| Blood/CSF isolates | 79 | 50 | 7 | 1 | 73 | 36 | 501 | 231 | 113 | 82 | 145 | 46 |
| ICU isolates | 1163 | 457 | 354 | 142 | 723 | 315 | 1411 | 619 | 401 | 286 | 320 | 133 |
| ***P. aeruginosa* isolates** | **980** | **407** | **204** | **70** | **398** | **191** | **1892** | **1015** | **623** | **435** | **262** | **114** |
| Blood/CSF isolates | 27 | 17 | 2 | 0 | 30 | 14 | 160 | 95 | 67 | 57 | 17 | 5 |
| ICU isolates | 402 | 125 | 157 | 32 | 236 | 93 | 1282 | 662 | 489 | 383 | 361 | 92 |
| ***Acinetobacter* spp. isolates** | **1584** | **877** | **213** | **200** | **638** | **380** | **1636** | **1203** | **512** | **413** | **377** | **258** |
| Blood/CSF isolates | 60 | 28 | 11 | 4 | 24 | 12 | 193 | 145 | 29 | 24 | 33 | 17 |
| ICU isolates | 581 | 362 | 130 | 97 | 432 | 264 | 1282 | 984 | 489 | 395 | 361 | 251 |
| ***S. aureus* isolates** | **2985** | **1207** | **359** | **158** | **1433** | **441** | **1581** | **743** | **276** | **137** | **298** | **134** |
| Blood/CSF isolates | 260 | 178 | 21 | 12 | 253 | 128 | 432 | 201 | 38 | 19 | 134 | 58 |
| ICU isolates | 716 | 230 | 196 | 53 | 474 | 176 | 402 | 194 | 135 | 69 | 161 | 72 |

## Figure S4. Summary of age and gender of patients with hospital acquired (HA) isolates, categorized by main pathogen in two hospitals


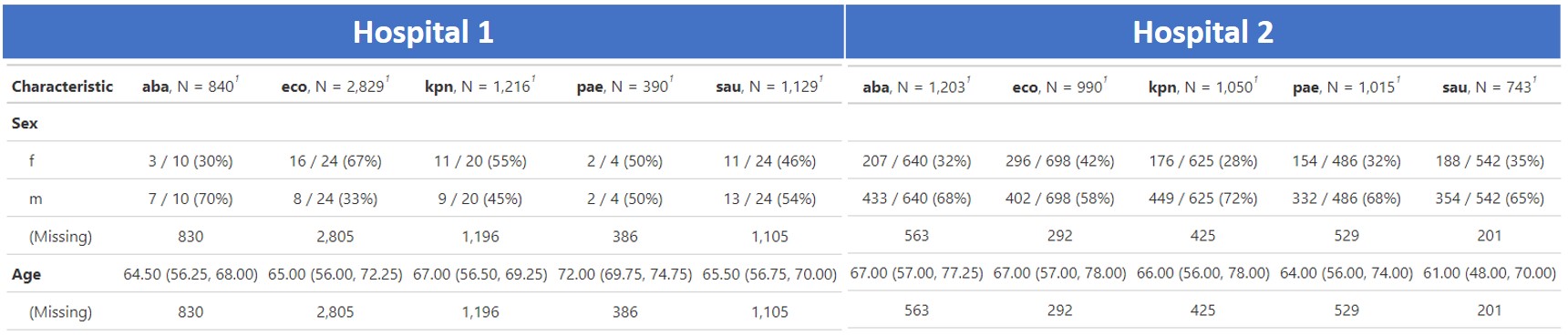


^1^n / N (%); Median (IQR)

Note: aba group contains all *Acinetobacter* spp. isolates; eco group contains all *E. coli* isolates; kpn group contains all *Klebsiella* spp. isolates; pae group contains all *P. aeruginosa* isolates; sau group contains all *S. aureus* isolates.

## Figure S5. Proportion of antibiotic non-susceptibility for main pathogen-drug combinations in intervention and control groups before and after the start of AMS intervention at two hospitals

H1: Hospital 1; H2: Hospital 2; I: Intervention; C: Control; MRSA: Methicillin-resistant Staphylococcus aureus; Those columns with * present results for hospital acquired isolates, the remaining columns are for all isolates in the corresponding group.


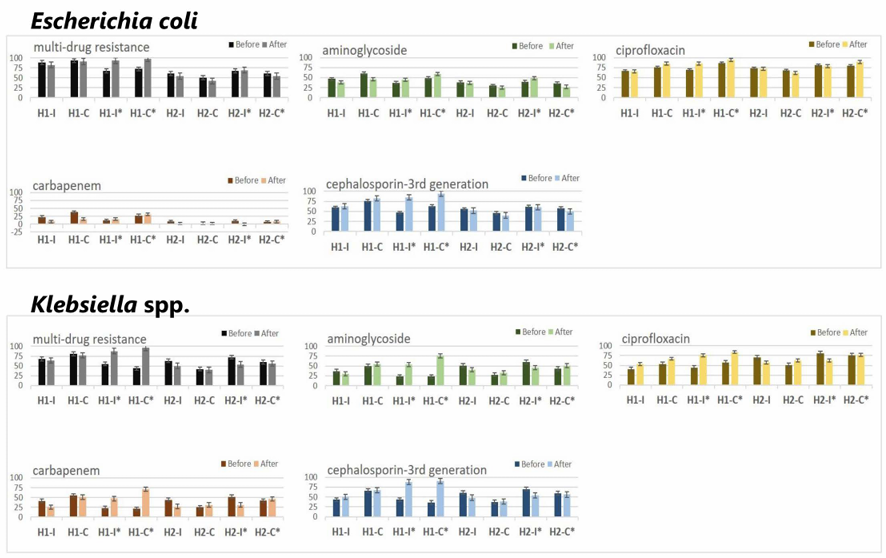

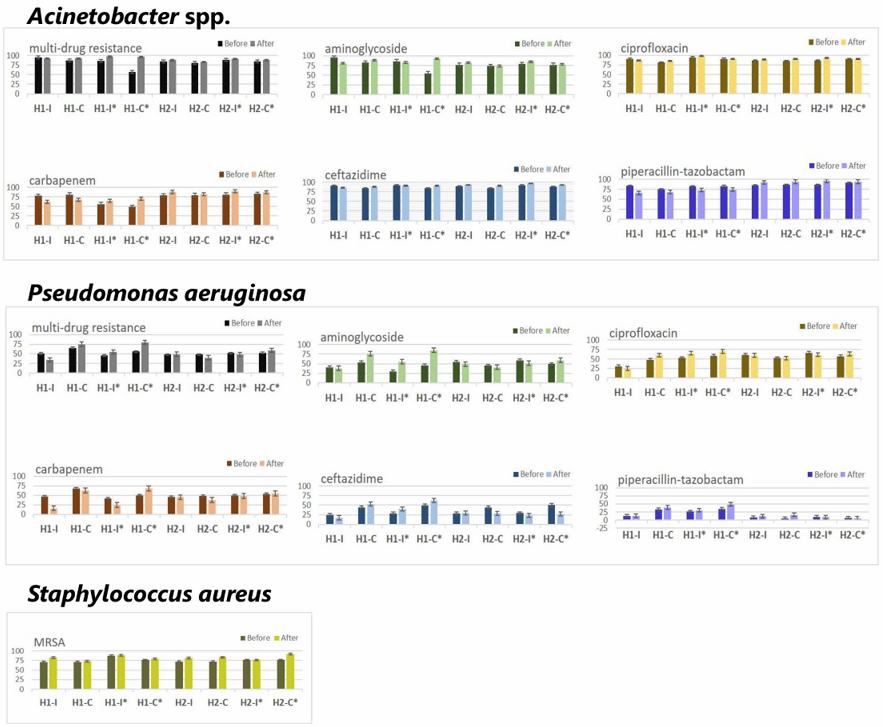


## Table S9. Antibiotic non-susceptibility proportions (all isolates, blood/CSF isolates, ICU isolates) in study wards in Hospital 1 (Jan 2014 - Dec 2021) and Hospital 2 (Dec 2017 - Dec 2021) for common bacteria – *E. coli*

Note: Time periods for antibiotic non-susceptibility data are Jan 2014-Dec 2021 at Hospital 1 (start of AMS intervention was on 01 Jun 2020) and Dec 2017 – Dec 2021 at Hospital 2 (start of AMS intervention was on 29 Jul 2020). Data are presented for all isolates identified in all specimen types, for isolates from blood/CSF, and for isolates from ICU. Non-susceptibility to third-generation cephalosporin: ceftriaxone or ceftazidime; non-susceptibility to aminoglycoside: gentamicin and one of amikacin/ tobramycin; non-susceptibility to carbapenem: one of ertapenem/ imipenem/ meropenem (doripenem was not done); Multidrug resistance is defined as non-susceptible to ≥1 agent in ≥3 antimicrobial categories following Magiorkos et al^8^ in 2012.

| **Drug** | **Period** | **Hospital 1** | | | | | | **Hospital 2** | | | | | |
| --- | --- | --- | --- | --- | --- | --- | --- | --- | --- | --- | --- | --- | --- |
|  |  | **Intervention group** | | | **Control group** | | | **Intervention group** | | | **Control group** | | |
|  |  | **All isolates** | **Blood/ CSF** | **ICU** | **All isolates** | **Blood/ CSF** | **ICU** | **All isolates** | **Blood/ CSF** | **ICU** | **All isolates** | **Blood/ CSF** | **ICU** |
| Gentamicin | Before | 635/1680 (38%) | 9/18  (50%) | 584/1614 (36%) | 795/1505 (53%) | 101/218 (46%) | 433/777 (56%) | 127/276 (46%) | 34/86  (40%) | 75/149  (50%) | 83/203  (41%) | 40/104 (38%) | 33/87  (38%) |
|  | After | 210/588  (36%) | 3/5  (60%) | 181/538  (34%) | 114/240  (48%) | 13/36  (36%) | 47/121  (39%) | 40/86  (47%) | 10/23  (43%) | 28/52  (54%) | 29/71  (41%) | 17/33  (52%) | 10/30  (33%) |
| Tobramycin | Before | 307/926  (33%) | 3/8  (38%) | 283/893  (32%) | 491/1000 (49%) | 61/137  (45%) | 304/539 (56%) | 31/118  (26%) | 6/39  (15%) | 20/67  (30%) | 25/102  (25%) | 11/53  (21%) | 9/41  (22%) |
|  | After | 0/0 | 0/0 | 0/0 | 0/0 | 0/0 | 0/0 | 8/14  (57%) | 3/6  (50%) | 7/8  (88%) | 5/14  (36%) | 4/9  (44%) | 4/8  (50%) |
| Amikacin | Before | 55/1703  (3%) | 0/18  (0%) | 49/1634  (3%) | 130/1528  (9%) | 11/223  (5%) | 64/792  (8%) | 7/274  (3%) | 2/87  (2%) | 4/148  (3%) | 4/203  (2%) | 1/104  (1%) | 3/87  (3%) |
|  | After | 18/653  (3%) | 0/2  (0%) | 14/602  (2%) | 19/276  (7%) | 1/39  (3%) | 5/131  (4%) | 1/84  (1%) | 1/23  (4%) | 1/51  (2%) | 0/73  (0%) | 0/35  (0%) | 0/29  (0%) |
| Neltimicin | Before | 86/1709  (5%) | 1/18  (6%) | 72/1639  (4%) | 169/1533 (11%) | 16/224  (7%) | 80/792  (10%) | 0/0 | 0/0 | 0/0 | 0/0 | 0/0 | 0/0 |
|  | After | 29/677  (4%) | 1/5  (20%) | 22/622  (4%) | 33/282  (12%) | 1/40  (2%) | 9/136  (7%) | 0/0 | 0/0 | 0/0 | 0/0 | 0/0 | 0/0 |
| **Aminoglycoside*** | Before | 812/1714 (47%) | 12/18  (67%) | 753/1644 (46%) | 939/1537 (61%) | 126/224 (56%) | 512/794 (64%) | 133/349 (38%) | 35/148 (24%) | 76/186  (41%) | 89/294  (30%) | 43/190 (23%) | 33/141 (23%) |
|  | After | 260/679  (38%) | 3/5  (60%) | 230/624  (37%) | 130/282  (46%) | 15/40  (38%) | 53/136  (39%) | 45/123  (37%) | 12/58  (21%) | 31/78  (40%) | 30/120  (25%) | 18/76  (24%) | 10/61  (16%) |
| Ticarcillin-clavulanic acid | Before | 356/936  (38%) | 4/8  (50%) | 333/903  (37%) | 572/1006 (57%) | 70/137  (51%) | 352/541 (65%) | 1/6  (17%) | 0/2  (0%) | 1/2  (50%) | 0/4  (0%) | 0/3  (0%) | 0/2  (0%) |
|  | After | 0/0 | 0/0 | 0/0 | 0/0 | 0/0 | 0/0 | 1/3  (33%) | 0/2  (0%) | 1/2  (50%) | 0/1  (0%) | 0/0 | 0/1  (0%) |
| Piperacillin-tazobactam | Before | 220/1714 (13%) | 4/18  (22%) | 198/1644 (12%) | 479/1537 (31%) | 47/224  (21%) | 258/794 (32%) | 34/182  (19%) | 6/53  (11%) | 22/98  (22%) | 15/109  (14%) | 7/58  (12%) | 7/46  (15%) |
|  | After | 57/673  (8%) | 1/5  (20%) | 37/618  (6%) | 62/278  (22%) | 3/40  (8%) | 23/134  (17%) | 7/71  (10%) | 2/21  (10%) | 6/43  (14%) | 6/66  (9%) | 1/29  (3%) | 4/27  (15%) |
| Ertapenem | Before | 94/1704 (6%) | 1/18 (6%) | 79/1634 (5%) | 248/1532 (16%) | 17/224 (8%) | 115/792 (15%) | 19/258 (7%) | 3/84 (4%) | 11/132 (8%) | 6/193 (3%) | 2/97 (2%) | 4/84 (5%) |
|  | After | 23/656  (4%) | 1/5  (20%) | 12/602  (2%) | 34/276  (12%) | 1/39  (3%) | 11/134  (8%) | 2/81  (2%) | 1/22  (5%) | 2/50  (4%) | 2/73  (3%) | 1/38  (3%) | 1/27  (4%) |
| Imipenem | Before | 108/1686  (6%) | 1/18  (6%) | 101/1616  (6%) | 220/1511 (15%) | 16/218  (7%) | 105/784 (13%) | 17/270  (6%) | 2/84  (2%) | 11/146  (8%) | 4/200  (2%) | 2/104  (2%) | 2/86  (2%) |
|  | After | 14/478  (3%) | 0/1  (0%) | 9/443  (2%) | 18/211  (9%) | 0/34  (0%) | 5/96  (5%) | 2/86  (2%) | 1/24  (4%) | 2/52  (4%) | 2/76  (3%) | 1/38  (3%) | 1/30  (3%) |
| Meropenem | Before | 53/1674  (3%) | 1/18  (6%) | 48/1609  (3%) | 158/1507 (10%) | 7/218  (3%) | 60/776  (8%) | 17/273  (6%) | 2/86  (2%) | 11/146  (8%) | 4/202  (2%) | 2/103  (2%) | 2/86  (2%) |
|  | After | 12/575  (2%) | 1/5  (20%) | 6/527  (1%) | 22/237  (9%) | 0/35  (0%) | 6/109  (6%) | 2/86  (2%) | 1/24  (4%) | 2/52  (4%) | 3/76  (4%) | 1/38  (3%) | 2/31  (6%) |
| **Carbapenem**** | Before | 383/1714 (22%) | 4/18  (22%) | 356/1644 (22%) | 578/1537 (38%) | 54/224  (24%) | 297/794 (37%) | 27/349  (8%) | 7/148  (5%) | 14/186  (8%) | 10/294  (3%) | 4/190  (2%) | 5/141  (4%) |
|  | After | 54/679  (8%) | 1/5  (20%) | 38/624  (6%) | 46/282  (16%) | 2/40  (5%) | 19/136  (14%) | 2/123  (2%) | 1/58  (2%) | 2/78  (3%) | 3/120  (2%) | 1/76  (1%) | 2/61  (3%) |
| Cefuroxime | Before | 985/1714 (57%) | 12/18  (67%) | 924/1644 (56%) | 1168/1537 (76%) | 190/224 (85%) | 658/794 (83%) | 70/101  (69%) | 25/37  (68%) | 36/52  (69%) | 56/91  (62%) | 25/45  (56%) | 28/39  (72%) |
|  | After | 387/671  (58%) | 4/5  (80%) | 340/617  (55%) | 226/278  (81%) | 33/40  (82%) | 107/134 (80%) | 19/29  (66%) | 5/7  (71%) | 12/16  (75%) | 11/21  (52%) | 7/12  (58%) | 5/5  (100%) |
| Ceftriaxone | Before | 932/1704 (55%) | 11/18  (61%) | 874/1635 (53%) | 1117/1531 (73%) | 181/224 (81%) | 633/792 (80%) | 22/29  (76%) | 4/6  (67%) | 13/17  (76%) | 12/20  (60%) | 5/9  (56%) | 7/9  (78%) |
|  | After | 360/650  (55%) | 4/5  (80%) | 314/596  (53%) | 211/267  (79%) | 32/39  (82%) | 99/129  (77%) | 2/2  (100%) | 1/1  (100%) | 1/1  (100%) | 0/0 | 0/0 | 0/0 |
| Ceftazidime | Before | 709/1696 (42%) | 7/18  (39%) | 657/1628 (40%) | 960/1526 (63%) | 142/221 (64%) | 549/788 (70%) | 117/276 (42%) | 28/87  (32%) | 69/147  (47%) | 68/200  (34%) | 33/102 (32%) | 28/86  (33%) |
|  | After | 225/664  (34%) | 2/5  (40%) | 191/610  (31%) | 161/277  (58%) | 19/38  (50%) | 71/133  (53%) | 33/83  (40%) | 8/22  (36%) | 24/52  (46%) | 22/76  (29%) | 12/38  (32%) | 9/31  (29%) |
| Cefepime | Before | 553/1713 (32%) | 5/18  (28%) | 506/1643 (31%) | 805/1536 (52%) | 107/224 (48%) | 452/794 (57%) | 73/263  (28%) | 13/84  (15%) | 44/141  (31%) | 40/186  (22%) | 18/91  (20%) | 16/81  (20%) |
|  | After | 233/654  (36%) | 2/3  (67%) | 199/602  (33%) | 161/274  (59%) | 18/39  (46%) | 70/130  (54%) | 26/85  (31%) | 6/24  (25%) | 18/52  (35%) | 15/76  (20%) | 9/38  (24%) | 9/30  (30%) |
| **Third generation Cephalosporin^#^** | Before | 1025/1714 (60%) | 12/18  (67%) | 963/1644 (59%) | 1175/1537 (76%) | 188/224 (84%) | 659/794 (83%) | 196/349 (56%) | 54/148 (36%) | 110/186 (59%) | 135/294 (46%) | 63/190 (33%) | 64/141 (45%) |
|  | After | 429/679  (63%) | 4/5  (80%) | 381/624  (61%) | 232/282  (82%) | 34/40  (85%) | 107/136 (79%) | 64/123  (52%) | 17/58  (29%) | 42/78  (54%) | 48/120  (40%) | 23/76  (30%) | 19/61  (31%) |
| Cefoxitin | Before | 363/1712 (21%) | 5/18  (28%) | 330/1642 (20%) | 640/1537 (42%) | 73/224  (33%) | 342/794 (43%) | 26/67  (39%) | 8/23  (35%) | 11/31  (35%) | 14/57  (25%) | 7/33  (21%) | 5/21  (24%) |
|  | After | 189/625  (30%) | 0/2  (0%) | 165/577  (29%) | 120/263  (46%) | 13/39  (33%) | 58/126  (46%) | 8/25  (32%) | 2/5  (40%) | 6/14  (43%) | 3/17  (18%) | 3/12  (25%) | 2/5  (40%) |
| Cefotetan | Before | 94/928  (10%) | 1/8  (12%) | 88/896  (10%) | 254/1001 (25%) | 20/133  (15%) | 146/536 (27%) | 0/0 | 0/0 | 0/0 | 0/0 | 0/0 | 0/0 |
|  | After | 0/0 | 0/0 | 0/0 | 0/0 | 0/0 | 0/0 | 0/0 | 0/0 | 0/0 | 0/0 | 0/0 | 0/0 |
| Ciprofloxacin | Before | 1130/1691 (67%) | 13/18  (72%) | 1070/1623 (66%) | 1151/1523 (76%) | 181/221 (82%) | 651/787 (83%) | 192/262 (73%) | 56/81  (69%) | 112/142 (79%) | 131/194 (68%) | 61/99  (62%) | 57/83  (69%) |
|  | After | 442/668  (66%) | 5/5  (100%) | 394/613  (64%) | 231/272  (85%) | 34/40  (85%) | 110/134 (82%) | 62/86  (72%) | 15/23  (65%) | 41/53  (77%) | 47/76  (62%) | 18/37  (49%) | 17/30  (57%) |
| Trimethoprim-sulphamethoxazole | Before | 1140/1678 (68%) | 12/18  (67%) | 1088/1610 (68%) | 1076/1516 (71%) | 147/217 (68%) | 559/783 (71%) | 204/270 (76%) | 60/86  (70%) | 108/143 (76%) | 152/200 (76%) | 72/101 (71%) | 64/85  (75%) |
|  | After | 389/627  (62%) | 3/5  (60%) | 345/574  (60%) | 176/253  (70%) | 26/38  (68%) | 81/122  (66%) | 58/81  (72%) | 17/22  (77%) | 37/51  (73%) | 56/70  (80%) | 27/34  (79%) | 21/28  (75%) |
| Aztreonam | Before | 296/935  (32%) | 3/8  (38%) | 272/902  (30%) | 519/1004 (52%) | 74/137  (54%) | 323/539 (60%) | 0/0 | 0/0 | 0/0 | 0/0 | 0/0 | 0/0 |
|  | After | 0/0 | 0/0 | 0/0 | 0/0 | 0/0 | 0/0 | 0/0 | 0/0 | 0/0 | 0/0 | 0/0 | 0/0 |
| Amoxicillin-clavulanic acid | Before | 622/1714 (36%) | 11/18  (61%) | 578/1644 (35%) | 932/1537 (61%) | 127/224 (57%) | 515/794 (65%) | 65/171  (38%) | 16/51  (31%) | 40/92  (43%) | 46/126  (37%) | 23/58  (40%) | 21/55  (38%) |
|  | After | 235/678  (35%) | 1/5  (20%) | 204/623  (33%) | 141/282  (50%) | 13/40  (32%) | 66/136  (49%) | 21/56  (38%) | 5/16  (31%) | 14/37  (38%) | 8/50  (16%) | 4/24  (17%) | 2/20  (10%) |
| Chloramphenicol | Before | 293/926  (32%) | 2/7  (29%) | 283/893  (32%) | 353/998  (35%) | 40/137  (29%) | 208/537 (39%) | 18/72  (25%) | 11/35  (31%) | 10/40  (25%) | 20/83  (24%) | 11/49  (22%) | 7/37  (19%) |
|  | After | 0/0 | 0/0 | 0/0 | 0/0 | 0/0 | 0/0 | 5/11  (45%) | 2/5  (40%) | 4/7  (57%) | 3/11  (27%) | 3/11  (27%) | 2/4  (50%) |
| Fosfomycin | Before | 0/0 | 0/0 | 0/0 | 0/0 | 0/0 | 0/0 | 3/89  (3%) | 0/16  (0%) | 2/43  (5%) | 3/43  (7%) | 0/17  (0%) | 0/16  (0%) |
|  | After | 0/0 | 0/0 | 0/0 | 0/0 | 0/0 | 0/0 | 0/27  (0%) | 0/5  (0%) | 0/15  (0%) | 0/27  (0%) | 0/12  (0%) | 0/13  (0%) |
| Colistin | Before | 22/1687  (1%) | 0/16  (0%) | 22/1621  (1%) | 38/1436  (3%) | 6/217  (3%) | 25/728  (3%) | 0/5  (0%) | 0/1  (0%) | 0/3  (0%) | 0/5  (0%) | 0/1  (0%) | 0/4  (0%) |
|  | After | 6/602  (1%) | 0/2  (0%) | 5/554  (1%) | 3/249  (1%) | 0/34  (0%) | 3/118  (3%) | 0/0 | 0/0 | 0/0 | 0/0 | 0/0 | 0/0 |
| Tetracycline | Before | 1262/1691 (75%) | 14/17  (82%) | 1203/1623 (74%) | 1191/1508 (79%) | 151/219 (69%) | 623/778 (80%) | 66/97  (68%) | 20/34  (59%) | 36/51  (71%) | 57/91  (63%) | 24/45  (53%) | 22/39  (56%) |
|  | After | 453/634  (71%) | 3/3  (100%) | 415/583  (71%) | 195/264  (74%) | 25/39  (64%) | 94/128  (73%) | 9/12  (75%) | 1/2  (50%) | 6/9  (67%) | 3/5  (60%) | 2/4  (50%) | 0/0 |
| **Multidrug resistance^$^** | Before | 1500/1714 (88%) | 17/18 (94%) | 1431/1644 (87%) | 1422/1537 (93%) | 211/224 (94%) | 762/794 (96%) | 214/349 (61%) | 60/148 (41%) | 121/186 (65%) | 148/294 (50%) | 66/190 (35%) | 65/141 (46%) |
|  | After | 555/679  (82%) | 5/5  (100%) | 501/624  (80%) | 256/282  (91%) | 36/40  (90%) | 126/136 (93%) | 66/123  (54%) | 18/58  (31%) | 45/78  (58%) | 50/120  (42%) | 23/76  (30%) | 20/61  (33%) |

## Table S10. Antibiotic non-susceptibility proportions (all isolates, blood/CSF isolates, ICU isolates) in study wards in Hospital 1 (Jan 2014 - Dec 2021) and Hospital 2 (Dec 2017 - Dec 2021) for common bacteria – *Klebsiella* spp.

Note: Time periods for antibiotic non-susceptibility data are Jan 2014-Dec 2021 at Hospital 1 (start of AMS intervention was on 01 Jun 2020) and Dec 2017 – Dec 2021 at Hospital 2 (start of AMS intervention was on 29 Jul 2020). Data are presented for all isolates identified in all specimen types, for isolates from blood/CSF, and for isolates from ICU. Non-susceptibility to third-generation cephalosporin: ceftriaxone or ceftazidime; non-susceptibility to aminoglycoside: gentamicin and one of amikacin/ tobramycin; non-susceptibility to carbapenem: one of ertapenem/ imipenem/ meropenem (doripenem was not done); Multidrug resistance is defined as non-susceptible to ≥1 agent in ≥3 antimicrobial categories following Magiorkos et al^8^ in 2012.

| **Drug** | **Period** | **Hospital 1** | | | | | | **Hospital 2** | | | | | |
| --- | --- | --- | --- | --- | --- | --- | --- | --- | --- | --- | --- | --- | --- |
|  |  | **Intervention group** | | | **Control group** | | | **Intervention group** | | | **Control group** | | |
|  |  | **All isolates** | **Blood/ CSF** | **ICU** | **All isolates** | **Blood/ CSF** | **ICU** | **All isolates** | **Blood/ CSF** | **ICU** | **All isolates** | **Blood/ CSF** | **ICU** |
| Gentamicin | Before | 90/302  (30%) | 3/6  (50%) | 67/262  (26%) | 403/938 (43%) | 22/60 (37%) | 288/611 (47%) | 151/298 (51%) | 27/48 (56%) | 135/250 (54%) | 63/230  (27%) | 8/54  (15%) | 55/184  (30%) |
|  | After | 21/104  (20%) | 0/1  (0%) | 13/69  (19%) | 57/142  (40%) | 2/11  (18%) | 25/90  (28%) | 42/93  (45%) | 4/10  (40%) | 38/82  (46%) | 35/79  (44%) | 6/19  (32%) | 30/60  (50%) |
| Tobramycin | Before | 46/164  (28%) | 1/1 (100%) | 39/154  (25%) | 283/662 (43%) | 10/42 (24%) | 212/443 (48%) | 66/138  (48%) | 9/14  (64%) | 61/113  (54%) | 40/118  (34%) | 2/17  (12%) | 37/99  (37%) |
|  | After | 0/0 | 0/0 | 0/0 | 0/0 | 0/0 | 0/0 | 7/28  (25%) | 0/1  (0%) | 7/25  (28%) | 17/23  (74%) | 3/4  (75%) | 17/22  (77%) |
| Amikacin | Before | 32/308  (10%) | 1/6  (17%) | 20/266  (8%) | 201/952 (21%) | 9/61  (15%) | 146/617 (24%) | 42/299  (14%) | 9/48  (19%) | 37/250  (15%) | 7/228  (3%) | 2/54  (4%) | 6/182  (3%) |
|  | After | 16/127  (13%) | 0/1  (0%) | 11/88  (12%) | 46/154  (30%) | 3/12  (25%) | 23/98  (23%) | 4/94  (4%) | 0/11  (0%) | 3/84  (4%) | 4/77  (5%) | 0/19  (0%) | 3/58  (5%) |
| Neltimicin | Before | 32/308  (10%) | 1/6  (17%) | 19/266  (7%) | 223/955 (23%) | 9/61  (15%) | 161/620 (26%) | 0/0 | 0/0 | 0/0 | 0/0 | 0/0 | 0/0 |
|  | After | 17/129  (13%) | 0/1  (0%) | 10/88  (11%) | 49/162  (30%) | 3/12  (25%) | 23/102  (23%) | 0/0 | 0/0 | 0/0 | 0/0 | 0/0 | 0/0 |
| **Aminoglycoside*** | Before | 113/308 (37%) | 4/6  (67%) | 88/266  (33%) | 475/956 (50%) | 27/61 (44%) | 333/621 (54%) | 180/351 (51%) | 34/86 (40%) | 162/296 (55%) | 81/293  (28%) | 10/102 (10%) | 71/226  (31%) |
|  | After | 39/129  (30%) | 0/1  (0%) | 25/88  (28%) | 90/163  (55%) | 4/12  (33%) | 50/102  (49%) | 49/120  (41%) | 4/27  (15%) | 45/105  (43%) | 37/117  (32%) | 6/43  (14%) | 32/94  (34%) |
| Ticarcillin-clavulanic acid | Before | 65/165  (39%) | 1/1 (100%) | 59/155  (38%) | 351/668 (53%) | 19/42 (45%) | 256/446 (57%) | 5/18  (28%) | 0/1  (0%) | 5/17  (29%) | 3/7  (43%) | 1/5  (20%) | 3/6  (50%) |
|  | After | 0/0 | 0/0 | 0/0 | 0/0 | 0/0 | 0/0 | 5/8  (62%) | 0/1  (0%) | 5/7  (71%) | 5/6  (83%) | 0/0 | 5/6  (83%) |
| Piperacillin-tazobactam | Before | 69/308  (22%) | 1/6  (17%) | 55/266  (21%) | 339/956 (35%) | 13/61 (21%) | 232/621 (37%) | 118/198 (60%) | 25/38 (66%) | 107/171 (63%) | 54/142  (38%) | 7/38  (18%) | 48/111  (43%) |
|  | After | 28/128  (22%) | 0/1  (0%) | 19/87  (22%) | 80/160  (50%) | 4/11  (36%) | 42/99  (42%) | 33/83  (40%) | 1/7  (14%) | 33/76  (43%) | 36/74  (49%) | 6/18  (33%) | 31/57  (54%) |
| Ertapenem | Before | 65/307  (21%) | 2/6  (33%) | 53/265  (20%) | 301/953 (32%) | 12/61 (20%) | 211/618 (34%) | 133/272 (49%) | 26/48 (54%) | 122/224 (54%) | 58/205  (28%) | 6/48  (12%) | 53/163  (33%) |
|  | After | 29/127  (23%) | 0/1  (0%) | 16/87  (18%) | 79/163  (48%) | 4/12  (33%) | 41/102  (40%) | 21/79  (27%) | 2/11  (18%) | 20/68  (29%) | 17/59  (29%) | 4/18  (22%) | 15/43  (35%) |
| Imipenem | Before | 49/304  (16%) | 1/6  (17%) | 39/262  (15%) | 253/940 (27%) | 11/61 (18%) | 170/612 (28%) | 118/295 (40%) | 24/48 (50%) | 107/246 (43%) | 58/229  (25%) | 6/54  (11%) | 54/183  (30%) |
|  | After | 16/103  (16%) | 0/1  (0%) | 11/69  (16%) | 61/126  (48%) | 2/11  (18%) | 30/77  (39%) | 27/97  (28%) | 2/11  (18%) | 26/86  (30%) | 30/80  (38%) | 5/20  (25%) | 26/61  (43%) |
| Meropenem | Before | 46/297  (15%) | 1/5  (20%) | 36/258  (14%) | 216/945 (23%) | 10/61 (16%) | 152/615 (25%) | 123/299 (41%) | 25/49 (51%) | 113/249 (45%) | 60/226  (27%) | 7/54  (13%) | 55/181  (30%) |
|  | After | 20/109  (18%) | 0/1  (0%) | 12/72  (17%) | 70/148  (47%) | 2/10  (20%) | 35/92  (38%) | 29/99  (29%) | 2/11  (18%) | 28/88  (32%) | 32/80  (40%) | 6/20  (30%) | 27/61  (44%) |
| **Carbapenem**** | Before | 125/308 (41%) | 2/6  (33%) | 105/266 (39%) | 524/956 (55%) | 31/61 (51%) | 349/621 (56%) | 156/351 (44%) | 27/86 (31%) | 142/296 (48%) | 73/293  (25%) | 7/102  (7%) | 68/226  (30%) |
|  | After | 32/129  (25%) | 0/1  (0%) | 19/88  (22%) | 83/163  (51%) | 4/12  (33%) | 44/102  (43%) | 32/120  (27%) | 2/27  (7%) | 31/105  (30%) | 36/117  (31%) | 6/43  (14%) | 31/94  (33%) |
| Cefuroxime | Before | 138/307 (45%) | 2/6  (33%) | 109/265 (41%) | 586/956 (61%) | 33/61 (54%) | 396/621 (64%) | 66/106  (62%) | 8/11  (73%) | 59/83  (71%) | 37/90  (41%) | 2/14  (14%) | 32/75  (43%) |
|  | After | 58/129  (45%) | 0/1  (0%) | 37/88  (42%) | 106/160 (66%) | 6/12  (50%) | 63/102  (62%) | 12/33  (36%) | 2/4  (50%) | 7/25  (28%) | 4/16  (25%) | 3/8  (38%) | 3/11  (27%) |
| Ceftriaxone | Before | 115/307 (37%) | 2/6  (33%) | 92/265  (35%) | 518/953 (54%) | 26/61 (43%) | 359/618 (58%) | 20/27  (74%) | 1/2  (50%) | 20/24  (83%) | 7/27  (26%) | 0/3  (0%) | 7/22  (32%) |
|  | After | 46/125  (37%) | 0/1  (0%) | 31/87  (36%) | 96/154  (62%) | 3/12  (25%) | 57/99  (58%) | 3/4  (75%) | 0/0 | 3/4  (75%) | 1/2  (50%) | 0/0 | 1/2  (50%) |
| Ceftazidime | Before | 107/304 (35%) | 1/6  (17%) | 86/263  (33%) | 514/953 (54%) | 24/61 (39%) | 351/619 (57%) | 191/302 (63%) | 37/49 (76%) | 171/252 (68%) | 82/231  (35%) | 11/54  (20%) | 74/185  (40%) |
|  | After | 47/126  (37%) | 0/1  (0%) | 32/86  (37%) | 92/159  (58%) | 3/12  (25%) | 53/100  (53%) | 44/93  (47%) | 3/11  (27%) | 41/83  (49%) | 38/75  (51%) | 7/18  (39%) | 32/58  (55%) |
| Cefepime | Before | 83/308  (27%) | 2/6  (33%) | 66/266  (25%) | 373/956 (39%) | 18/61 (30%) | 250/621 (40%) | 147/285 (52%) | 29/48 (60%) | 134/239 (56%) | 62/210  (30%) | 9/53  (17%) | 57/169  (34%) |
|  | After | 33/126  (26%) | 0/1  (0%) | 22/87  (25%) | 92/155  (59%) | 3/12  (25%) | 54/99  (55%) | 33/98  (34%) | 3/11  (27%) | 30/87  (34%) | 35/78  (45%) | 5/19  (26%) | 31/60  (52%) |
| **Third generation Cephalosporin#** | Before | 132/308 (43%) | 2/6  (33%) | 106/266 (40%) | 628/956 (66%) | 36/61 (59%) | 420/621 (68%) | 213/351 (61%) | 38/86 (44%) | 185/296 (62%) | 107/293 (37%) | 13/102 (13%) | 92/226  (41%) |
|  | After | 65/129  (50%) | 1/1 (100%) | 41/88  (47%) | 109/163 (67%) | 4/12  (33%) | 63/102  (62%) | 58/120  (48%) | 5/27  (19%) | 53/105  (50%) | 44/117  (38%) | 8/43  (19%) | 36/94  (38%) |
| Cefoxitin | Before | 104/306 (34%) | 2/6  (33%) | 84/264  (32%) | 453/956 (47%) | 20/61 (33%) | 284/621 (46%) | 33/80  (41%) | 3/7  (43%) | 27/63  (43%) | 17/50  (34%) | 1/10  (10%) | 15/41  (37%) |
|  | After | 54/124  (44%) | 1/1 (100%) | 33/87  (38%) | 81/145  (56%) | 5/12  (42%) | 42/92  (46%) | 7/31  (23%) | 1/4  (25%) | 5/24  (21%) | 3/16  (19%) | 2/8  (25%) | 3/11  (27%) |
| Cefotetan | Before | 39/163  (24%) | 1/1 (100%) | 36/153  (24%) | 213/663 (32%) | 7/42  (17%) | 153/443 (35%) | 0/0 | 0/0 | 0/0 | 0/0 | 0/0 | 0/0 |
|  | After | 0/0 | 0/0 | 0/0 | 0/0 | 0/0 | 0/0 | 0/0 | 0/0 | 0/0 | 0/0 | 0/0 | 0/0 |
| Ciprofloxacin | Before | 122/303 (40%) | 2/6  (33%) | 96/261  (37%) | 507/954 (53%) | 26/61 (43%) | 350/621 (56%) | 200/287 (70%) | 38/49 (78%) | 178/242 (74%) | 111/217 (51%) | 16/53  (30%) | 98/175  (56%) |
|  | After | 67/126  (53%) | 0/1  (0%) | 45/87  (52%) | 107/160 (67%) | 5/12  (42%) | 63/101  (62%) | 56/99  (57%) | 3/11  (27%) | 54/88  (61%) | 50/80  (62%) | 9/20  (45%) | 41/61  (67%) |
| Trimethoprim-sulphamethoxazole | Before | 140/302 (46%) | 0/6  (0%) | 114/260 (44%) | 519/947 (55%) | 28/60 (47%) | 358/615 (58%) | 166/296 (56%) | 31/49 (63%) | 141/248 (57%) | 97/219  (44%) | 16/54  (30%) | 82/174  (47%) |
|  | After | 46/122  (38%) | 1/1 (100%) | 32/85  (38%) | 77/147  (52%) | 2/10  (20%) | 44/91  (48%) | 50/90  (56%) | 4/9  (44%) | 46/81  (57%) | 45/75  (60%) | 8/18  (44%) | 38/57  (67%) |
| Aztreonam | Before | 49/165  (30%) | 0/1  (0%) | 44/155  (28%) | 283/666 (42%) | 13/42 (31%) | 215/444 (48%) | 0/0 | 0/0 | 0/0 | 0/0 | 0/0 | 0/0 |
|  | After | 0/0 | 0/0 | 0/0 | 0/0 | 0/0 | 0/0 | 0/0 | 0/0 | 0/0 | 0/0 | 0/0 | 0/0 |
| Amoxicillin-clavulanic acid | Before | 131/308 (43%) | 3/6  (50%) | 107/266 (40%) | 574/955 (60%) | 32/61 (52%) | 372/621 (60%) | 117/175 (67%) | 27/36 (75%) | 104/148 (70%) | 56/130  (43%) | 10/37  (27%) | 46/99  (46%) |
|  | After | 56/129  (43%) | 1/1 (100%) | 33/88  (38%) | 99/163  (61%) | 6/12  (50%) | 54/102  (53%) | 31/54  (57%) | 1/7  (14%) | 31/51  (61%) | 20/46  (43%) | 3/11  (27%) | 15/32  (47%) |
| Chloramphenicol | Before | 67/164  (41%) | 0/1  (0%) | 65/154  (42%) | 319/663 (48%) | 16/42 (38%) | 228/441 (52%) | 34/96  (35%) | 3/11  (27%) | 26/76  (34%) | 21/87  (24%) | 5/15  (33%) | 17/71  (24%) |
|  | After | 0/0 | 0/0 | 0/0 | 0/0 | 0/0 | 0/0 | 7/24  (29%) | 1/4  (25%) | 4/18  (22%) | 1/9  (11%) | 1/5  (20%) | 1/7  (14%) |
| Colistin | Before | 12/304  (4%) | 1/6  (17%) | 5/263  (2%) | 32/883  (4%) | 7/58  (12%) | 20/557  (4%) | 0/3  (0%) | 0/0 | 0/2  (0%) | 0/8  (0%) | 0/1  (0%) | 0/8  (0%) |
|  | After | 4/118  (3%) | 0/1  (0%) | 3/81  (4%) | 8/137  (6%) | 0/12  (0%) | 4/91  (4%) | 1/1  (100%) | 0/0 | 1/1  (100%) | 2/3  (67%) | 0/0 | 2/3  (67%) |
| Tetracycline | Before | 139/305 (46%) | 1/6  (17%) | 115/263 (44%) | 495/937 (53%) | 28/61 (46%) | 346/610 (57%) | 38/101  (38%) | 3/10  (30%) | 28/78  (36%) | 31/94  (33%) | 4/15  (27%) | 26/78  (33%) |
|  | After | 50/123  (41%) | 1/1 (100%) | 35/86  (41%) | 72/152  (47%) | 2/12  (17%) | 39/97  (40%) | 7/14  (50%) | 2/4  (50%) | 4/11  (36%) | 1/2  (50%) | 1/2  (50%) | 0/1  (0%) |
| **Multidrug resistance^$^** | Before | 209/308 (68%) | 3/6  (50%) | 173/266 (65%) | 774/956 (81%) | 48/61 (79%) | 510/621 (82%) | 222/351 (63%) | 38/86 (44%) | 193/296  (65%) | 124/293  (42%) | 18/102  (18%) | 108/226  (48%) |
|  | After | 83/129  (64%) | 1/1 (100%) | 54/88  (61%) | 125/163 (77%) | 8/12  (67%) | 74/102  (73%) | 60/120  (50%) | 4/27  (15%) | 55/105  (52%) | 47/117  (40%) | 8/43  (19%) | 39/94  (41%) |

## Table S11. Antibiotic non-susceptibility proportions (all isolates, blood/CSF isolates, ICU isolates) in study wards in Hospital 1 (Jan 2014 - Dec 2021) and Hospital 2 (Dec 2017 - Dec 2021) for common bacteria – *P. aeruginosa*

Note: Time periods for antibiotic non-susceptibility data are Jan 2014-Dec 2021 at Hospital 1 (start of AMS intervention was on 01 Jun 2020) and Dec 2017 – Dec 2021 at Hospital 2 (start of AMS intervention was on 29 Jul 2020). Data are presented for all isolates identified in all specimen types, for isolates from blood/CSF, and for isolates from ICU. Non-susceptibility to aminoglycoside: one of amikacin/tobramycin; Non-susceptibility to carbapenem: one of imipenem/ meropenem (doripenem was not done); Multidrug resistance is defined as non-susceptible to ≥1 agent in ≥3 antimicrobial categories following Magiorkos et al^8^ in 2012.

| **Drug** |  | **Hospital 1** | | | | | | **Hospital 2** | | | | | |
| --- | --- | --- | --- | --- | --- | --- | --- | --- | --- | --- | --- | --- | --- |
|  | **Period** | **Intervention group** | | | **Control group** | | | **Intervention group** | | | **Control group** | | |
|  |  | **All isolates** | **Blood/ CSF** | **ICU** | **All isolates** | **Blood/ CSF** | **ICU** | **All isolates** | **Blood/ CSF** | **ICU** | **All isolates** | **Blood/ CSF** | **ICU** |
| Gentamicin | Before | 37/137 (27%) | 0/0 | 29/113 (26%) | 152/319 (48%) | 10/22 (45%) | 75/186 (40%) | 250/425 (59%) | 19/31 (61%) | 235/350 (67%) | 81/186 (44%) | 2/5  (40%) | 75/162 (46%) |
|  | After | 11/50  (22%) | 1/2 (50%) | 5/35  (14%) | 42/60  (70%) | 8/8 (100%) | 23/38  (61%) | 65/115 (57%) | 3/4  (75%) | 60/97  (62%) | 24/46  (52%) | 1/2  (50%) | 21/36  (58%) |
| Tobramycin | Before | 36/141 (26%) | 0/0 | 29/115 (25%) | 146/323 (45%) | 8/22 (36%) | 70/189 (37%) | 233/401 (58%) | 18/28 (64%) | 219/329 (67%) | 75/180 (42%) | 2/3  (67%) | 69/158 (44%) |
|  | After | 19/60  (32%) | 2/2 (100%) | 10/39  (26%) | 49/71  (69%) | 7/8  (88%) | 24/44  (55%) | 28/63  (44%) | 4/6  (67%) | 26/50  (52%) | 10/19  (53%) | 1/2  (50%) | 9/16  (56%) |
| Amikacin | Before | 23/142 (16%) | 0/0 | 15/116 (13%) | 113/323 (35%) | 4/22 (18%) | 56/192 (29%) | 193/427 (45%) | 16/29 (55%) | 181/351 (52%) | 48/188 (26%) | 2/5  (40%) | 44/163 (27%) |
|  | After | 9/56  (16%) | 0/2  (0%) | 2/35  (6%) | 38/71  (54%) | 2/8  (25%) | 19/44  (43%) | 59/123 (48%) | 5/7  (71%) | 55/104 (53%) | 13/46  (28%) | 0/2  (0%) | 10/35  (29%) |
| Neltimicin | Before | 11/62  (18%) | 0/0 | 9/54  (17%) | 63/190 (33%) | 0/9  (0%) | 35/115 (30%) | 0/0 | 0/0 | 0/0 | 0/0 | 0/0 | 0/0 |
|  | After | 0/0 | 0/0 | 0/0 | 0/0 | 0/0 | 0/0 | 0/0 | 0/0 | 0/0 | 0/0 | 0/0 | 0/0 |
| **Aminoglycoside*** | Before | 59/143 (41%) | 0/0 | 46/117 (39%) | 178/327 (54%) | 12/22 (55%) | 92/192 (48%) | 258/473 (55%) | 19/55 (35%) | 240/392 (61%) | 90/199 (45%) | 2/10  (20%) | 81/170 (48%) |
|  | After | 23/61  (38%) | 2/2 (100%) | 13/40  (32%) | 54/71  (76%) | 8/8 (100%) | 28/44  (64%) | 74/150 (49%) | 5/12 (42%) | 68/128 (53%) | 26/63  (41%) | 1/7  (14%) | 22/50  (44%) |
| Ticarcillin-clavulanic acid | Before | 18/61  (30%) | 0/0 | 13/53  (25%) | 97/188 (52%) | 2/9  (22%) | 52/114 (46%) | 33/137 (24%) | 7/15 (47%) | 33/119 (28%) | 0/44  (0%) | 0/1  (0%) | 0/39  (0%) |
|  | After | 0/0 | 0/0 | 0/0 | 0/0 | 0/0 | 0/0 | 5/17 (29%) | 0/0 | 5/16 (31%) | 0/6 (0%) | 0/0 | 0/6 (0%) |
| Piperacillin-tazobactam | Before | 18/143 (13%) | 0/0 | 13/117 (11%) | 109/327 (33%) | 2/22  (9%) | 50/192 (26%) | 34/420  (8%) | 2/29  (7%) | 26/348  (7%) | 10/185  (5%) | 0/5  (0%) | 9/161  (6%) |
|  | After | 8/60  (13%) | 0/2  (0%) | 2/40  (5%) | 28/71  (39%) | 0/8  (0%) | 13/44  (30%) | 17/136 (12%) | 1/7  (14%) | 14/114 (12%) | 8/50  (16%) | 0/2  (0%) | 6/40  (15%) |
| Imipenem | Before | 47/142 (33%) | 0/0 | 37/116 (32%) | 166/322 (52%) | 11/22 (50%) | 90/189 (48%) | 134/425 (32%) | 10/31 (32%) | 124/349 (36%) | 71/185 (38%) | 2/5  (40%) | 66/161 (41%) |
|  | After | 9/47  (19%) | 2/2 (100%) | 3/28  (11%) | 36/51  (71%) | 7/7 (100%) | 16/28  (57%) | 47/136 (35%) | 3/7  (43%) | 44/114 (39%) | 19/51  (37%) | 1/2  (50%) | 14/40  (35%) |
| Meropenem | Before | 25/140 (18%) | 0/0 | 19/115 (17%) | 139/319 (44%) | 3/19 (16%) | 68/185 (37%) | 207/424 (49%) | 15/31 (48%) | 195/348 (56%) | 88/185 (48%) | 2/5  (40%) | 82/160 (51%) |
|  | After | 8/50  (16%) | 0/2  (0%) | 2/31  (6%) | 36/61  (59%) | 0/8  (0%) | 15/34  (44%) | 65/136 (48%) | 6/7  (86%) | 60/114 (53%) | 21/50  (42%) | 1/2  (50%) | 16/39  (41%) |
| **Carbapenem**** | Before | 67/143 (47%) | 0/0 | 53/117 (45%) | 221/327 (68%) | 15/22 (68%) | 127/192 (66%) | 219/473 (46%) | 16/55 (29%) | 205/392 (52%) | 95/199 (48%) | 2/10  (20%) | 88/170 (52%) |
|  | After | 10/61  (16%) | 2/2 (100%) | 4/40  (10%) | 45/71  (63%) | 7/8  (88%) | 21/44  (48%) | 67/150 (45%) | 6/12 (50%) | 62/128 (48%) | 24/63  (38%) | 1/7  (14%) | 19/50  (38%) |
| Ceftazidime | Before | 33/140 (24%) | 0/0 | 24/114 (21%) | 141/323 (44%) | 2/20 (10%) | 65/188 (35%) | 122/433 (28%) | 12/31 (39%) | 110/357 (31%) | 83/187 (44%) | 1/5  (20%) | 76/162 (47%) |
|  | After | 10/59  (17%) | 0/2  (0%) | 4/39  (10%) | 37/70  (53%) | 0/8  (0%) | 17/43  (40%) | 33/111 (30%) | 2/6  (33%) | 29/92  (32%) | 11/39  (28%) | 1/2  (50%) | 8/32  (25%) |
| Cefepime | Before | 27/143 (19%) | 0/0 | 20/117 (17%) | 138/327 (42%) | 3/22 (14%) | 62/192 (32%) | 99/376 (26%) | 9/28 (32%) | 91/314 (29%) | 67/164 (41%) | 1/5  (20%) | 63/141 (45%) |
|  | After | 11/57  (19%) | 0/2  (0%) | 5/36  (14%) | 38/70  (54%) | 0/8  (0%) | 17/43  (40%) | 31/135 (23%) | 2/7  (29%) | 29/113 (26%) | 16/50  (32%) | 1/2  (50%) | 11/39  (28%) |
| Ciprofloxacin | Before | 42/140 (30%) | 0/0 | 28/115 (24%) | 151/324 (47%) | 3/22 (14%) | 71/191 (37%) | 243/400 (61%) | 18/30 (60%) | 229/333 (69%) | 93/180 (52%) | 2/5  (40%) | 87/157 (55%) |
|  | After | 15/60  (25%) | 0/2  (0%) | 6/40  (15%) | 41/68  (60%) | 0/8  (0%) | 21/43  (49%) | 79/133 (59%) | 6/7  (86%) | 71/111 (64%) | 25/48  (52%) | 1/2  (50%) | 22/38  (58%) |
| Levofloxacin | Before | 42/142 (30%) | 0/0 | 27/116 (23%) | 158/326 (48%) | 3/22 (14%) | 75/192 (39%) | 10/30  (33%) | 0/1  (0%) | 8/23  (35%) | 3/9  (33%) | 0/0 | 2/7  (29%) |
|  | After | 15/58  (26%) | 0/2  (0%) | 6/38  (16%) | 38/64  (59%) | 0/7  (0%) | 20/41  (49%) | 1/1  (100%) | 0/0 | 1/1  (100%) | 3/4  (75%) | 0/0 | 2/3  (67%) |
| Aztreonam | Before | 29/141 (21%) | 0/0 | 20/115 (17%) | 122/325 (38%) | 6/22 (27%) | 57/191 (30%) | 0/0 | 0/0 | 0/0 | 0/0 | 0/0 | 0/0 |
|  | After | 5/20  (25%) | 1/2 (50%) | 3/13  (23%) | 15/27  (56%) | 0/4  (0%) | 4/13  (31%) | 0/0 | 0/0 | 0/0 | 0/0 | 0/0 | 0/0 |
| Colistin | Before | 0/49  (0%) | 0/0 | 0/46  (0%) | 7/158  (4%) | 2/8  (25%) | 3/98  (3%) | 0/0 | 0/0 | 0/0 | 0/0 | 0/0 | 0/0 |
|  | After | 3/42  (7%) | 0/1  (0%) | 2/27  (7%) | 4/61  (7%) | 1/5  (20%) | 3/38  (8%) | 0/0 | 0/0 | 0/0 | 0/0 | 0/0 | 0/0 |
| **Multidrug resistance^$^** | Before | 73/143 (51%) | 0/0 | 58/117 (50%) | 217/327 (66%) | 15/22 (68%) | 120/192 (62%) | 227/473 (48%) | 17/55 (31%) | 213/392 (54%) | 95/199 (48%) | 2/10  (20%) | 87/170 (51%) |
|  | After | 21/61  (34%) | 2/2 (100%) | 10/40  (25%) | 53/71  (75%) | 7/8  (88%) | 27/44  (61%) | 73/150 (49%) | 5/12 (42%) | 66/128 (52%) | 25/63  (40%) | 1/7  (14%) | 20/50  (40%) |

## Table S12. Antibiotic non-susceptibility proportions (all isolates, blood/CSF isolates, ICU isolates) in study wards in Hospital 1 (Jan 2014 - Dec 2021) and Hospital 2 (Dec 2017 - Dec 2021) for common bacteria – *Acinetobacter* spp.

Note: Time periods for antibiotic non-susceptibility data are Jan 2014-Dec 2021 at Hospital 1 (start of AMS intervention was on 01 Jun 2020) and Dec 2017 – Dec 2021 at Hospital 2 (start of AMS intervention was on 29 Jul 2020). Data are presented for all isolates identified in all specimen types, for isolates from blood/CSF, and for isolates from ICU. Non-susceptibility to aminoglycoside: one of amikacin/tobramycin; Non-susceptibility to carbapenem: one of imipenem/ meropenem (doripenem was not done); Multidrug resistance is defined as non-susceptible to ≥1 agent in ≥3 antimicrobial categories following Magiorkos et al^8^ in 2012.

| **Drug** |  | **Hospital 1** | | | | | | **Hospital 2** | | | | | |
| --- | --- | --- | --- | --- | --- | --- | --- | --- | --- | --- | --- | --- | --- |
|  | **Period** | **Intervention group** | | | **Control group** | | | **Intervention group** | | | **Control group** | | |
|  |  | **All isolates** | **Blood/ CSF** | **ICU** | **All isolates** | **Blood/ CSF** | **ICU** | **All isolates** | **Blood/ CSF** | **ICU** | **All isolates** | **Blood/ CSF** | **ICU** |
| Gentamicin | Before | 121/140 (86%) | 4/6 (67%) | 83/96  (86%) | 413/550 (75%) | 7/14 (50%) | 286/366 (78%) | 266/343 (78%) | 12/16 (75%) | 255/325 (78%) | 212/272 (78%) | 9/18  (50%) | 208/262 (79%) |
|  | After | 45/63  (71%) | 2/3 (67%) | 22/31  (71%) | 50/64  (78%) | 2/6  (33%) | 40/48  (83%) | 109/124 (88%) | 2/3  (67%) | 106/120 (88%) | 54/65  (83%) | 2/3  (67%) | 52/62  (84%) |
| Tobramycin | Before | 118/139 (85%) | 5/7 (71%) | 77/94  (82%) | 397/561 (71%) | 5/14 (36%) | 274/374 (73%) | 183/291 (63%) | 5/14 (36%) | 178/278 (64%) | 175/229 (76%) | 7/15  (47%) | 172/219 (79%) |
|  | After | 56/72  (78%) | 2/4 (50%) | 26/34  (76%) | 56/75  (75%) | 3/10 (30%) | 43/56  (77%) | 46/53  (87%) | 0/0 | 44/50  (88%) | 35/40  (88%) | 1/2  (50%) | 34/37  (92%) |
| Amikacin | Before | 94/141 (67%) | 5/7 (71%) | 63/96  (66%) | 344/558 (62%) | 5/14 (36%) | 243/372 (65%) | 143/234 (61%) | 6/13 (46%) | 140/224 (62%) | 129/176 (73%) | 6/14  (43%) | 127/167 (76%) |
|  | After | 42/71  (59%) | 2/4 (50%) | 23/33  (70%) | 48/68  (71%) | 5/9  (56%) | 37/52  (71%) | 34/43  (79%) | 0/1  (0%) | 32/40  (80%) | 15/21  (71%) | 0/2  (0%) | 15/19  (79%) |
| Neltimicin | Before | 30/41  (73%) | 2/2 (100%) | 24/35  (69%) | 160/294 (54%) | 1/3  (33%) | 124/212 (58%) | 0/0 | 0/0 | 0/0 | 0/0 | 0/0 | 0/0 |
|  | After | 0/0 | 0/0 | 0/0 | 0/0 | 0/0 | 0/0 | 0/0 | 0/0 | 0/0 | 0/0 | 0/0 | 0/0 |
| **Aminoglycoside*** | Before | 135/141 (96%) | 7/7 (100%) | 91/96  (95%) | 467/563 (83%) | 8/14 (57%) | 321/376 (85%) | 290/376 (77%) | 13/25 (52%) | 279/357 (78%) | 223/300 (74%) | 10/28 (36%) | 219/289 (76%) |
|  | After | 58/72  (81%) | 3/4 (75%) | 27/34  (79%) | 67/75  (89%) | 6/10 (60%) | 49/56  (88%) | 112/136 (82%) | 2/4  (50%) | 109/132 (83%) | 57/77  (74%) | 2/5  (40%) | 55/72  (76%) |
| Ticarcillin-clavulanic acid | Before | 66/70  (94%) | 4/4 (100%) | 54/58  (93%) | 304/389 (78%) | 3/7  (43%) | 231/271 (85%) | 54/62  (87%) | 1/1 (100%) | 51/59  (86%) | 36/41  (88%) | 1/2  (50%) | 36/40  (90%) |
|  | After | 0/0 | 0/0 | 0/0 | 0/0 | 0/0 | 0/0 | 9/10  (90%) | 0/0 | 9/10  (90%) | 11/12  (92%) | 0/0 | 10/11  (91%) |
| Piperacillin-tazobactam | Before | 118/141 (84%) | 3/7 (43%) | 83/96  (86%) | 425/563 (75%) | 6/14 (43%) | 309/376 (82%) | 259/306 (85%) | 10/15 (67%) | 248/292 (85%) | 202/234 (86%) | 6/14  (43%) | 199/228 (87%) |
|  | After | 47/71  (66%) | 1/4 (25%) | 24/33  (73%) | 50/74  (68%) | 4/10 (40%) | 37/55  (67%) | 113/123 (92%) | 2/3  (67%) | 112/121 (93%) | 62/66  (94%) | 2/3  (67%) | 60/63  (95%) |
| Imipenem | Before | 105/141 (74%) | 5/7 (71%) | 71/96  (74%) | 392/559 (70%) | 7/14 (50%) | 283/374 (76%) | 267/341 (78%) | 9/15 (60%) | 254/323 (79%) | 227/271 (84%) | 9/18  (50%) | 221/262 (84%) |
|  | After | 40/59  (68%) | 0/3  (0%) | 18/27  (67%) | 39/50  (78%) | 6/7  (86%) | 32/38  (84%) | 117/128 (91%) | 2/3  (67%) | 115/124 (93%) | 62/69  (90%) | 2/4  (50%) | 60/65  (92%) |
| Meropenem | Before | 101/137 (74%) | 3/7 (43%) | 72/93  (77%) | 419/559 (75%) | 8/13 (62%) | 298/374 (80%) | 261/334 (78%) | 9/16 (56%) | 247/316 (78%) | 233/274 (85%) | 10/19 (53%) | 228/265 (86%) |
|  | After | 43/65  (66%) | 0/3  (0%) | 19/30  (63%) | 43/62  (69%) | 4/6  (67%) | 34/45  (76%) | 118/128 (92%) | 2/3  (67%) | 116/124 (94%) | 62/69  (90%) | 2/4  (50%) | 60/65  (92%) |
| **Carbapenem**** | Before | 110/141 (78%) | 5/7 (71%) | 76/96  (79%) | 457/563 (81%) | 8/14 (57%) | 323/376 (86%) | 296/376 (79%) | 10/25 (40%) | 282/357 (79%) | 239/300 (80%) | 11/28 (39%) | 233/289 (81%) |
|  | After | 45/72  (62%) | 0/4  (0%) | 20/34  (59%) | 50/75  (67%) | 6/10 (60%) | 38/56  (68%) | 119/136 (88%) | 2/4  (50%) | 117/132 (89%) | 63/77  (82%) | 2/5  (40%) | 61/72  (85%) |
| Ceftriaxone | Before | 131/141 (93%) | 5/7 (71%) | 89/96  (93%) | 484/560 (86%) | 7/14 (50%) | 332/373 (89%) | 47/54  (87%) | 2/3  (67%) | 46/53  (87%) | 35/40  (88%) | 2/2  (100%) | 33/37  (89%) |
|  | After | 64/72  (89%) | 2/4 (50%) | 30/34  (88%) | 61/68  (90%) | 8/9  (89%) | 48/52  (92%) | 31/33  (94%) | 0/1  (0%) | 30/32  (94%) | 13/14  (93%) | 0/0 | 13/13 (100%) |
| Ceftazidime | Before | 127/139 (91%) | 4/7 (57%) | 87/95  (92%) | 470/562 (84%) | 8/14 (57%) | 328/376 (87%) | 313/347 (90%) | 13/16 (81%) | 300/329 (91%) | 234/274 (85%) | 11/18 (61%) | 228/263 (87%) |
|  | After | 59/69  (86%) | 2/4 (50%) | 28/34  (82%) | 65/74  (88%) | 8/10 (80%) | 49/55  (89%) | 109/117 (93%) | 2/3  (67%) | 108/115 (94%) | 61/67  (91%) | 2/4  (50%) | 59/63  (94%) |
| Cefepime | Before | 126/141 (89%) | 5/7 (71%) | 85/96  (89%) | 451/563 (80%) | 7/14 (50%) | 316/376 (84%) | 279/316 (88%) | 12/15 (80%) | 267/302 (88%) | 219/253 (87%) | 10/16 (62%) | 216/246 (88%) |
|  | After | 60/72  (83%) | 2/4 (50%) | 27/34  (79%) | 59/69  (86%) | 8/9  (89%) | 46/53  (87%) | 116/127 (91%) | 2/3  (67%) | 114/123 (93%) | 63/69  (91%) | 2/4  (50%) | 61/65  (94%) |
| Ciprofloxacin | Before | 127/140 (91%) | 3/7 (43%) | 87/96  (91%) | 455/559 (81%) | 7/14 (50%) | 314/373 (84%) | 287/335 (86%) | 10/16 (62%) | 275/320 (86%) | 232/272 (85%) | 11/18 (61%) | 226/261 (87%) |
|  | After | 61/70  (87%) | 1/4 (25%) | 27/34  (79%) | 61/72  (85%) | 4/10 (40%) | 47/55  (85%) | 114/128 (89%) | 2/3  (67%) | 112/124 (90%) | 62/69  (90%) | 2/4  (50%) | 60/65  (92%) |
| Levofloxacin | Before | 121/141 (86%) | 2/7 (29%) | 84/96  (88%) | 442/563 (79%) | 5/14 (36%) | 306/376 (81%) | 158/216 (73%) | 6/11 (55%) | 150/205 (73%) | 138/169 (82%) | 4/10  (40%) | 138/165 (84%) |
|  | After | 47/62  (76%) | 1/4 (25%) | 24/32  (75%) | 47/64  (73%) | 4/10 (40%) | 37/48  (77%) | 32/36  (89%) | 0/0 | 31/35  (89%) | 27/29  (93%) | 0/0 | 26/28  (93%) |
| Trimethoprim-sulphamethoxazole | Before | 79/140 (56%) | 1/7 (14%) | 52/95  (55%) | 342/559 (61%) | 6/14 (43%) | 226/373 (61%) | 167/338 (49%) | 5/12 (42%) | 157/320 (49%) | 135/264 (51%) | 8/19  (42%) | 134/255 (53%) |
|  | After | 33/65  (51%) | 1/3 (33%) | 15/32  (47%) | 41/67  (61%) | 5/10 (50%) | 31/50  (62%) | 87/121 (72%) | 0/3  (0%) | 85/117 (73%) | 53/66  (80%) | 2/4  (50%) | 51/62  (82%) |
| Aztreonam | Before | 64/69  (93%) | 3/4 (75%) | 53/57  (93%) | 351/389 (90%) | 6/7  (86%) | 262/271 (97%) | 0/0 | 0/0 | 0/0 | 0/0 | 0/0 | 0/0 |
|  | After | 0/0 | 0/0 | 0/0 | 0/0 | 0/0 | 0/0 | 0/0 | 0/0 | 0/0 | 0/0 | 0/0 | 0/0 |
| Colistin | Before | 3/45  (7%) | 2/2 (100%) | 0/36  (0%) | 3/295  (1%) | 0/3  (0%) | 0/213  (0%) | 0/2  (0%) | 0/0 | 0/1  (0%) | 2/48  (4%) | 0/2  (0%) | 2/48  (4%) |
|  | After | 5/62  (8%) | 0/3  (0%) | 3/27  (11%) | 0/57  (0%) | 0/8  (0%) | 0/44  (0%) | 0/2  (0%) | 0/0 | 0/2  (0%) | 0/1  (0%) | 0/0 | 0/1  (0%) |
| Tetracycline | Before | 90/140 (64%) | 3/7 (43%) | 60/95  (63%) | 309/550 (56%) | 8/14 (57%) | 201/369 (54%) | 24/48  (50%) | 1/2  (50%) | 20/43  (47%) | 27/51  (53%) | 3/6  (50%) | 25/45  (56%) |
|  | After | 35/65  (54%) | 1/4 (25%) | 12/31  (39%) | 35/65  (54%) | 2/8  (25%) | 28/50  (56%) | 1/3  (33%) | 0/0 | 1/2  (50%) | 0/2  (0%) | 0/1  (0%) | 0/1  (0%) |
| **Multidrug resistance^$^** | Before | 134/141 (95%) | 7/7 (100%) | 91/96  (95%) | 492/563 (87%) | 10/14 (71%) | 340/376 (90%) | 320/376 (85%) | 13/25 (52%) | 305/357 (85%) | 242/300 (81%) | 12/28 (43%) | 236/289 (82%) |
|  | After | 66/72  (92%) | 2/4 (50%) | 30/34  (88%) | 69/75  (92%) | 9/10 (90%) | 51/56  (91%) | 120/136 (88%) | 2/4  (50%) | 117/132 (89%) | 64/77  (83%) | 2/5  (40%) | 62/72  (86%) |

## Table S13. Antibiotic non-susceptibility proportions (all isolates, blood/CSF isolates, ICU isolates) in study wards in Hospital 1 (Jan 2014 - Dec 2021) and Hospital 2 (Dec 2017 - Dec 2021) for common bacteria *– S. aureus*

Note: Time periods for antibiotic non-susceptibility data are Jan 2014-Dec 2021 at Hospital 1 (start of AMS intervention was on 01 Jun 2020) and Dec 2017 – Dec 2021 at Hospital 2 (start of AMS intervention was on 29 Jul 2020). Data are presented for all isolates identified in all specimen types, for isolates from blood/CSF, and for isolates from ICU; Multidrug resistance is defined as non-susceptible to ≥1 agent in ≥3 antimicrobial categories following Magiorkos et al^8^ in 2012.

|  | **Period** | **Hospital 1** | | | | | | **Hospital 2** | | | | | |
| --- | --- | --- | --- | --- | --- | --- | --- | --- | --- | --- | --- | --- | --- |
|  |  | **Intervention group** | | | **Control group** | | | **Intervention group** | | | **Control group** | | |
|  |  | **All isolates** | **Blood/ CSF** | **ICU** | **All isolates** | **Blood/ CSF** | **ICU** | **All isolates** | **Blood/ CSF** | **ICU** | **All isolates** | **Blood/ CSF** | **ICU** |
| Multidrug resistance (MRSA) | Before | 165/232 (71%) | 8/11 (73%) | 107/159 (67%) | 830/1173 (71%) | 114/160 (71%) | 268/342 (78%) | 122/169 (72%) | 12/17 (71%) | 58/86 (67%) | 116/162 (72%) | 32/50 (64%) | 74/99 (75%) |
|  | After | 94/114 (82%) | 4/9 (44%) | 24/31 (77%) | 175/239 (73%) | 60/85 (71%) | 94/123 (76%) | 39/48 (81%) | 3/4 (75%) | 11/17 (65%) | 30/36 (83%) | 9/11 (82%) | 12/15 (80%) |

## Table S14. Antibiotic non-susceptibility for main pathogen-drug pairs in intervention and control groups before and after the start of AMS intervention at two hospitals: all isolates versus hospital acquired isolates

**Note.** Time periods for antibiotic non-susceptibility data are Jan 2014-Dec 2021 at Hospital 1 (start of AMS intervention was on 01 Jun 2020) and Dec 2017 – Dec 2021 at Hospital 2 (start of AMS intervention was on 29 Jul 2020). Multidrug resistance is defined as non-susceptible to ≥1 agent in ≥3 antimicrobial categories following Magiorkos et al^6^ in 2012. Non-susceptibility to specific antibiotic subgroups: *Escherichia* *coli* and *Klebsiella* spp.: third-generation cephalosporin (ceftriaxone or ceftazidime), aminoglycoside (gentamicin and one of amikacin/ tobramycin), fluoroquinolone (ciprofloxacin), carbapenem (one of ertapenem/ imipenem/ meropenem/ doripenem); *Pseudomonas aeruginosa* and *Acinetobacter* spp.: third-generation cephalosporin (ceftazidime), aminoglycoside (one of amikacin/tobramycin), fluoroquinolone (ciprofloxacin), carbapenem (one of imipenem/ meropenem/ doripenem), piperacillin-tazobactam, aztreonam, colistin; *Staphylococcus aureus*: MRSA (oxacillin or cefoxitin).

| **Pathogen-Drug** | **Time period** | **Hospital 1** | | | | **Hospital 2** | | | |
| --- | --- | --- | --- | --- | --- | --- | --- | --- | --- |
|  |  | **Intervention** | | **Control** | | **Intervention** | | **Control** | |
|  |  | **All isolates** | **HA isolates** | **All isolates** | **HA isolates** | **All isolates** | **HA isolates** | **All isolates** | **HA isolates** |
| ***Escherichia coli*** |  |  |  |  |  |  |  |  |  |
| Aminoglycoside | Before | 812/1714  (47%) | 242/657 (37%) | 939/1537  (61%) | 395/806 (49%) | 133/349 (38%) | 57/144  (40%) | 89/294  (30%) | 27/74  (36%) |
|  | After | 260/679  (38%) | 65/145  (45%) | 130/282  (46%) | 104/176 (59%) | 45/123  (37%) | 22/45  (49%) | 30/120  (25%) | 7/26  (27%) |
| Ciprofloxacin | Before | 1130/1691 (67%) | 335/476 (70%) | 1151/1523 (76%) | 515/590 (87%) | 192/262 (73%) | 91/113  (81%) | 131/194 (68%) | 44/55  (80%) |
|  | After | 442/668  (66%) | 122/144 (85%) | 231/272  (85%) | 157/167 (94%) | 62/86  (72%) | 27/34  (79%) | 47/76  (62%) | 16/18  (89%) |
| Carbapenem | Before | 383/1714  (22%) | 78/657  (12%) | 578/1537  (38%) | 216/806 (27%) | 27/349  (8%) | 14/144  (10%) | 10/294  (3%) | 5/74  (7%) |
|  | After | 54/679  (8%) | 23/145  (16%) | 46/282  (16%) | 54/176  (31%) | 2/123  (2%) | 0/45  (0%) | 3/120  (2%) | 2/26  (8%) |
| Third-generation cephalosporin | Before | 1025/1714 (60%) | 311/657 (47%) | 1175/1537 (76%) | 508/806 (63%) | 196/349 (56%) | 89/144  (62%) | 135/294 (46%) | 43/74  (58%) |
|  | After | 429/679  (63%) | 123/145 (85%) | 232/282  (82%) | 166/176 (94%) | 64/123  (52%) | 27/45  (60%) | 48/120  (40%) | 13/26  (50%) |
| MDR | Before | 1500/1714 (88%) | 440/657 (67%) | 1422/1537 (93%) | 577/806 (72%) | 214/349 (61%) | 96/144  (67%) | 148/294 (50%) | 45/74  (61%) |
|  | After | 555/679  (82%) | 135/145 (93%) | 256/282  (91%) | 172/176 (98%) | 66/123  (54%) | 31/45  (69%) | 50/120  (42%) | 14/26  (54%) |
| ***Klebsiella spp.*** |  |  |  |  |  |  |  |  |  |
| Aminoglycoside | Before | 113/308  (37%) | 33/141  (23%) | 475/956  (50%) | 106/455 (23%) | 180/351 (51%) | 150/249 (60%) | 81/293  (28%) | 51/119  (43%) |
|  | After | 39/129  (30%) | 26/49  (53%) | 90/163  (55%) | 73/96  (76%) | 49/120  (41%) | 31/67  (46%) | 37/117  (32%) | 21/41  (51%) |
| Ciprofloxacin | Before | 122/303  (40%) | 46/105  (44%) | 507/954  (53%) | 154/268 (57%) | 200/287 (70%) | 166/205 (81%) | 111/217 (51%) | 69/91  (76%) |
|  | After | 67/126  (53%) | 34/45  (76%) | 107/160  (67%) | 81/96  (84%) | 56/99  (57%) | 35/56  (62%) | 50/80  (62%) | 23/30  (77%) |
| Carbapenem | Before | 125/308  (41%) | 32/141  (23%) | 524/956  (55%) | 95/455  (21%) | 156/351 (44%) | 130/249 (52%) | 73/293  (25%) | 50/119  (42%) |
|  | After | 32/129  (25%) | 23/49  (47%) | 83/163  (51%) | 68/96  (71%) | 32/120  (27%) | 21/67  (31%) | 36/117  (31%) | 19/41  (46%) |
| Third-generation cephalosporin | Before | 132/308  (43%) | 60/141  (43%) | 628/956  (66%) | 164/455 (36%) | 213/351 (61%) | 174/249 (70%) | 107/293 (37%) | 70/119  (59%) |
|  | After | 65/129  (50%) | 43/49  (88%) | 109/163  (67%) | 86/96  (90%) | 58/120  (48%) | 36/67  (54%) | 44/117  (38%) | 23/41  (56%) |
| MDR | Before | 209/308  (68%) | 77/141  (55%) | 774/956  (81%) | 201/455 (44%) | 222/351 (63%) | 179/249 (72%) | 124/293 (42%) | 71/119  (60%) |
|  | After | 83/129  (64%) | 43/49  (88%) | 125/163  (77%) | 92/96  (96%) | 60/120  (50%) | 36/67  (54%) | 47/117  (40%) | 23/41  (56%) |
| ***Pseudomonas aeruginosa*** |  |  |  |  |  |  |  |  |  |
| Aminoglycoside | Before | 59/143  (41%) | 15/50  (30%) | 178/327  (54%) | 69/150  (46%) | 258/473 (55%) | 206/348 (59%) | 90/199  (45%) | 45/92  (49%) |
|  | After | 23/61  (38%) | 11/20  (55%) | 54/71  (76%) | 35/41  (85%) | 74/150  (49%) | 44/87  (51%) | 26/63  (41%) | 13/22  (59%) |
| Ciprofloxacin | Before | 42/140  (30%) | 22/42  (52%) | 151/324  (47%) | 63/109  (58%) | 243/400 (61%) | 193/293 (66%) | 93/180  (52%) | 47/83  (57%) |
|  | After | 15/60  (25%) | 13/20  (65%) | 41/68  (60%) | 28/40  (70%) | 79/133  (59%) | 46/75  (61%) | 25/48  (52%) | 12/19  (63%) |
| Carbapenem | Before | 67/143  (47%) | 21/50  (42%) | 221/327  (68%) | 74/150  (49%) | 219/473 (46%) | 174/348 (50%) | 95/199  (48%) | 49/92  (53%) |
|  | After | 10/61  (16%) | 5/20  (25%) | 45/71  (63%) | 28/41  (68%) | 67/150  (45%) | 42/87  (48%) | 24/63  (38%) | 12/22  (55%) |
| Ceftazidime | Before | 33/140  (24%) | 12/43  (28%) | 141/323  (44%) | 53/108  (49%) | 122/433 (28%) | 91/316  (29%) | 83/187  (44%) | 44/86  (51%) |
|  | After | 10/59  (17%) | 8/20  (40%) | 37/70  (53%) | 25/40  (62%) | 33/111  (30%) | 14/61  (23%) | 11/39  (28%) | 4/15  (27%) |
| Piperacillin-tazobactam | Before | 18/143  (13%) | 11/43  (26%) | 109/327  (33%) | 38/110  (35%) | 34/420  (8%) | 30/309  (10%) | 10/185  (5%) | 6/83  (7%) |
|  | After | 8/60  (13%) | 6/20  (30%) | 28/71  (39%) | 20/41  (49%) | 17/136  (12%) | 7/77  (9%) | 8/50  (16%) | 1/20  (5%) |
| Aztreonam | Before | 29/141  (21%) | 12/43  (28%) | 122/325  (38%) | 46/105  (44%) | 0/0 | 0/0 | 0/0 | 0/0 |
|  | After | 5/20  (25%) | 1/8  (12%) | 15/27  (56%) | 13/21  (62%) | 0/0 | 0/0 | 0/0 | 0/0 |
| Colistin | Before | 0/49  (0%) | 0/1  (0%) | 7/158  (4%) | 1/8  (12%) | 0/0 | 0/0 | 0/0 | 0/0 |
|  | After | 3/42  (7%) | 0/13  (0%) | 4/61  (7%) | 3/29  (10%) | 0/0 | 0/0 | 0/0 | 0/0 |
| MDR | Before | 73/143  (51%) | 23/50  (46%) | 217/327  (66%) | 84/150  (56%) | 227/473 (48%) | 181/348 (52%) | 95/199  (48%) | 49/92  (53%) |
|  | After | 21/61  (34%) | 11/20  (55%) | 53/71  (75%) | 33/41  (80%) | 73/150  (49%) | 42/87  (48%) | 25/63  (40%) | 13/22  (59%) |
| ***Acinetobacter spp.*** |  |  |  |  |  |  |  |  |  |
| Aminoglycoside | Before | 135/141  (96%) | 120/140 (86%) | 467/563  (83%) | 172/310 (55%) | 290/376 (77%) | 244/310 (79%) | 223/300 (74%) | 153/198 (77%) |
|  | After | 58/72  (81%) | 50/60  (83%) | 67/75  (89%) | 65/70  (93%) | 112/136 (82%) | 88/103  (85%) | 57/77  (74%) | 47/60  (78%) |
| Ciprofloxacin | Before | 127/140  (91%) | 118/124 (95%) | 455/559  (81%) | 162/179 (91%) | 287/335 (86%) | 245/282 (87%) | 232/272 (85%) | 160/177 (90%) |
|  | After | 61/70  (87%) | 57/58  (98%) | 61/72  (85%) | 60/67  (90%) | 114/128 (89%) | 90/97  (93%) | 62/69  (90%) | 51/56  (91%) |
| Carbapenem | Before | 110/141  (78%) | 78/140  (56%) | 457/563  (81%) | 153/310 (49%) | 296/376 (79%) | 251/310 (81%) | 239/300 (80%) | 165/198 (83%) |
|  | After | 45/72  (62%) | 39/60  (65%) | 50/75  (67%) | 49/70  (70%) | 119/136 (88%) | 93/103  (90%) | 63/77  (82%) | 52/60  (87%) |
| Ceftazidime | Before | 127/139  (91%) | 112/122 (92%) | 470/562  (84%) | 153/183 (84%) | 313/347 (90%) | 267/289 (92%) | 234/274 (85%) | 160/179 (89%) |
|  | After | 59/69  (86%) | 53/58  (91%) | 65/74  (88%) | 63/69  (91%) | 109/117 (93%) | 84/87  (97%) | 61/67  (91%) | 50/54  (93%) |
| Piperacillin-tazobactam | Before | 118/141  (84%) | 102/124 (82%) | 425/563  (75%) | 151/183 (83%) | 259/306 (85%) | 219/254 (86%) | 202/234 (86%) | 142/156 (91%) |
|  | After | 47/71  (66%) | 44/60  (73%) | 50/74  (68%) | 52/70  (74%) | 113/123 (92%) | 89/93  (96%) | 62/66  (94%) | 51/54  (94%) |
| Aztreonam | Before | 64/69  (93%) | 18/22  (82%) | 351/389  (90%) | 67/72  (93%) | 0/0 | 0/0 | 0/0 | 0/0 |
|  | After | 0/0 | 0/0 | 0/0 | 0/0 | 0/0 | 0/0 | 0/0 | 0/0 |
| Colistin | Before | 3/45  (7%) | 2/5  (40%) | 3/295  (1%) | 0/4  (0%) | 0/2  (0%) | 0/2  (0%) | 2/48  (4%) | 1/30  (3%) |
|  | After | 5/62  (8%) | 3/51  (6%) | 0/57  (0%) | 0/55  (0%) | 0/2  (0%) | 0/0 | 0/1  (0%) | 0/0 |
| MDR | Before | 134/141  (95%) | 120/140 (86%) | 492/563  (87%) | 178/310 (57%) | 320/376 (85%) | 272/310 (88%) | 242/300 (81%) | 166/198 (84%) |
|  | After | 66/72  (92%) | 58/60  (97%) | 69/75  (92%) | 67/70  (96%) | 120/136 (88%) | 94/103  (91%) | 64/77  (83%) | 53/60  (88%) |
| ***Staphylococcus aureus*** |  |  |  |  |  |  |  |  |  |
| MDR (MRSA) | Before | 165/232  (71%) | 74/85  (87%) | 830/1173  (71%) | 210/278 (76%) | 122/169 (72%) | 71/93  (76%) | 116/162 (72%) | 60/79  (76%) |
|  | After | 94/114  (82%) | 50/57  (88%) | 175/239  (73%) | 88/112  (79%) | 39/48  (81%) | 19/25  (76%) | 30/36  (83%) | 11/12  (92%) |

# **D. Model results**

In each hospital, 46 subgroup analyses were conducted. For each subgroup, three Interrupted Time Series (ITS) models were fitted: one for the intervention group, one for the control group, and one for the combined control and intervention (CITS). To account for multiple comparisons, a Bonferroni correction was applied to adjust the significance threshold for residual testing. Given a total of 138 tests per hospital (46 subgroups × 3 models), the adjusted significance level was set at 0.00036 (0.05/138)^10^.

## Figure S6. Results of ITS and CITS models for antibiotic use and antibiotic non-susceptibility among the hospital-acquired common pathogens identified from routine microbiology in the intervention group at two hospitals.

Note: ^1^Odds ratio between non-susceptibility vs susceptibility. Bold values: statistically significant estimate; Green shades: decreasing trends; Red shades: increasing trends; Dark shades: consistent significant results for ITS/CITS models. Pip-tazobactam: Piperacillin-tazobactam.

***Figure S6.1. Antibiotic use***

*
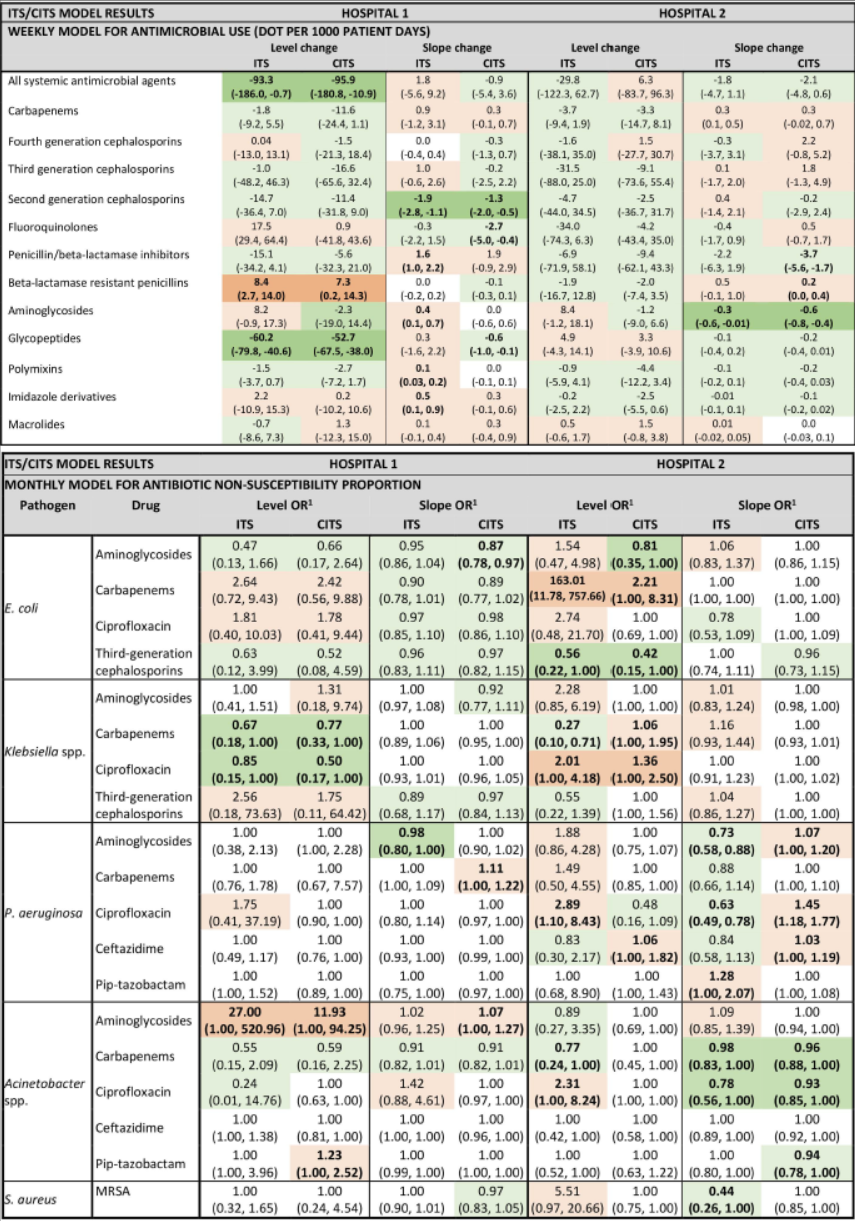
*

***Figure S6.2. Antibiotic non-susceptibility***


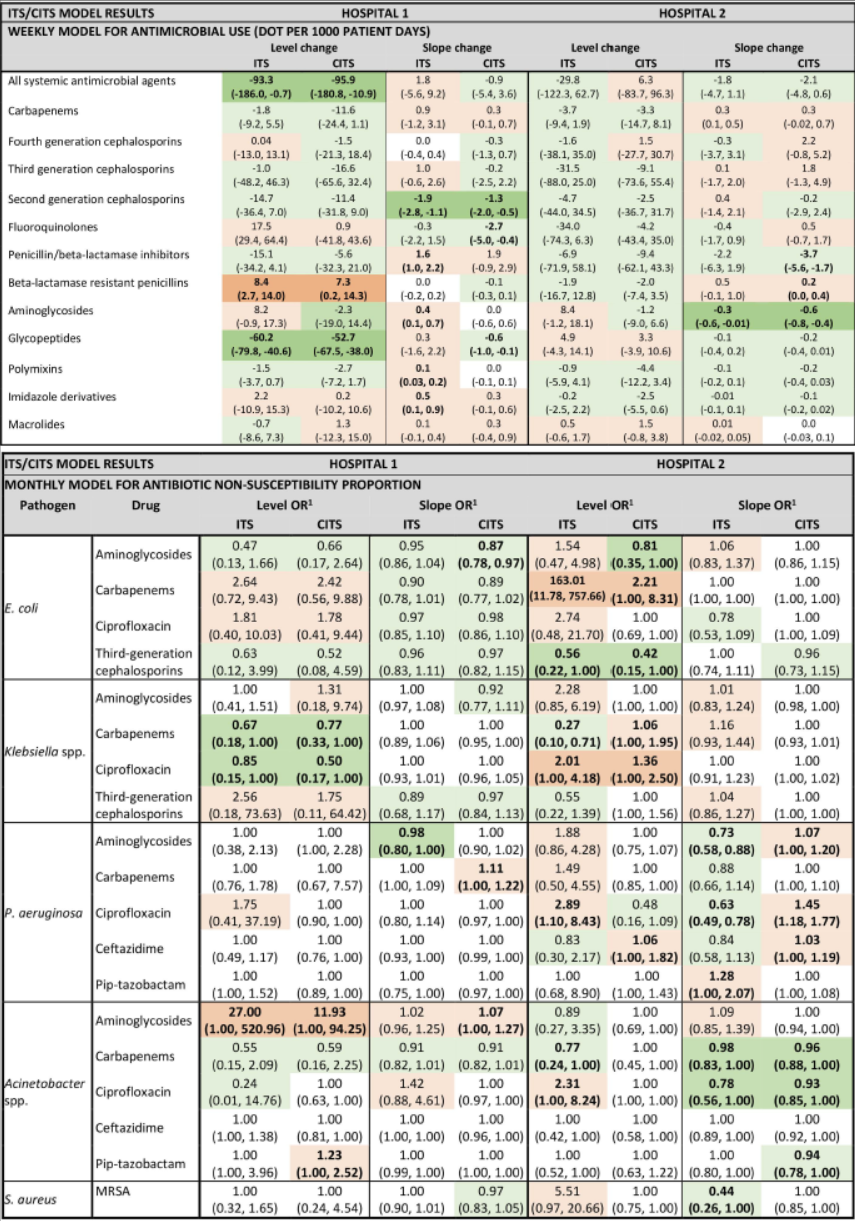


## Table S15. Results of ITS and controlled ITS models for antibiotic use in DOT per 1000 patient days

Note: ITS – I models are ITS models for the intervention time-series only, ITS – C models are ITS models for the control time-series only, CITS-I model estimates intervention group adjusting for control time-series; Lower and upper values are of the 95% confidence interval of the amount of change estimated from each model; Values highlighted in **bold face** were those with 95% confidence interval does not include 0.

| **Model**  Antibiotic use (DOT1000) | **Data series** | **Hospital 1** | | | | | | **Hospital 2** | | | | | |
| --- | --- | --- | --- | --- | --- | --- | --- | --- | --- | --- | --- | --- | --- |
|  |  | **Immediate change in DOT (first week)** | | | **Change in DOT per week (slope)** | | | **Immediate change in DOT (first week)** | | | **Change in DOT per week (slope)** | | |
|  |  | **Amount** | **Lower** | **Upper** | **Amount** | **Lower** | **Upper** | **Amount** | **Lower** | **Upper** | **Amount** | **Lower** | **Upper** |
| **Antibiotic use** |  |  |  |  |  |  |  |  |  |  |  |  |  |
| All antibiotics | ITS - I | **-93.3** | **-186.0** | **-0.7** | 1.8 | -5.6 | 9.2 | -29.8 | -122.3 | 62.7 | -1.8 | -4.7 | 1.1 |
|  | ITS - C | -16.5 | -97.9 | 64.9 | 1.6 | -6.9 | 10.0 | 38.2 | -46.0 | 122.5 | 0.5 | -2.1 | 3.2 |
|  | CITS - I | **-95.9** | **-180.8** | **-10.9** | -0.9 | -5.4 | 3.6 | 6.3 | -83.7 | 96.3 | -2.1 | -4.8 | 0.6 |
| ***By antibiotic subgroups*** |  |  |  |  |  |  |  |  |  |  |  |  |  |
| Carbapenem | ITS - I | -1.8 | -9.2 | 5.5 | 0.9 | -1.2 | 3.1 | -3.7 | -9.4 | 1.9 | **0.3** | **0.1** | **0.5** |
|  | ITS - C | 7.3 | -12.9 | 27.6 | 0.9 | -1.2 | 3.1 | -3.2 | -16.0 | 9.6 | **0.6** | **0.2** | **1.0** |
|  | CITS - I | -11.6 | -24.4 | 1.1 | 0.3 | -0.1 | 0.7 | -3.3 | -14.7 | 8.1 | 0.3 | -0.02 | 0.7 |
| 4G cephalosporin | ITS - I | 0.04 | -13.0 | 13.1 | 0.01 | -0.4 | 0.4 | -1.6 | -38.1 | 35.0 | -0.3 | -3.7 | 3.1 |
|  | ITS - C | 20.1 | -4.9 | 45.0 | -1.0 | -2.1 | 0.1 | -0.1 | -6.3 | 6.0 | -0.1 | -0.3 | 0.07 |
|  | CITS - I | -1.5 | -21.3 | 18.4 | -0.3 | -1.3 | 0.7 | 1.5 | -27.7 | 30.7 | 2.2 | -0.8 | 5.2 |
| 3G cephalosporin | ITS - I | -1.0 | -48.2 | 46.3 | 1.0 | -0.6 | 2.6 | -31.5 | -88.0 | 25.0 | 0.1 | -1.7 | 2.0 |
|  | ITS - C | 11.5 | -32.6 | 55.6 | 2.3 | -3.2 | 7.9 | 7.5 | -30.6 | 45.7 | **-1.4** | **-2.6** | **-0.2** |
|  | CITS - I | -16.6 | -65.6 | 32.4 | -0.2 | -2.5 | 2.2 | -9.1 | -73.6 | 55.4 | 1.8 | -1.3 | 4.9 |
| 2G cephalosporin | ITS - I | -14.7 | -36.4 | 7.0 | **-1.9** | **-2.8** | **-1.1** | -4.7 | -44.0 | 34.5 | 0.4 | -1.4 | 2.1 |
|  | ITS - C | 5.0 | -11.1 | 21.1 | -0.3 | -0.8 | 0.2 | 16.4 | -3.5 | 36.2 | -0.1 | -0.8 | 0.5 |
|  | CITS - I | -11.4 | -31.8 | 9.0 | **-1.3** | **-2.0** | **-0.5** | -2.5 | -36.7 | 31.7 | -0.2 | -2.9 | 2.4 |
| Fluoroquinolones | ITS - I | 17.5 | -29.4 | 64.4 | -0.3 | -2.2 | 1.5 | -34.0 | -74.3 | 6.3 | -0.4 | -1.7 | 0.9 |
|  | ITS - C | 7.7 | -25.4 | 40.8 | **-1.3** | **-2.5** | **-0.1** | -23.5 | -56.3 | 9.4 | 0.9 | -0.2 | 1.2 |
|  | CITS - I | 0.9 | -41.8 | 43.6 | **-2.7** | **-5.0** | **-0.4** | -4.2 | -43.4 | 35.0 | 0.5 | -0.7 | 1.7 |
| Penicillin/ beta-lactamase inhibitors | ITS - I | -15.1 | -34.2 | 4.1 | **1.6** | **1.0** | **2.2** | -6.9 | -71.9 | 58.1 | -2.2 | -6.3 | 1.9 |
|  | ITS - C | -12.1 | -37.5 | 13.3 | 0.5 | -0.2 | 1.3 | 11.0 | -40.3 | 62.2 | 1.2 | -0.5 | 3.0 |
|  | CITS - I | -5.6 | -32.3 | 21.0 | **1.9** | **0.9** | **2.9** | -9.4 | -62.1 | 43.3 | **-3.7** | **-5.6** | **-1.7** |
| Beta-lactamase resistant penicillin | ITS - I | **8.4** | **2.7** | **14.0** | 0.02 | -0.2 | 0.2 | -1.9 | -16.7 | 12.8 | 0.5 | -0.1 | 1.0 |
|  | ITS - C | **13.4** | **6.0** | **20.7** | 0.1 | -0.1 | 0.3 | -6.1 | -12.3 | 0.1 | **0.3** | **0.1** | **0.5** |
|  | CITS - I | **7.3** | **0.2** | **14.3** | -0.1 | -0.3 | 0.1 | -2.0 | -7.4 | 3.5 | **0.2** | **0.0** | **0.4** |
| Aminoglycosides | ITS - I | 8.2 | -0.9 | 17.3 | **0.4** | **0.1** | **0.7** | 8.4 | -1.2 | 18.1 | **-0.3** | **-0.6** | **-0.01** |
|  | ITS - C | 4.0 | -18.4 | 26.5 | 0.5 | -0.9 | 1.9 | **8.5** | **3.2** | **13.9** | **-0.2** | **-0.4** | **-0.04** |
|  | CITS - I | -2.3 | -19.0 | 14.4 | -0.01 | -0.6 | 0.6 | -1.2 | -9.0 | 6.6 | **-0.6** | **-0.8** | **-0.4** |
| Glycopeptide antibacterials | ITS - I | **-60.2** | **-79.8** | **-40.6** | 0.3 | -1.6 | 2.2 | 4.9 | -4.3 | 14.1 | -0.1 | -0.4 | 0.2 |
|  | ITS - C | **-31.3** | **-45.6** | **-17.0** | 0.04 | -0.4 | 0.5 | **7.5** | **2.7** | **12.4** | **-0.4** | **-0.5** | **-0.2** |
|  | CITS - I | **-52.7** | **-67.5** | **-38.0** | **-0.6** | **-1.0** | **-0.1** | 3.3 | -3.9 | 10.6 | -0.2 | -0.4 | 0.01 |
| Polymyxins | ITS - I | -1.5 | -3.7 | 0.7 | **0.1** | **0.03** | **0.2** | -0.9 | -5.9 | 4.1 | -0.1 | -0.2 | 0.1 |
|  | ITS - C | 0.6 | -3.9 | 5.1 | 0.03 | -0.1 | 0.2 | -8.1 | -19.0 | 2.8 | 0.1 | -0.6 | 0.8 |
|  | CITS - I | -2.7 | -7.2 | 1.7 | -0.01 | -0.1 | 0.1 | -4.4 | -12.2 | 3.4 | -0.2 | -0.4 | 0.03 |
| Imidazole derivatives | ITS - I | 2.2 | -10.9 | 15.3 | **0.5** | **0.1** | **0.9** | -0.2 | -2.5 | 2.2 | -0.01 | -0.1 | 0.1 |
|  | ITS - C | -0.2 | -4.4 | 4.0 | 0.1 | -0.01 | 0.2 | -0.2 | -3.2 | 2.8 | 0.01 | -0.1 | 0.1 |
|  | CITS - I | 0.2 | -10.2 | 10.6 | 0.3 | -0.1 | 0.6 | -2.5 | -5.5 | 0.6 | -0.1 | -0.2 | 0.02 |
| Macrolides | ITS - I | -0.7 | -8.6 | 7.3 | 0.1 | -0.1 | 0.4 | 0.5 | -0.6 | 1.7 | 0.014 | -0.024 | 0.051 |
|  | ITS - C | **19.7** | **3.0** | **36.5** | 0.5 | -1.3 | 2.3 | 0.6 | -2.2 | 3.4 | -0.03 | -0.1 | 0.1 |
|  | CITS - I | 1.3 | -12.3 | 15.0 | 0.3 | -0.4 | 0.9 | 1.5 | -0.8 | 3.8 | 0.03 | -0.03 | 0.1 |
| ***By study ward pairs*** |  |  |  |  |  |  |  |  |  |  |  |  |  |
| ICU pair | ITS - I | -86.3 | -177.3 | 4.8 | **3.7** | **0.9** | **6.5** | -96.8 | -288.1 | 83.4 | -2.2 | -8.1 | 3.7 |
|  | ITS - C | -107.9 | -240.0 | 23.9 | 3.0 | -1.6 | 7.7 | 24.1 | -250.3 | 298.4 | 6.3 | -7.0 | 19.6 |
|  | CITS - I | -128.0 | -258.2 | 2.2 | 4.5 | -0.6 | 9.6 | 70.1 | -199.1 | 339.3 | -3.0 | -20.4 | 14.5 |
| Surgical pair | ITS - I | **-156.1** | **-256.0** | **-56.3** | 2.1 | -2.3 | 6.4 | -41.2 | -120.1 | 37.7 | -0.7 | -3.2 | 1.8 |
|  | ITS - C | 57.7 | -146.7 | 262.2 | 5.1 | -15.4 | 25.6 | -25.6 | -214.1 | 162.8 | 3.8 | -2.3 | 10.0 |
|  | CITS - I | **-213.0** | **-351.3** | **-74.8** | -0.2 | -4.6 | 4.2 | -27.7 | -202.8 | 147.4 | -3.2 | -10.2 | 3.9 |
| Internal pair 1 | ITS - I | -86.1 | -247.7 | 75.6 | -1.6 | -12.5 | 9.3 | 44.5 | -37.1 | 126.0 | **-2.8** | **-5.3** | **-0.2** |
|  | ITS - C | 8.5 | -97.8 | 114.8 | 3.9 | -8.2 | 16.1 | -7.2 | -35.8 | 21.3 | 0.1 | -0.8 | 1.0 |
|  | CITS - I | -137.3 | -278.1 | 3.5 | -2.9 | -9.7 | 3.9 | **87.6** | **16.0** | **159.2** | -1.7 | -3.9 | 0.6 |
| Internal pair 2 | ITS - I | 61.6 | -69.4 | 192.7 | -0.8 | -5.1 | 3.6 | -31.6 | -132.2 | 68.9 | -0.2 | -3.3 | 2.9 |
|  | ITS - C | 61.9 | -2.7 | 126.5 | **-3.1** | **-5.0** | **-1.1** | 59.6 | -8.1 | 127.3 | 1.3 | -0.8 | 3.4 |
|  | CITS - I | **127.9** | **6.2** | **249.6** | -0.4 | -5.0 | 4.2 | -9.5 | -108.4 | 89.5 | 0.3 | -2.7 | 3.2 |

## Table S16. Results of ITS and controlled ITS models for monthly non-susceptibility proportions (for hospital-acquired isolates only)

Note: ITS – I models are ITS models for the intervention time-series only, ITS – C models are ITS models for the control time-series only, CITS-I model estimates intervention group adjusting for control; Lower and upper values are of the 95% confidence interval of the amount of change estimated from each model; OR: Odds ratio between non-susceptibility vs susceptibility; Values highlighted in **bold face** were those with 95% confidence interval does not include 1.

| **Model**  Monthly non-susceptibility proportions | **Data series** | **Hospital 1** | | | | | | **Hospital 2** | | | | | |
| --- | --- | --- | --- | --- | --- | --- | --- | --- | --- | --- | --- | --- | --- |
|  |  | **Immediate change in OR of non-susceptibility (level)** | | | **Change in OR of non-susceptibility per month (slope)** | | | **Immediate change in OR of non-susceptibility (level)** | | | **Change in OR of non-susceptibility per month (slope)** | | |
|  |  | **Amount** | **Lower** | **Upper** | **Amount** | **Lower** | **Upper** | **Amount** | **Lower** | **Upper** | **Amount** | **Lower** | **Upper** |
| ***Escherichia coli*** |  |  |  |  |  |  |  |  |  |  |  |  |  |
| Aminoglycoside | ITS - I | 0.47 | 0.13 | 1.66 | 0.95 | 0.86 | 1.04 | 1.54 | 0.47 | 4.98 | 1.06 | 0.83 | 1.37 |
|  | ITS - C | 0.34 | 0.09 | 1.27 | 1.00 | 0.88 | 1.13 | **0.49** | **0.12** | **1.00** | **1.40** | **1.07** | **1.86** |
|  | CITS - I | 0.66 | 0.17 | 2.64 | **0.87** | **0.78** | **0.97** | **0.81** | **0.35** | **1.00** | 1.00 | 0.86 | 1.15 |
| Carbapenem | ITS - I | 2.64 | 0.72 | 9.43 | 0.90 | 0.78 | 1.01 | **163.01** | **11.78** | **757.66** | 1.00 | 1.00 | 1.00 |
|  | ITS - C | 1.11 | 0.35 | 3.52 | 0.94 | 0.83 | 1.05 | 1.00 | 1.00 | 4.47 | **0.88** | **0.62** | **1.00** |
|  | CITS – I | 2.42 | 0.56 | 9.88 | 0.89 | 0.77 | 1.02 | **2.21** | **1.00** | **8.31** | 1.00 | 1.00 | 1.00 |
| Ciprofloxacin | ITS - I | 1.81 | 0.40 | 10.03 | 0.97 | 0.85 | 1.10 | 2.74 | 0.48 | 21.70 | 0.78 | 0.53 | 1.09 |
|  | ITS - C | 15.63 | 1.5 | 474.36 | **0.81** | **0.63** | **0.99** | **0.13** | **0.01** | **1.00** | 1.00 | 0.83 | 1.26 |
|  | CITS – I | 1.78 | 0.41 | 9.44 | 0.98 | 0.86 | 1.10 | 1.00 | 0.69 | 1.00 | 1.00 | 1.00 | 1.09 |
| Third-generation cephalosporin | ITS - I | 0.63 | 0.12 | 3.99 | 0.96 | 0.83 | 1.11 | **0.56** | **0.22** | **1.00** | 1.00 | 0.74 | 1.11 |
|  | ITS - C | 0.40 | 0.03 | 16.34 | 0.92 | 0.68 | 1.29 | 0.27 | 0.06 | 2.22 | **0.77** | **0.42** | **0.94** |
|  | CITS – I | 0.52 | 0.08 | 4.59 | 0.97 | 0.82 | 1.15 | **0.42** | **0.15** | **1.00** | 0.96 | 0.73 | 1.15 |
| ***Klebsiella* spp.** |  |  |  |  |  |  |  |  |  |  |  |  |  |
| Aminoglycoside | ITS - I | 1.00 | 0.41 | 1.51 | 1.00 | 0.97 | 1.08 | 2.28 | 0.85 | 6.19 | 1.01 | 0.83 | 1.24 |
|  | ITS - C | 2.74 | 0.67 | 12.94 | 0.91 | 0.81 | 1.04 | 1.31 | 0.32 | 5.29 | 1.22 | 0.95 | 1.57 |
|  | CITS - I | 1.31 | 0.18 | 9.74 | 0.92 | 0.77 | 1.11 | 1.00 | 1.00 | 1.00 | 1.00 | 0.98 | 1.00 |
| Carbapenem | ITS - I | 0.67 | 0.18 | 1.00 | 1.00 | 0.89 | 1.06 | **0.27** | **0.10** | **0.71** | 1.16 | 0.93 | 1.44 |
|  | ITS - C | 1.15 | 0.31 | 4.37 | 0.99 | 0.89 | 1.10 | 0.90 | 0.22 | 3.53 | 1.13 | 0.90 | 1.43 |
|  | CITS - I | **0.77** | **0.33** | **1.00** | 1.00 | 0.95 | 1.00 | **1.06** | **1.00** | **1.95** | 1.00 | 0.93 | 1.01 |
| Ciprofloxacin | ITS - I | **0.85** | **0.15** | **1.00** | 1.00 | 0.93 | 1.01 | **2.01** | **1.00** | **4.18** | 1.00 | 0.91 | 1.23 |
|  | ITS - C | **4.02** | **1.24** | **14.80** | 0.94 | 0.85 | 1.04 | 1.00 | 0.79 | 1.43 | 1.00 | 1.00 | 1.09 |
|  | CITS - I | **0.50** | **0.17** | **1.00** | 1.00 | 0.96 | 1.05 | **1.36** | **1.00** | **2.50** | 1.00 | 1.00 | 1.02 |
| Third-generation cephalosporin | ITS - I | 2.56 | 0.18 | 73.63 | 0.89 | 0.68 | 1.17 | 0.55 | 0.22 | 1.39 | 1.04 | 0.86 | 1.27 |
|  | ITS - C | 1.22 | 0.21 | 11.16 | 0.96 | 0.80 | 1.17 | 1.00 | 0.61 | 2.63 | 1.00 | 0.73 | 1.02 |
|  | CITS - I | 1.75 | 0.11 | 64.42 | 0.97 | 0.84 | 1.13 | 1.00 | 1.00 | 1.56 | 1.00 | 1.00 | 1.00 |
| ***Pseudomonas aeruginosa*** |  |  |  |  |  |  |  |  |  |  |  |  |  |
| Aminoglycoside | ITS - I | 1.00 | 0.38 | 2.13 | **0.98** | **0.80** | **1.00** | 1.88 | 0.86 | 4.28 | **0.73** | **0.58** | **0.88** |
|  | ITS - C | 0.11 | 0.01 | 1.22 | 0.91 | 0.76 | 1.12 | 1.00 | 0.41 | 1.00 | 1.00 | 0.90 | 1.13 |
|  | CITS - I | 1.00 | 1.00 | 2.28 | 1.00 | 0.90 | 1.02 | 1.00 | 0.75 | 1.07 | **1.07** | **1.00** | **1.20** |
| Carbapenem | ITS - I | 1.00 | 0.76 | 1.78 | 1.00 | 1.00 | 1.09 | 1.49 | 0.50 | 4.55 | 0.88 | 0.66 | 1.14 |
|  | ITS - C | **0.13** | **0.03** | **0.57** | **0.88** | **0.79** | **0.98** | 1.00 | 0.31 | 1.05 | 1.00 | 0.66 | 1.21 |
|  | CITS - I | 1.00 | 0.67 | 7.57 | **1.11** | **1.00** | **1.22** | 1.00 | 0.85 | 1.00 | 1.00 | 1.00 | 1.10 |
| Ciprofloxacin | ITS - I | 1.75 | 0.41 | 37.19 | 1.00 | 0.80 | 1.14 | **2.89** | **1.10** | **8.43** | **0.63** | **0.49** | **0.78** |
|  | ITS - C | 0.83 | 0.11 | 6.15 | 1.12 | 0.96 | 1.35 | 1.00 | 0.53 | 1.00 | 1.00 | 0.83 | 1.18 |
|  | CITS - I | 1.00 | 0.90 | 1.00 | 1.00 | 0.97 | 1.00 | 0.48 | 0.16 | 1.09 | **1.45** | **1.18** | **1.77** |
| Ceftazidime | ITS - I | 1.00 | 0.49 | 1.17 | 1.00 | 0.93 | 1.00 | 0.83 | 0.30 | 2.17 | 0.84 | 0.58 | 1.13 |
|  | ITS - C | 1.13 | 0.15 | 8.76 | 0.98 | 0.85 | 1.14 | 1.00 | 1.00 | 2.71 | 1.00 | 1.00 | 1.04 |
|  | CITS - I | 1.00 | 0.76 | 1.00 | 1.00 | 0.99 | 1.00 | **1.06** | **1.00** | **1.82** | **1.03** | **1.00** | **1.19** |
| Piperacillin-tazobactam | ITS - I | 1.00 | 1.00 | 1.52 | 1.00 | 0.75 | 1.00 | 1.00 | 0.68 | 8.90 | **1.28** | **1.00** | **2.07** |
|  | ITS – C | 1.00 | 1.00 | 2.36 | 1.00 | 0.80 | 1.00 | 1.00 | 1.00 | 2.36 | 1.00 | 0.80 | 1.00 |
|  | CITS - I | 1.00 | 0.89 | 1.00 | 1.00 | 0.97 | 1.00 | 1.00 | 1.00 | 1.43 | 1.00 | 1.00 | 1.08 |
| ***Acinetobacter* spp.** |  |  |  |  |  |  |  |  |  |  |  |  |  |
| Aminoglycoside | ITS - I | **27.00** | **1.00** | **520.96** | 1.02 | 0.96 | 1.25 | 0.89 | 0.27 | 3.35 | 1.09 | 0.85 | 1.39 |
|  | ITS - C | 0.03 | 0.00 | 20.43 | 0.86 | 0.57 | 1.33 | 1.00 | 0.31 | 1.90 | 1.00 | 0.85 | 1.14 |
|  | CITS - I | **11.93** | **1.00** | **94.25** | **1.07** | **1.00** | **1.27** | 1.00 | 0.69 | 1.00 | 1.00 | 0.94 | 1.00 |
| Carbapenem | ITS - I | 0.55 | 0.15 | 2.09 | 0.91 | 0.82 | 1.01 | **0.77** | **0.24** | **1.00** | **0.98** | **0.83** | **1.00** |
|  | ITS - C | **0.10** | **0.03** | **0.40** | 0.97 | 0.85 | 1.10 | 1.00 | 0.38 | 1.00 | 1.00 | 0.88 | 1.00 |
|  | CITS - I | 0.59 | 0.16 | 2.25 | 0.91 | 0.82 | 1.01 | 1.00 | 0.45 | 1.00 | **0.96** | **0.88** | **1.00** |
| Ciprofloxacin | ITS - I | 0.24 | 0.01 | 14.76 | 1.42 | 0.88 | 4.61 | **2.31** | **1.00** | **8.24** | **0.78** | **0.56** | **1.00** |
|  | ITS - C | 1.00 | 0.16 | 2.82 | 1.00 | 0.93 | 1.13 | 1.00 | 0.31 | 2.19 | 1.00 | 0.88 | 1.00 |
|  | CITS - I | 1.00 | 0.63 | 1.00 | 1.00 | 0.97 | 1.00 | 1.00 | 1.00 | 1.00 | **0.93** | **0.85** | **1.00** |
| Ceftazidime | ITS - I | 1.00 | 1.00 | 1.38 | 1.00 | 1.00 | 1.00 | 1.00 | 0.42 | 1.00 | 1.00 | 0.89 | 1.00 |
|  | ITS - C | 1.00 | 0.36 | 1.00 | 1.00 | 0.96 | 1.00 | 1.00 | 0.40 | 1.11 | 1.00 | 0.90 | 1.00 |
|  | CITS - I | 1.00 | 0.81 | 1.00 | 1.00 | 0.96 | 1.00 | 1.00 | 0.58 | 1.00 | 1.00 | 0.92 | 1.00 |
| Piperacillin-tazobactam | ITS - I | 1.00 | 1.00 | 3.96 | 1.00 | 0.99 | 1.00 | 1.00 | 0.52 | 1.00 | 1.00 | 0.80 | 1.00 |
|  | ITS - C | 1.00 | 1.00 | 2.01 | 1.00 | 1.00 | 1.02 | 1.00 | 0.56 | 1.00 | 1.00 | 0.92 | 1.00 |
|  | CITS - I | **1.23** | **1.00** | **2.52** | 1.00 | 1.00 | 1.00 | 1.00 | 0.63 | 1.22 | **0.94** | **0.78** | **1.00** |
| ***Staphylococcus aureus*** |  |  |  |  |  |  |  |  |  |  |  |  |  |
| MRSA | ITS - I | 1.00 | 0.32 | 1.65 | 1.00 | 0.90 | 1.01 | 5.51 | 0.97 | 20.66 | **0.44** | **0.26** | **1.00** |
|  | ITS - C | **0.28** | **0.10** | **0.82** | 1.03 | 0.95 | 1.12 | 1.00 | 0.23 | 1.00 | 1.00 | 1.00 | 1.00 |
|  | CITS - I | 1.00 | 0.24 | 4.54 | 0.97 | 0.83 | 1.05 | 1.00 | 0.75 | 1.00 | 1.00 | 0.85 | 1.00 |

## Table S17. Results of ITS and controlled ITS models for in-hospital mortality

Note: ITS – I models are ITS models for the intervention time-series only, ITS – C models are ITS models for the control time-series only, CITS-I model estimates intervention group adjusting for control; Lower and upper values are of the 95% confidence interval of the amount of change estimated from each model; Values highlighted in **bold face** were those with 95% confidence interval does not include 0.

| **Model** | **Data**  **series** | **Hospital 1** | | | | | | **Hospital 2** | | | | | |
| --- | --- | --- | --- | --- | --- | --- | --- | --- | --- | --- | --- | --- | --- |
|  |  | **Level change** | | | **Slope** | | | **Level change** | | | **Slope** | | |
|  |  | **Amount** | **Lower** | **Upper** | **Amount** | **Lower** | **Upper** | **Amount** | **Lower** | **Upper** | **Amount** | **Lower** | **Upper** |
| All study wards | ITS - I | -0.47 | -1.97 | 1.02 | **0.09** | **0.04** | **0.13** | 1.38 | -4.92 | 7.69 | 0.01 | -0.18 | 0.21 |
|  | ITS - C | **-12.9** | **-22.2** | **-3.58** | -0.15 | -0.68 | 0.39 | -2.59 | -14.25 | 9.07 | 0.27 | -0.09 | 0.63 |
|  | CITS - I | -0.37 | -5.68 | 4.95 | 0.07 | -0.09 | 0.23 | 2.23 | -8.42 | 12.89 | 0.09 | -0.22 | 0.40 |
| ICU pair | ITS – I | -1.93 | -4.64 | 0.78 | **0.17** | **0.08** | **0.25** | -1.20 | -19.95 | 17.56 | **0.85** | **0.24** | **1.46** |
|  | ITS – C | -20.47 | -51.78 | 10.8 | 0.91 | -0.17 | 1.98 | -44.13 | -143.38 | 55.12 | **3.82** | **0.72** | **6.92** |
|  | CITS - I | 0.51 | -13.61 | 14.63 | 0.23 | -0.18 | 0.65 | -7.61 | -77.36 | 62.13 | 1.61 | -0.46 | 3.67 |
| Surgical pair | ITS – I | 0.22 | -1.32 | 1.75 | -0.02 | -0.07 | 0.03 | **-2.48** | **-4.94** | **-0.02** | 0.05 | -0.02 | 0.13 |
|  | ITS – C | -0.76 | -3.48 | 1.97 | 0.00 | -0.08 | 0.09 | 3.78 | -1.50 | 9.06 | -0.03 | -0.20 | 0.13 |
|  | CITS - I | 0.82 | -1.87 | 3.52 | 0.00 | -0.08 | 0.07 | -4.00 | -8.69 | 0.68 | 0.01 | -0.12 | 0.14 |
| Internal pair 1 | ITS – I | -0.21 | -0.74 | 0.33 | 0.01 | -0.01 | 0.02 | -0.71 | -4.66 | 3.25 | 0.01 | -0.11 | 0.13 |
|  | ITS – C | -0.06 | -8.95 | 8.82 | 0.12 | -0.15 | 0.39 | -5.7 | -11.47 | 0.07 | 0.00 | -0.18 | 0.18 |
|  | CITS - I | -5.94 | -13.36 | 1.48 | -0.04 | -0.26 | 0.17 | -0.15 | -6.07 | 5.77 | 0.02 | -0.15 | 0.19 |
| Internal pair 2 | ITS – I | -0.30 | -2.99 | 2.40 | 0.00 | -0.08 | 0.08 | -1.09 | -7.37 | 5.2 | 0.02 | -0.18 | 0.21 |
|  | ITS – C | -0.88 | -4.75 | 2.99 | 0.04 | -0.08 | 0.16 | -5.34 | -15.5 | 4.82 | 0.24 | -0.07 | 0.56 |
|  | CITS - I | -2.00 | -5.81 | 1.81 | -0.05 | -0.15 | 0.06 | 1.21 | -7.90 | 10.32 | 0.07 | -0.19 | 0.34 |

## Table S18. Results of ITS and controlled ITS models for cost of hospitalization

Note: ITS – I models are ITS models for the intervention time-series only, ITS – C models are ITS models for the control time-series only, CITS-I model estimates intervention group adjusting for control; Lower and upper values are of the 95% confidence interval of the amount of change estimated from each model; Values highlighted in **bold face** were those with 95% confidence interval does not include 0.

| **Model** | **Data**  **series** | **Hospital 1** | | | | | | **Hospital 2** | | | | | |
| --- | --- | --- | --- | --- | --- | --- | --- | --- | --- | --- | --- | --- | --- |
|  |  | **Level change** | | | **Slope** | | | **Level change** | | | **Slope** | | |
|  |  | **Amount** | **Lower** | **Upper** | **Amount** | **Lower** | **Upper** | **Amount** | **Lower** | **Upper** | **Amount** | **Lower** | **Upper** |
| All study wards | ITS – I | 7.7 | -36.2 | 51.6 | **-3.4** | **-4.6** | **-2.1** | -88.5 | -426.8 | 249.8 | -9.2 | -21.2 | 2.9 |
|  | ITS – C | 7.4 | -20.1 | 35.0 | -1.9 | -5.8 | 2.1 | 64.4 | -77.3 | 206.2 | -3.5 | -8.0 | 1.0 |
|  | CITS - I | -22.3 | -55.4 | 10.9 | -2.0 | -4.0 | 0.0 | -51.2 | -326.7 | 224.4 | -7.7 | -17.7 | 2.3 |
| ICU pair | ITS - I | -21.4 | -68.8 | 26.0 | -0.0 | -2.6 | 2.5 | -213.6 | -783.7 | 356.5 | -17.8 | -36.3 | 0.7 |
|  | ITS – C | -4.0 | -75.4 | 67.5 | -0.6 | -7.2 | 6.0 | -214.5 | -668.8 | 239.8 | -8.7 | -23.2 | 5.9 |
|  | CITS - I | -25.2 | -84.4 | 34.0 | -1.3 | -3.7 | 1.2 | -369.5 | -966.8 | 227.9 | -17.4 | -35.8 | 1.0 |
| Surgical pair | ITS – I | -39.0 | -110.6 | 32.6 | -3.2 | -7.9 | 1.4 | -113.4 | -348.4 | 121.6 | -2.9 | -10.3 | 4.6 |
|  | ITS – C | **134.2** | **62.8** | **205.6** | **-3.7** | **-5.9** | **-1.5** | 84.0 | -147.4 | 315.5 | -2.4 | -9.7 | 4.9 |
|  | CITS - I | -46.8 | -119.8 | 26.2 | **-5.5** | **-8.0** | **-3.1** | -126.7 | -393.2 | 140.0 | -4.9 | -13.5 | 3.5 |
| Internal pair 1 | ITS - I | **68.7** | **19.1** | **118.3** | **3.0** | **1.2** | **4.7** | -45.3 | -421.9 | 331.3 | -4.5 | -16.4 | 7.4 |
|  | ITS – C | -6.4 | -22.9 | 10.1 | 0.2 | -0.3 | 0.9 | 44.7 | -124.3 | 213.8 | -2.7 | -8.2 | 2.7 |
|  | CITS - I | -10.4 | -48.5 | 27.8 | -0.1 | -3.1 | 2.8 | 108.0 | -215.3 | 431.3 | 0.3 | -9.8 | 10.5 |
| Internal pair 2 | ITS - I | -7.9 | -44.3 | 28.5 | **1.1** | **0.0** | **2.2** | -308.9 | -876.6 | 258.9 | 1.1 | -16.6 | 18.8 |
|  | ITS – C | 20.3 | -4.9 | 45.5 | **-1.2** | **-2.0** | **-0.4** | 78.5 | -89.0 | 246.0 | -0.7 | -6.1 | 4.7 |
|  | CITS - I | -4.7 | -38.9 | 29.4 | 0.2 | -1.0 | 1.4 | -14.7 | -492.7 | 463.4 | 11.2 | -4.35 | 26.8 |

## Table S19. Number of patient admissions to ICUs and ICU in-hospital mortality by main ICD10 diagnosis groups in intervention and control groups in two hospitals before and after the start of AMS implementation

| **ICD10 Diagnosis Group** | **Time period** | **Hospital 1** | | | | **Hospital 2** | | | |
| --- | --- | --- | --- | --- | --- | --- | --- | --- | --- |
|  |  | **Intervention** | | **Control** | | **Intervention** | | **Control** | |
|  |  | Number of patients | Mortality per 1000 patients | Number of patients | Mortality per 1000 patients | Number of patients | Mortality per 1000 patients | Number of patients | Mortality per 1000 patients |
| Certain infectious and parasitic diseases | Before | 5 | 0.32 | 88 | **10.53** | 10 | **4.43** | 34 | **12.15** |
|  | After | 2 | 0.19 | 85 | **13.88** | 13 | **9.00** | 19 | 8.94 |
| Diseases of the circulatory system | Before | 10 | **0.64** | 379 | **45.34** | 130 | **57.62** | 109 | **38.94** |
|  | After | 9 | **0.85** | 363 | **59.29** | 96 | **66.48** | 86 | **40.47** |
| Diseases of the digestive system | Before | **8** | **0.51** | 89 | **10.65** | 53 | 23.49 | **26** | **9.29** |
|  | After | **10** | **0.94** | 73 | **11.92** | 19 | 13.16 | **36** | **16.94** |
| Diseases of the genitourinary system | Before | 0 | 0 | **15** | **1.79** | 3 | 1.33 | 6 | 2.14 |
|  | After | 0 | 0 | **32** | **5.23** | 0 | 0 | 4 | 1.88 |
| Diseases of the respiratory system | Before | **6** | **0.39** | 384 | **45.94** | 53 | **23.49** | 277 | **98.96** |
|  | After | **9** | **0.85** | 313 | **51.13** | 41 | **28.39** | 245 | **115.29** |
| Diseases of the skin and subcutaneous tissue | Before | 0 | 0 | 12 | **1.44** | 3 | 1.32 | 0 | 0 |
|  | After | 0 | 0 | 9 | **1.47** | 0 | 0 | 0 | 0 |
| Injury, poisoning and certain other consequences of external causes | Before | **2** | **0.13** | 15 | 1.79 | 70 | **31.03** | 39 | 13.93 |
|  | After | **6** | **0.57** | 5 | 0.82 | 50 | **34.63** | 28 | 13.18 |
| Neoplasms | Before | **1** | **0.06** | **39** | **4.67** | 21 | 9.31 | 16 | 5.72 |
|  | After | **2** | **0.19** | **41** | **6.70** | 8 | 5.54 | 2 | 0.94 |
| Symptoms, signs and abnormal clinical and laboratory findings, not elsewhere classified | Before | 45 | **2.89** | 255 | **30.51** | 87 | **38.56** | 482 | **172.20** |
|  | After | 41 | **3.86** | 244 | **39.86** | 77 | **53.32** | 368 | **173.18** |

## Figure S7. Weekly trends in antibiotic use (number of DOT per 1000 patient-days) in the intervention (blue line) and control group (black line) overall (left column) and stratified by study ward pair in each hospital (right column).

Note: Vertical broken line indicates the time point when AMS intervention started. Solid trend lines are observed data, broken trend lines are the predicted data based on the data observed before the start of AMS intervention. Upper row: Trends in hospital 1; the trend before the start of intervention fluctuated widely, and when the intervention start, there was a stiff decrease in the first week then fluctuating again to a smaller degree in the following weeks for the intervention group while the trend continued to fluctuate widely for the control group. Lower row: Trends in hospital 2. After the intervention start, there was a decreasing trend in antibiotic use in the intervention group compared to the counterfactual scenario, while the reverse was observed for the control group.

| 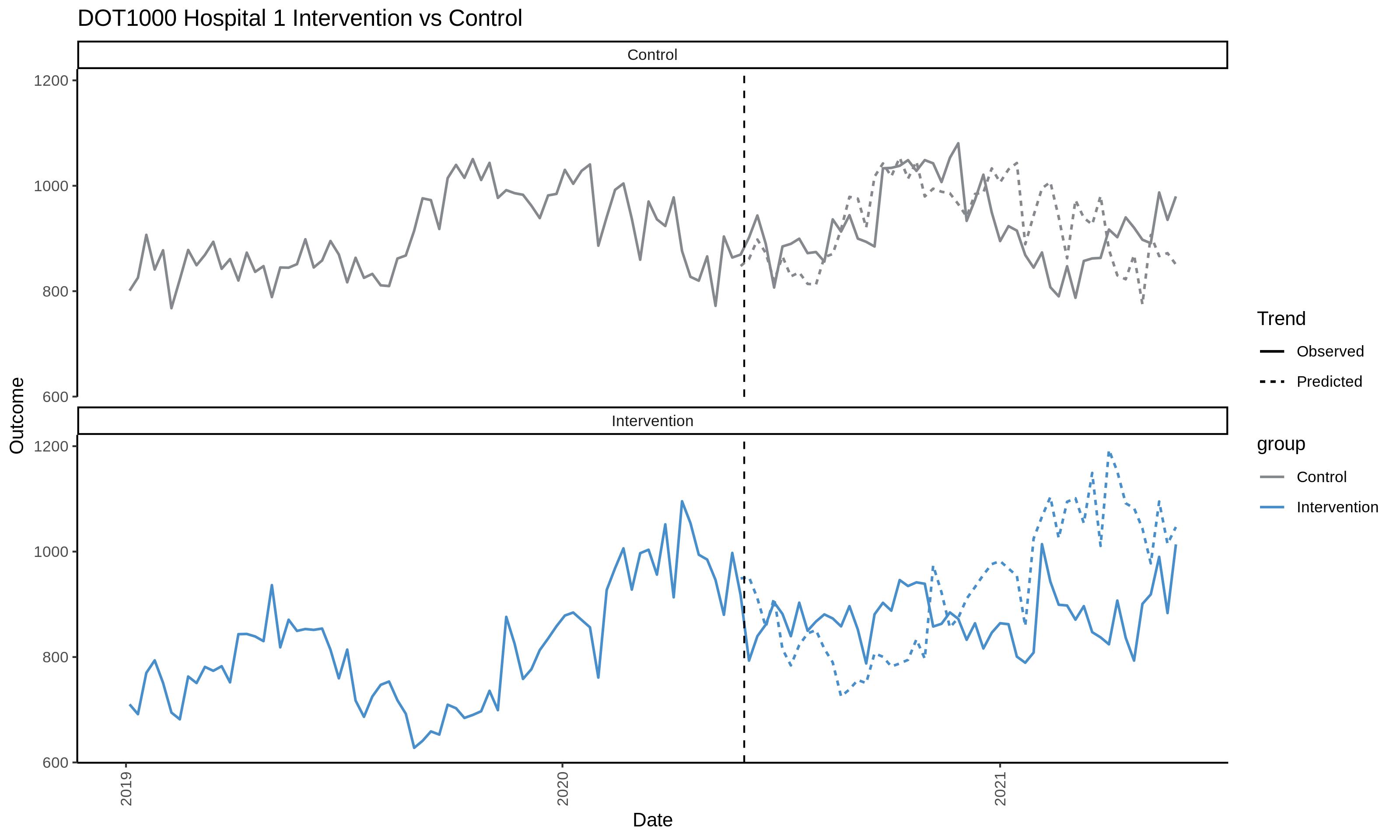 | 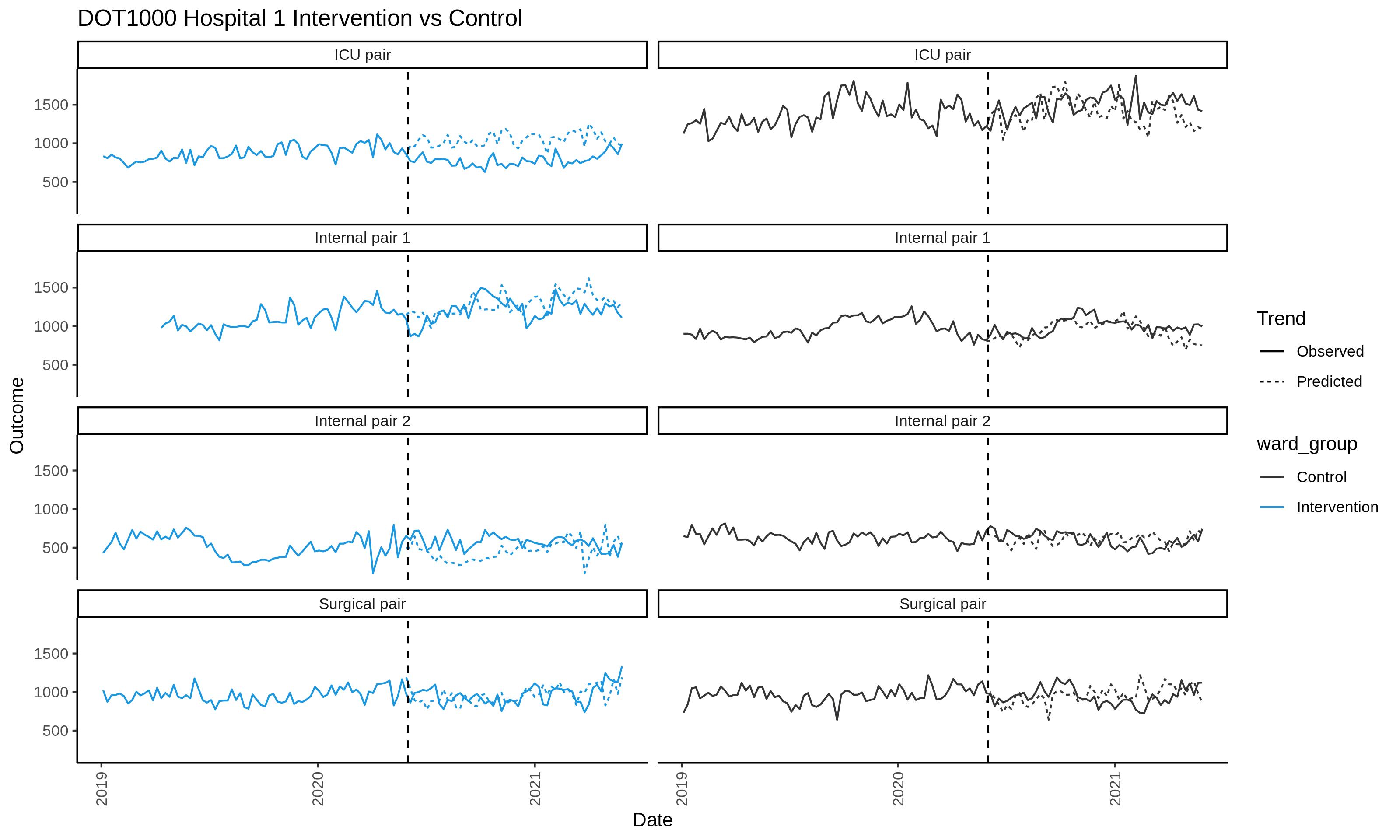 |
| --- | --- |
| 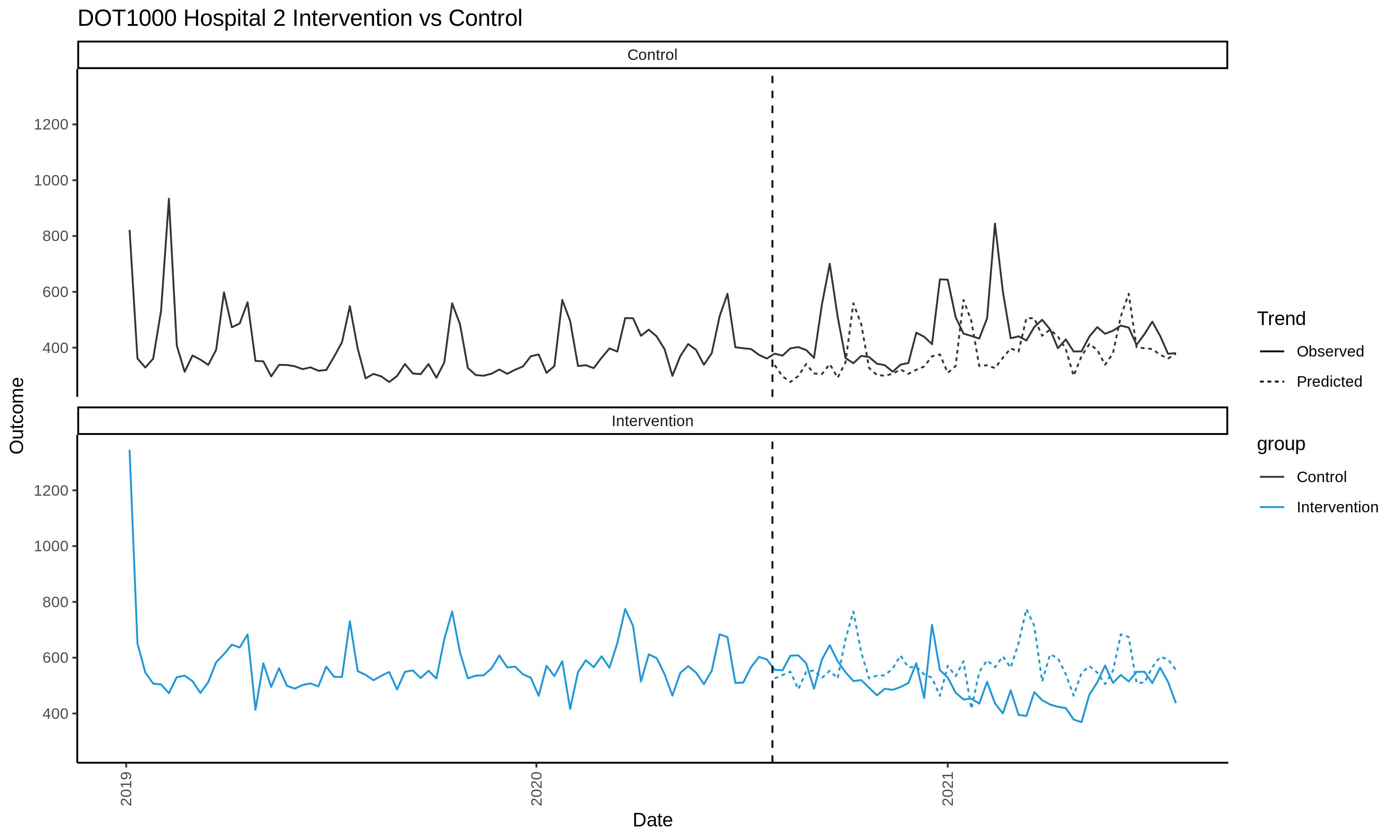 | 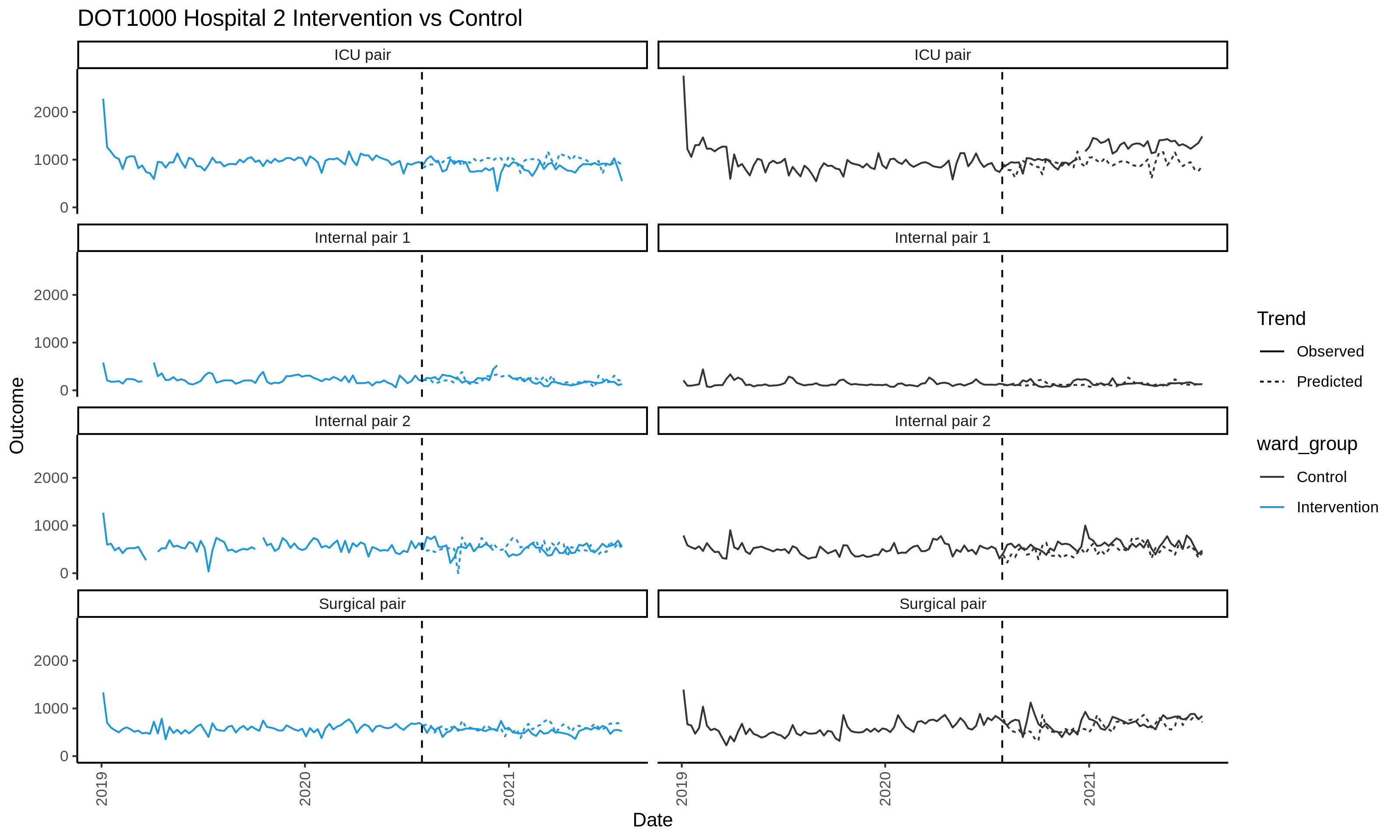 |

# **E. Model diagnostics**

## Table S20. Diagnostic plots for ITS/CITS ARIMA models for antibiotic use in DOT per 1000 patient days

Note: ITS – I models are ITS models for the intervention time-series only, ITS – C models are ITS models for the control time-series only, CITS-I model estimates intervention group adjusting for control. Segmented regression model assumptions were checked by examining the residuals, particularly temporal correlation using ACF/PACF plots and tests for residuals. Below are the results of checking the residuals. Models are considered to be valid when the residuals are approximately normally distributed and the residuals test returns a non-significant p value. All residual and test for autocorrelation of residuals were generated using checkresiduals() from the package forecast in R.

| **Model/**  **Data series** | | **Hospital 1** | | **Hospital 2** | |
| --- | --- | --- | --- | --- | --- |
|  |  | **Residuals from regression with ARIMA errors** | **Tests for residuals** | **Residuals from regression with ARIMA errors** | **Tests for residuals** |
| **Antibiotic DOT1000** | | | | | |
| All antibiotics | ITS – I | 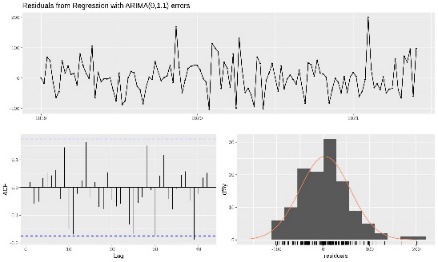 |  | 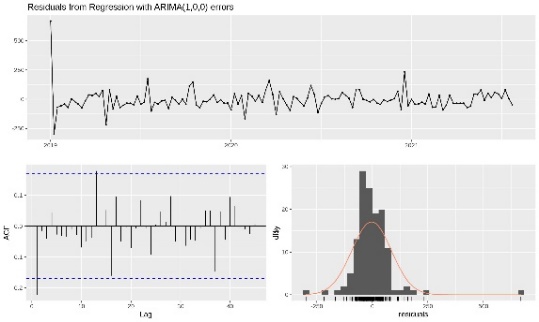 |  |
|  | ITS – C | 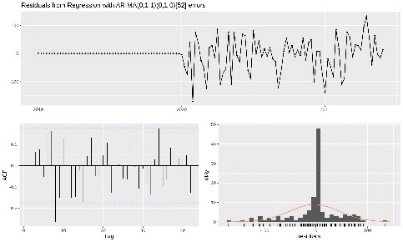 |  | 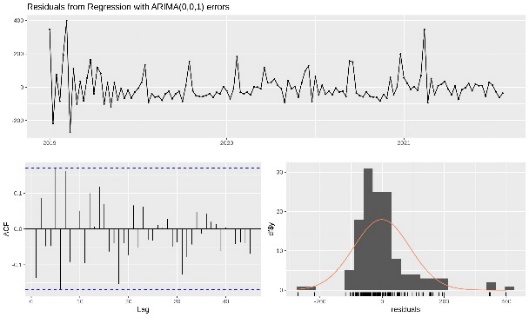 |  |
|  | CITS – I | 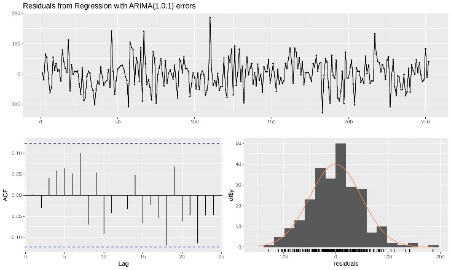 |  | 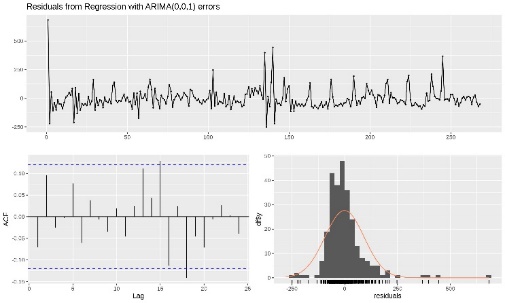 |  |
| ***DOT1000 by antibiotic subgroups*** | | | | | |
| Carbapenem | ITS - I | 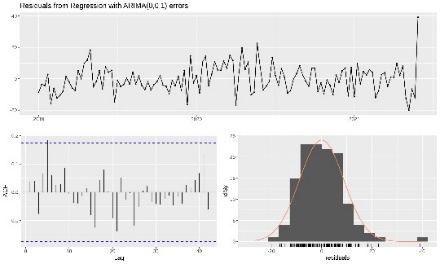 | 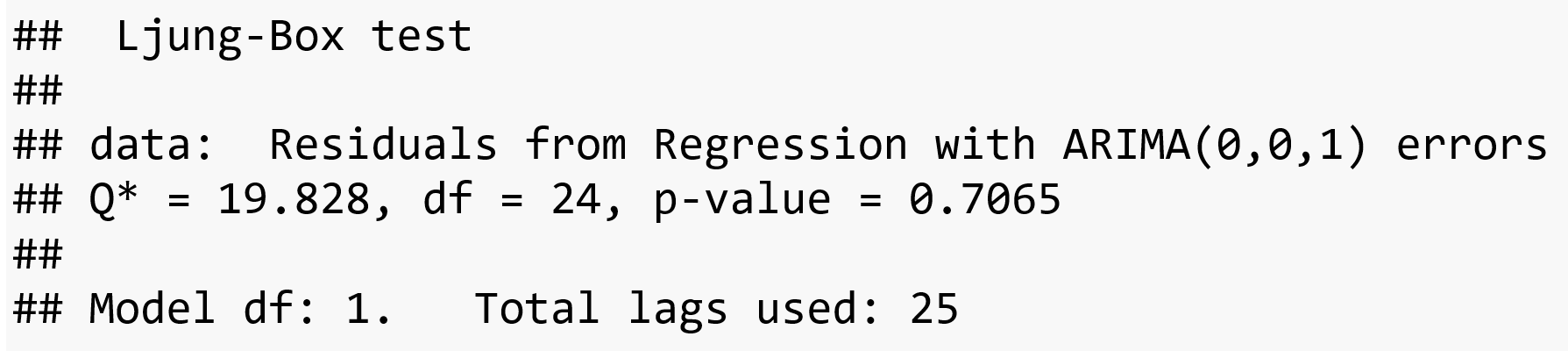 | 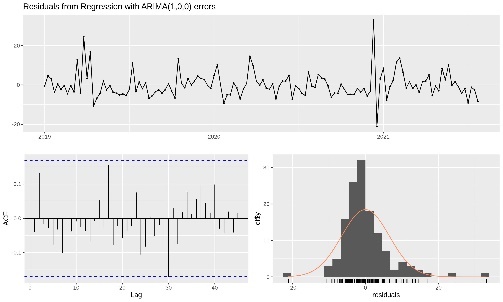 |  |
|  | ITS - C | 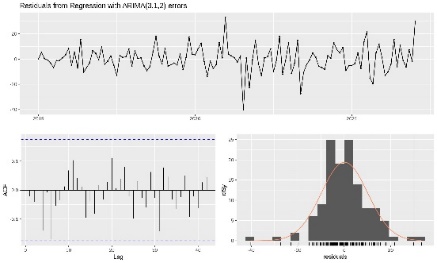 | 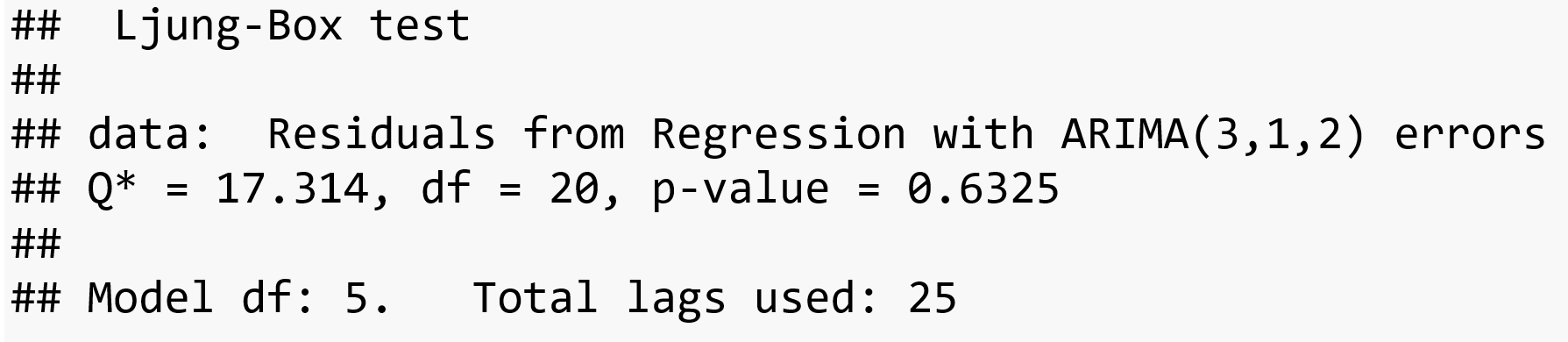 | 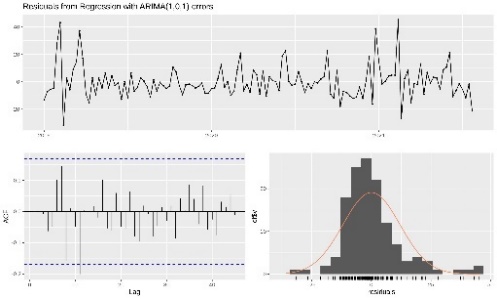 |  |
|  | CITS - I | 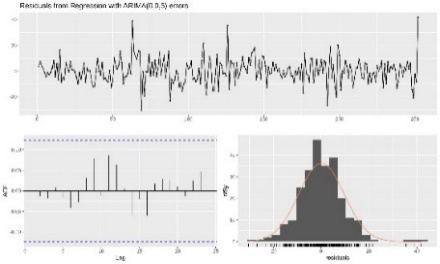 | 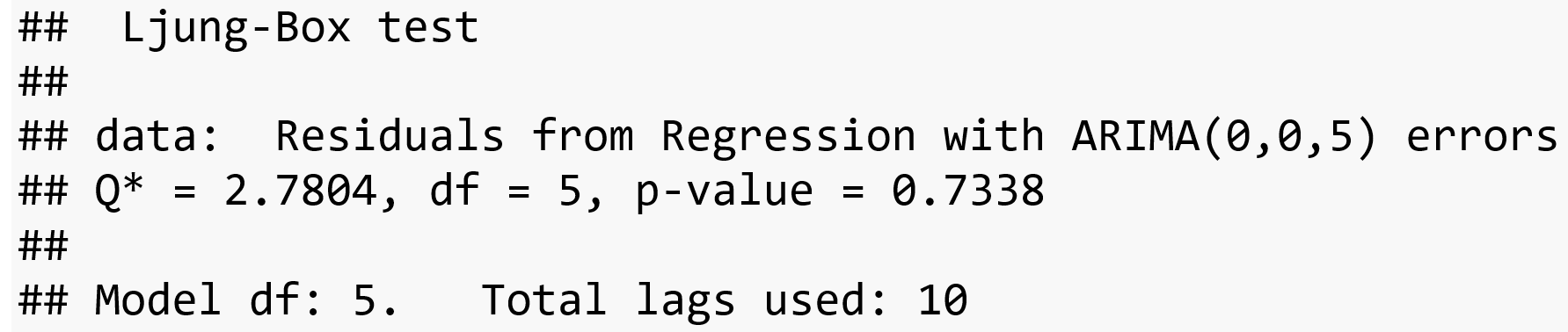 | 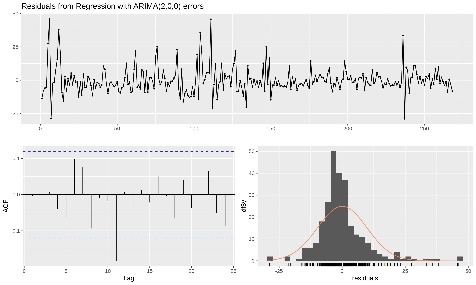 | 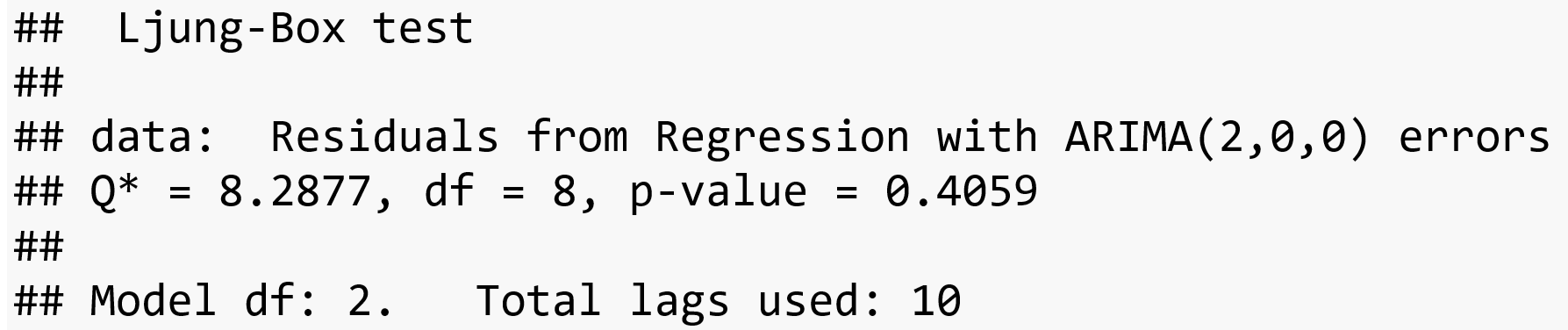 |
| 4G cephalosporin | ITS - I | 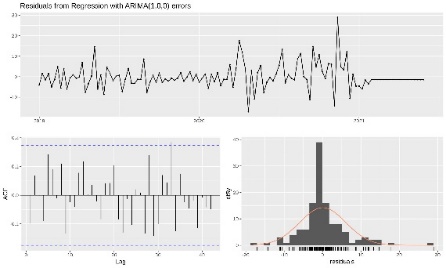 | 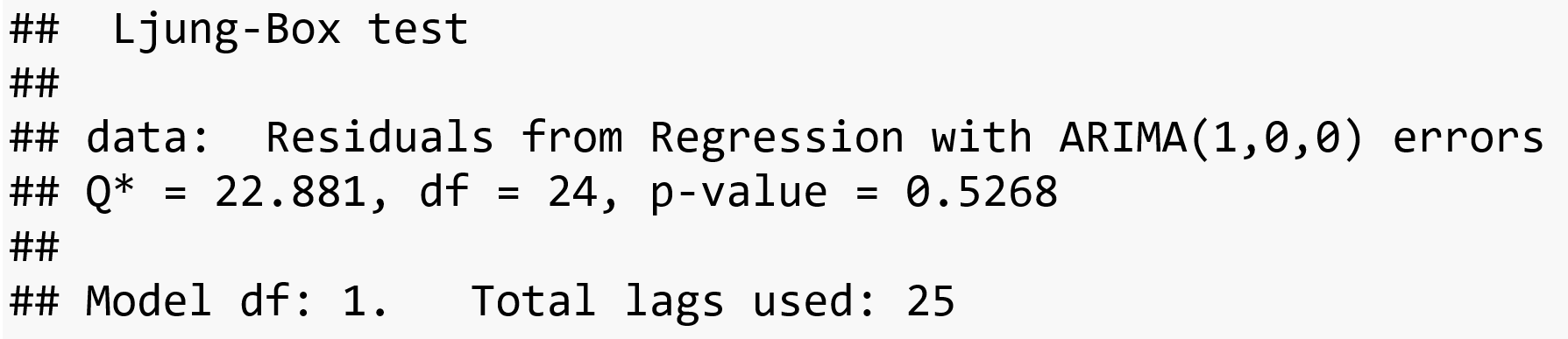 | 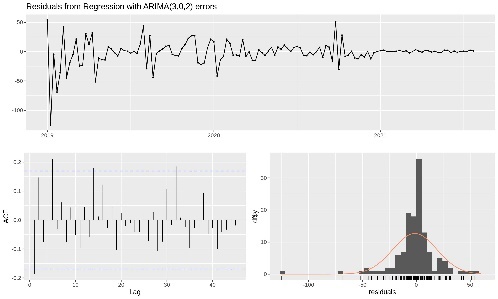 |  |
|  | ITS - C | 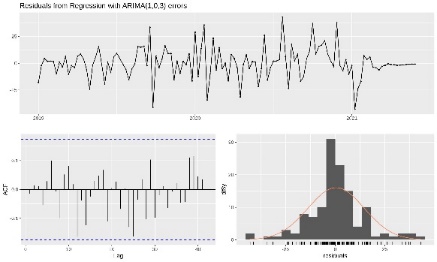 | 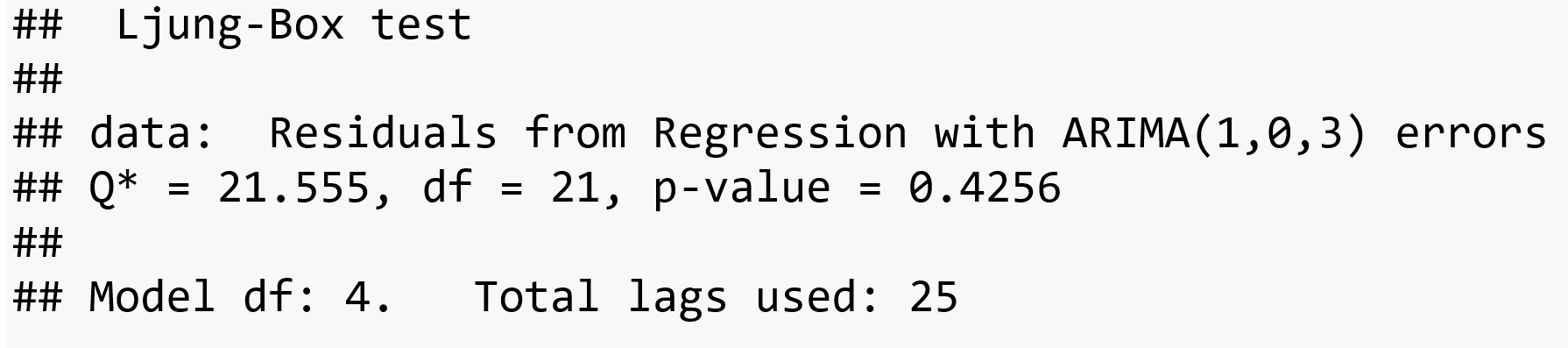 | 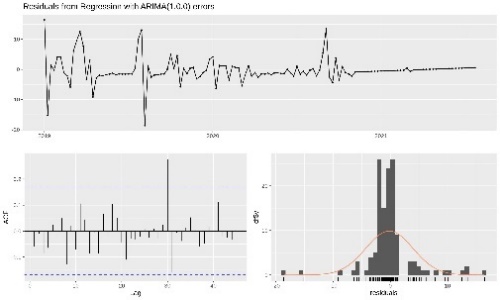 |  |
|  | CITS - I | 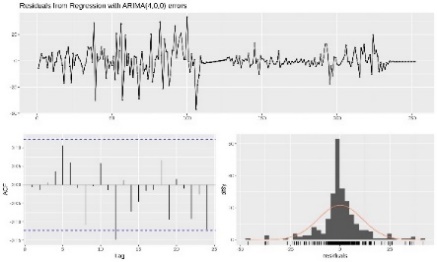 | 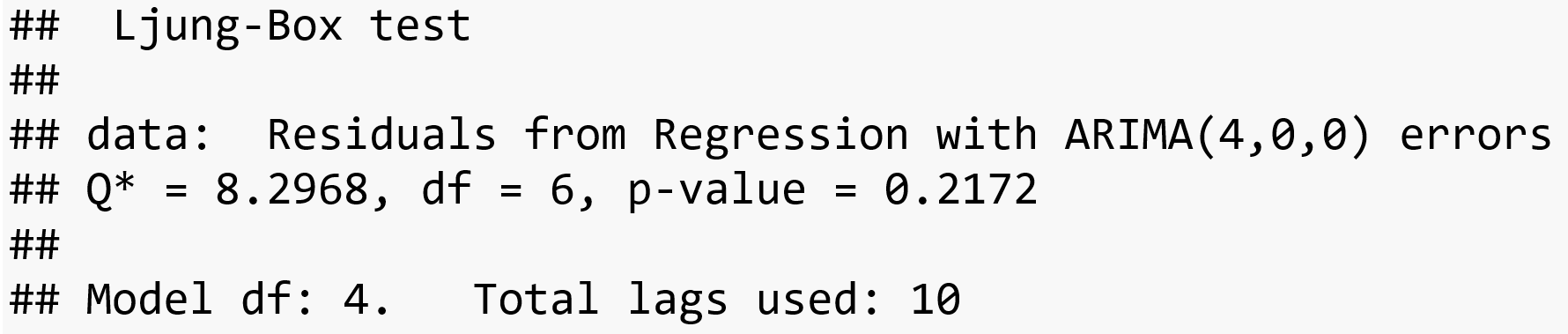 | 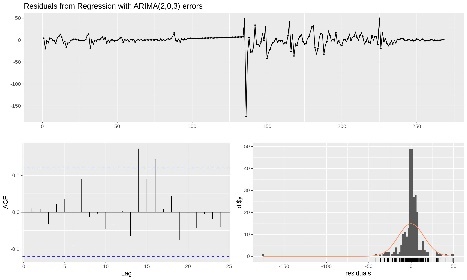 | 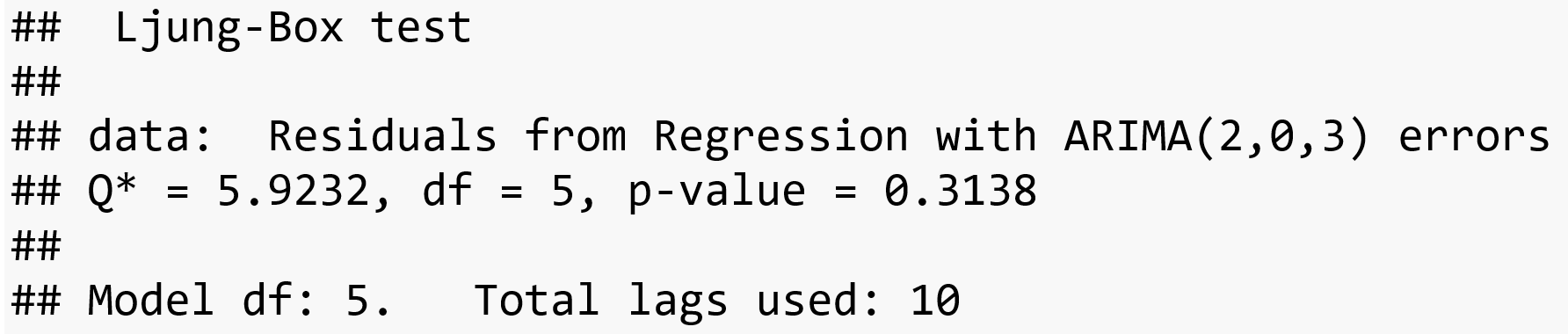 |
| 3G cephalosporin | ITS - I | 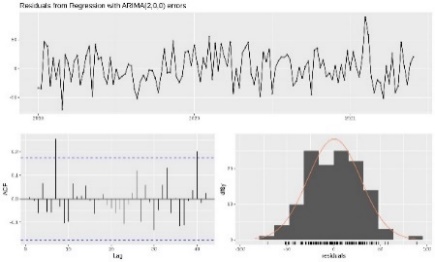 | 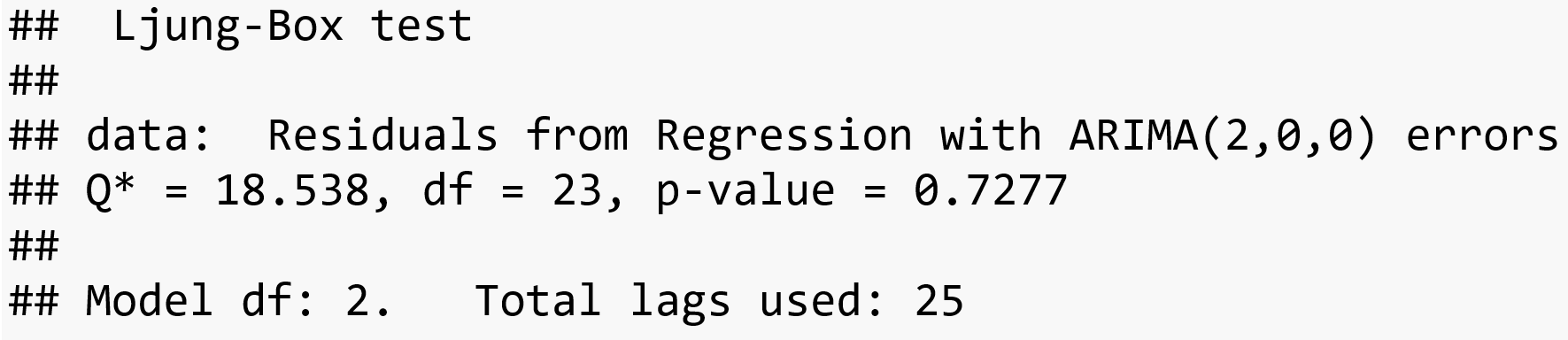 | 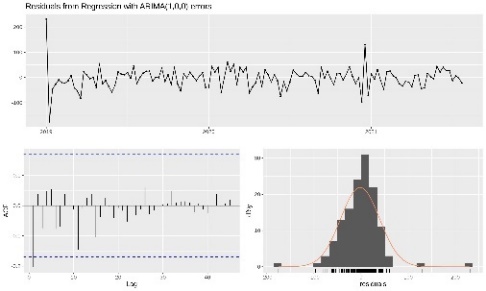 |  |
|  | ITS - C | 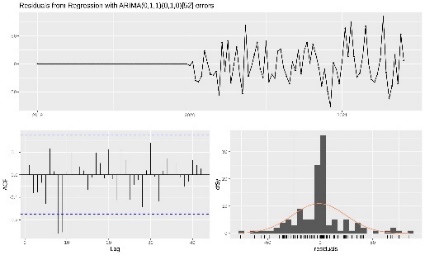 | 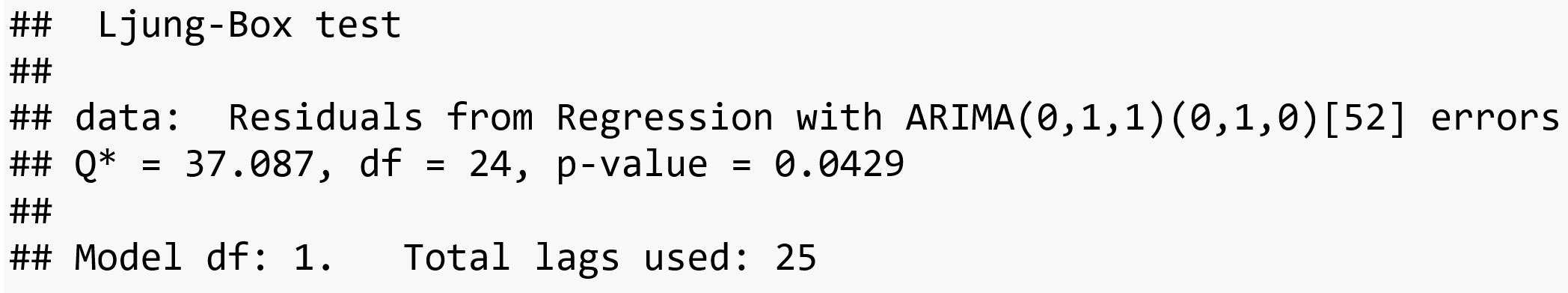 | 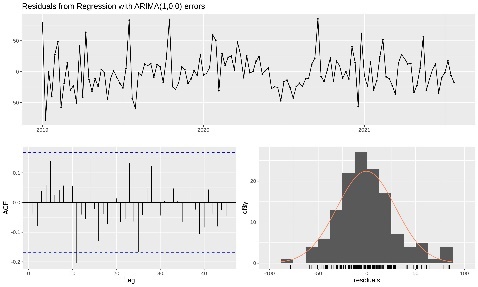 |  |
|  | CITS - I | 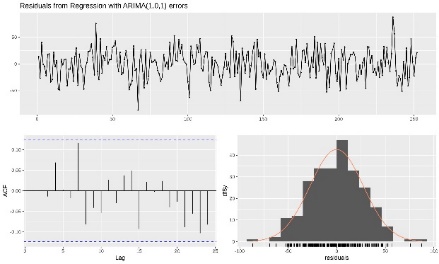 | 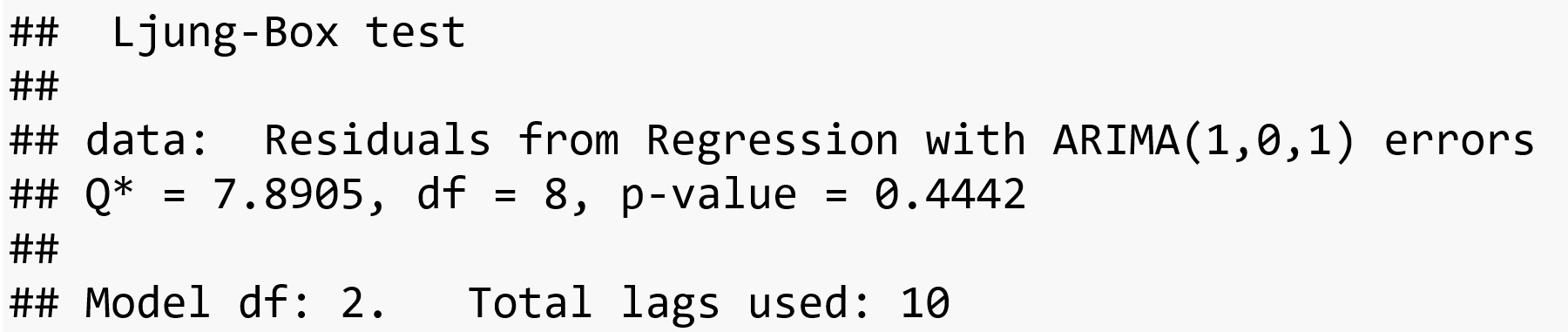 | 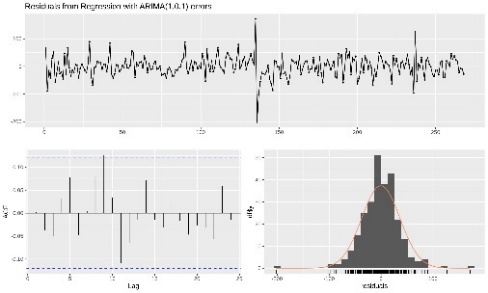 | 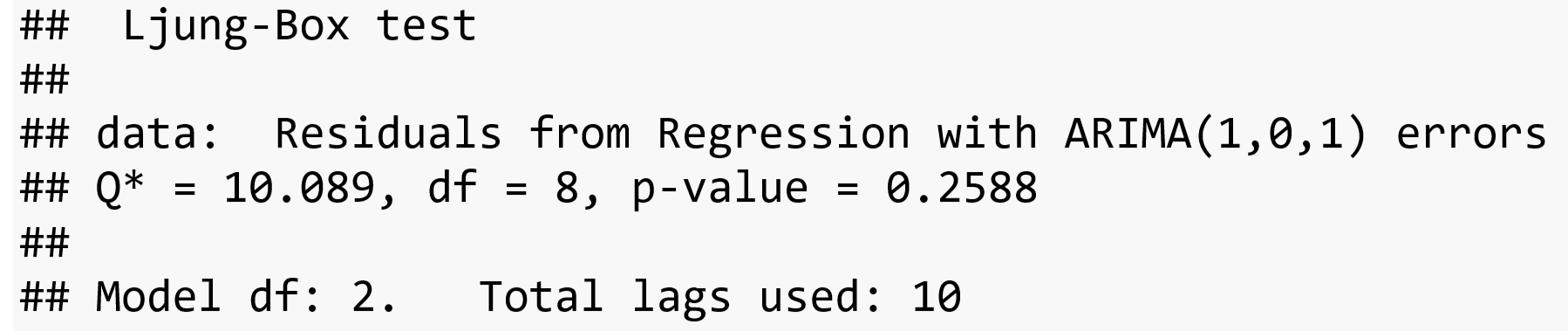 |
| 2G cephalosporin | ITS - I | 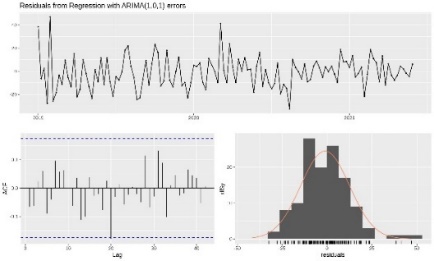 | 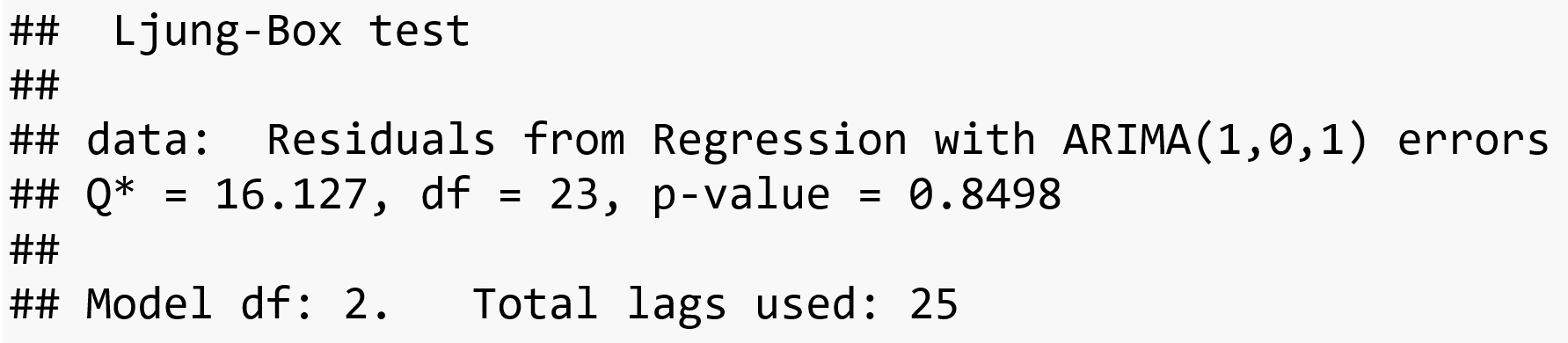 | 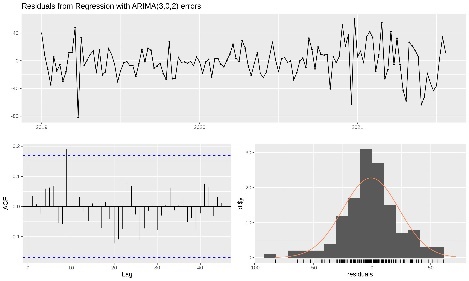 |  |
|  | ITS - C | 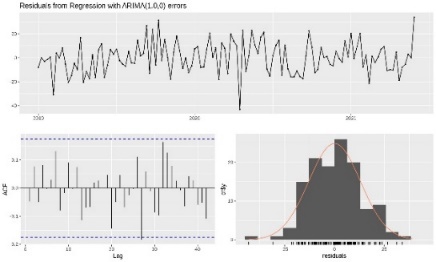 | 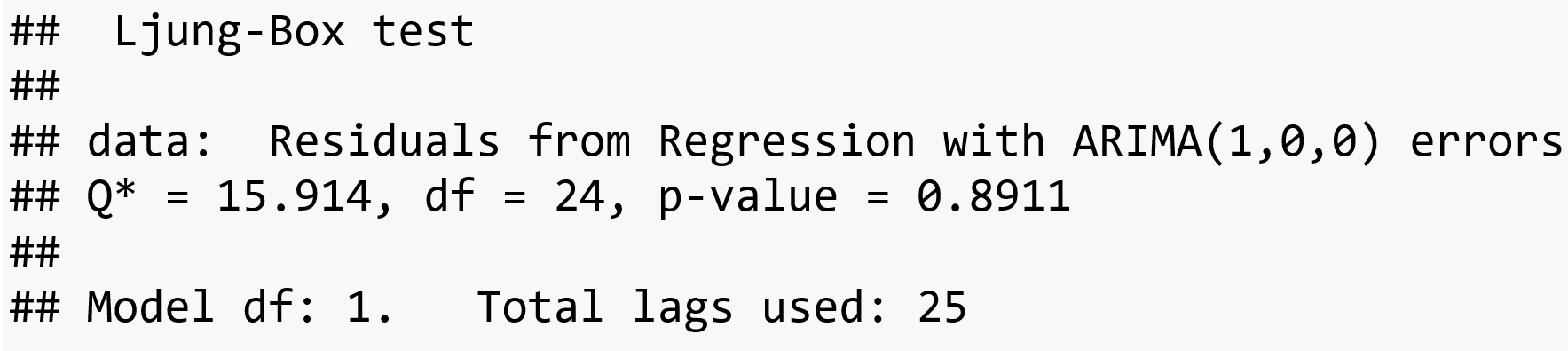 | 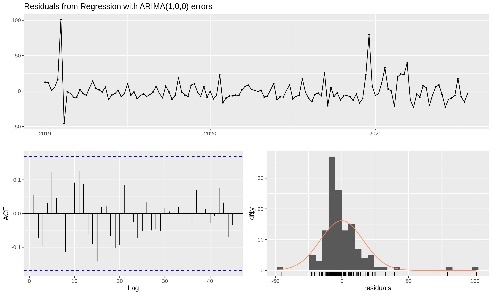 |  |
|  | CITS - I | 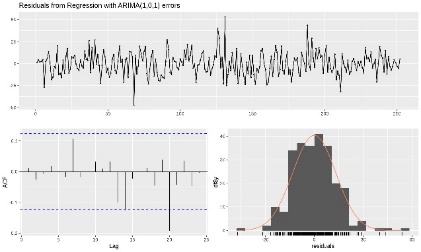 | 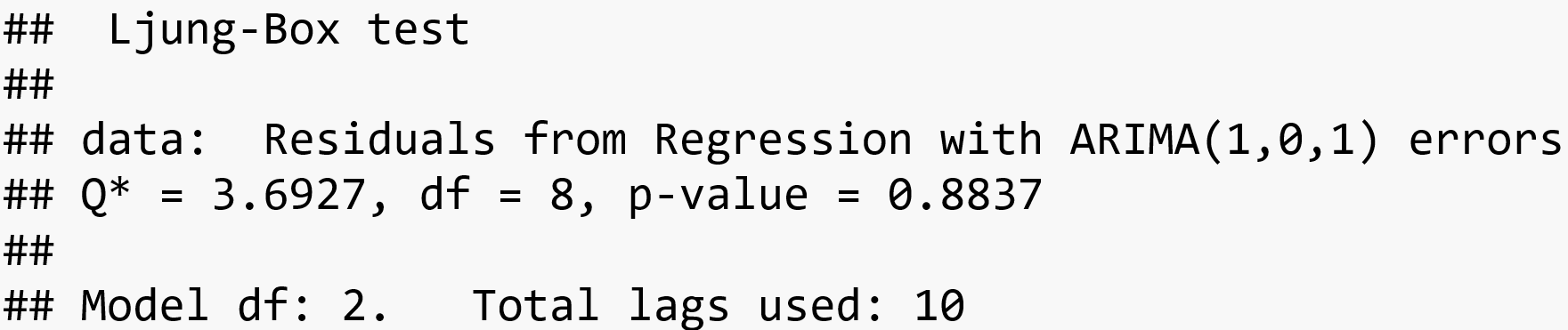 | 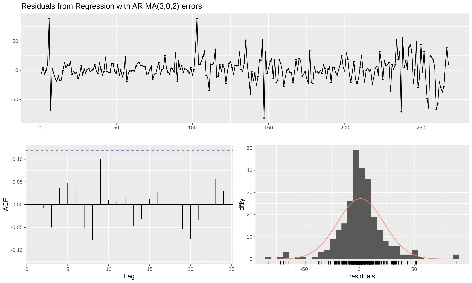 | 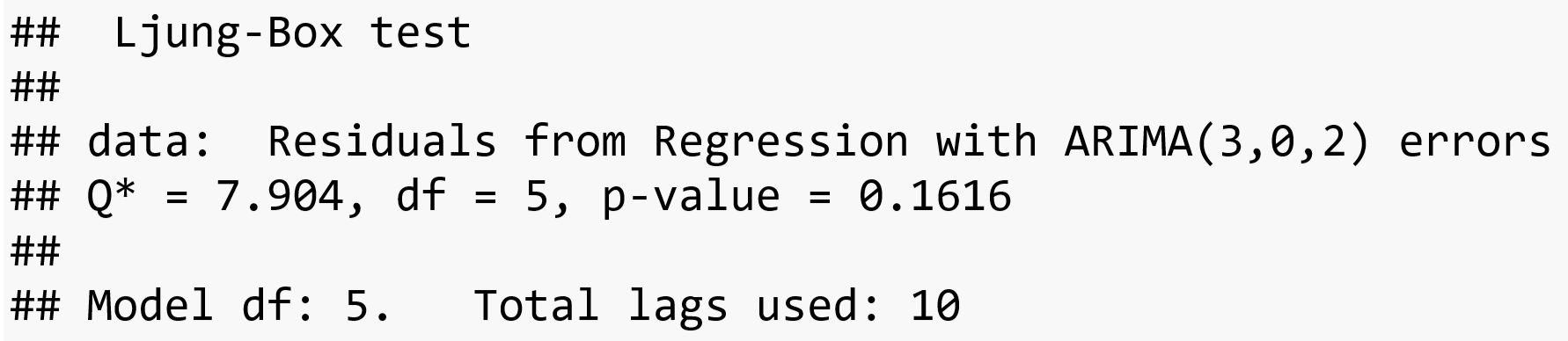 |
| Fluoroquinolones | ITS - I | 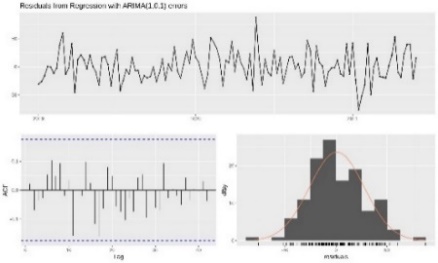 | 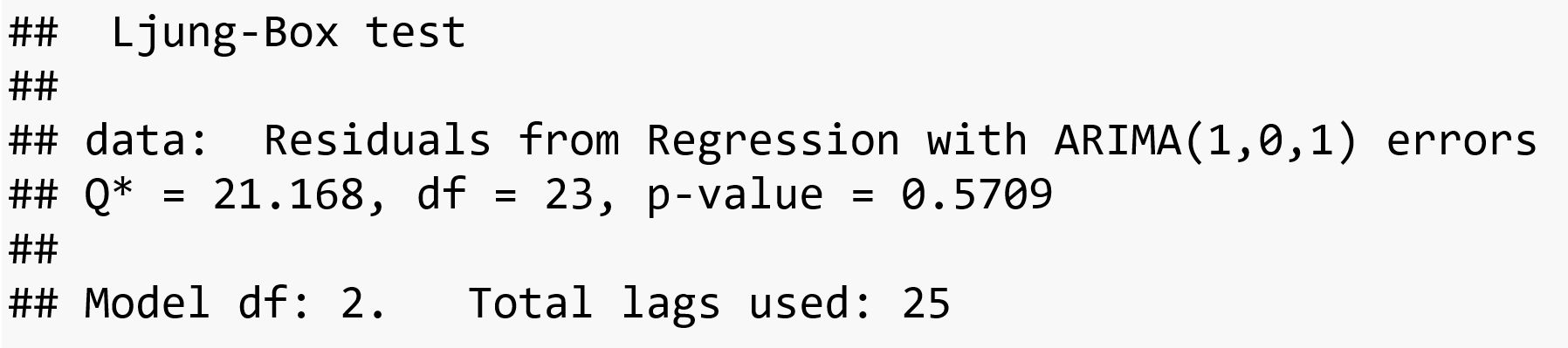 | 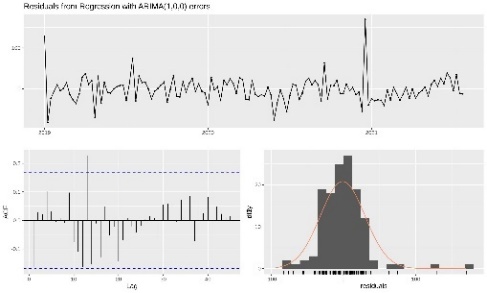 |  |
|  | ITS - C | 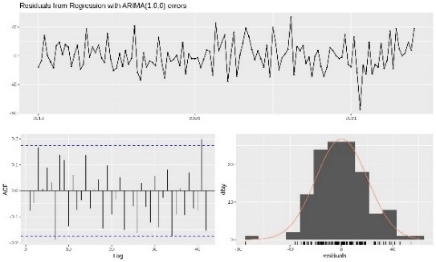 | 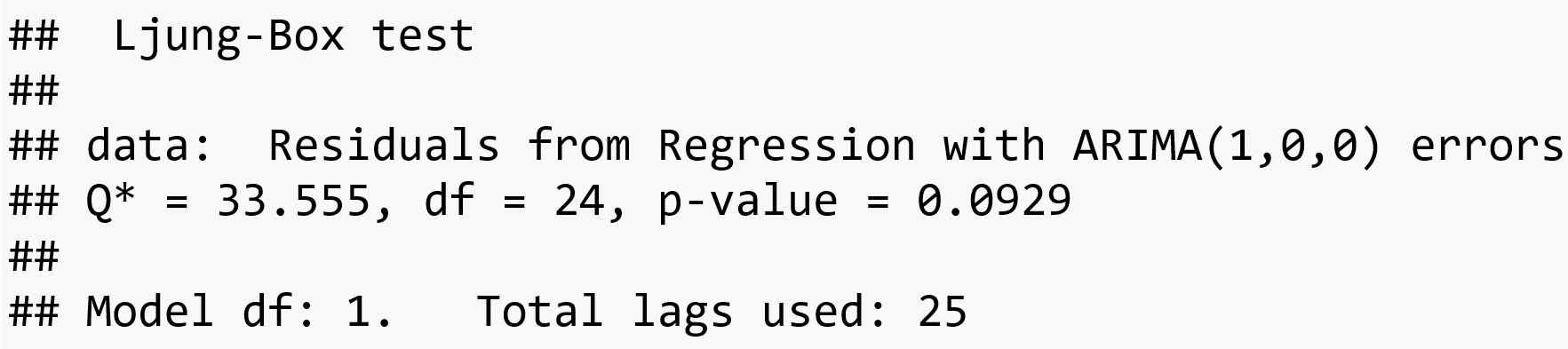 | 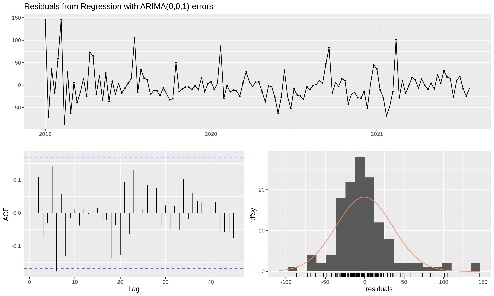 |  |
|  | CITS - I | 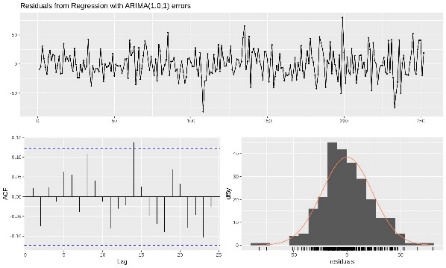 | 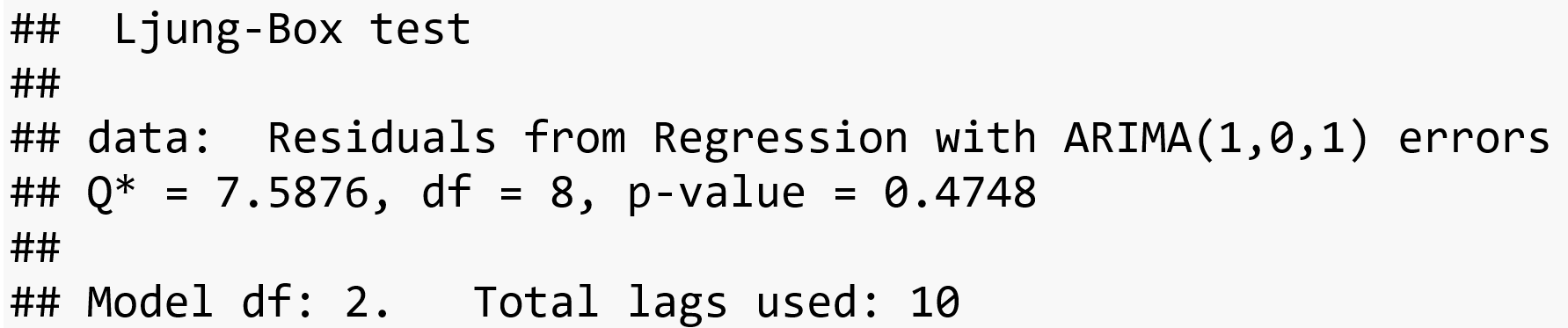 | 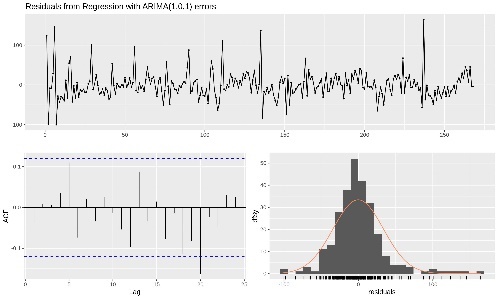 | 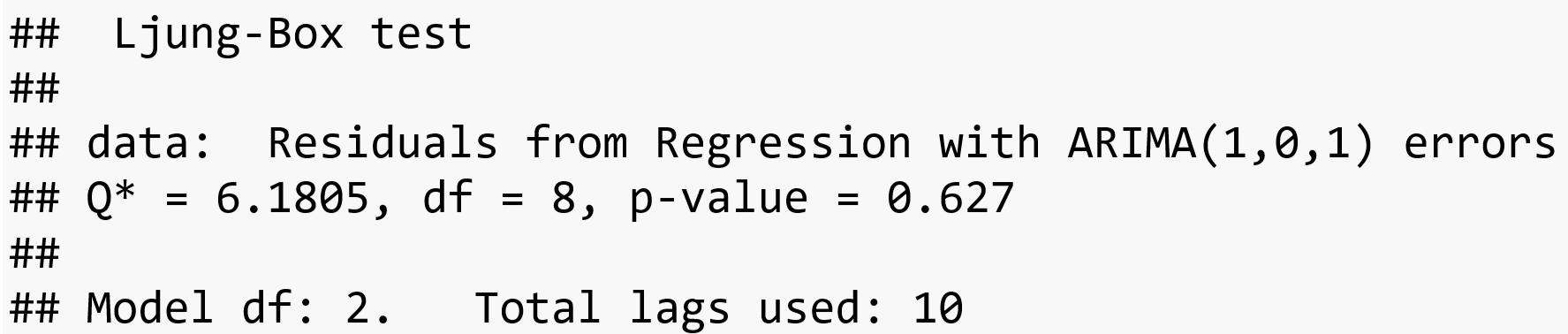 |
| Penicillin/ beta-lactamase inhibitors | ITS - I | 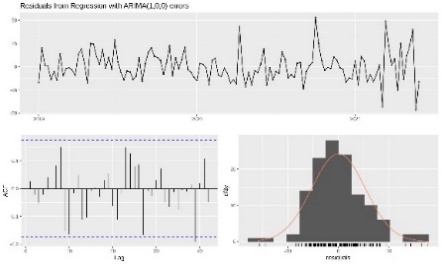 | 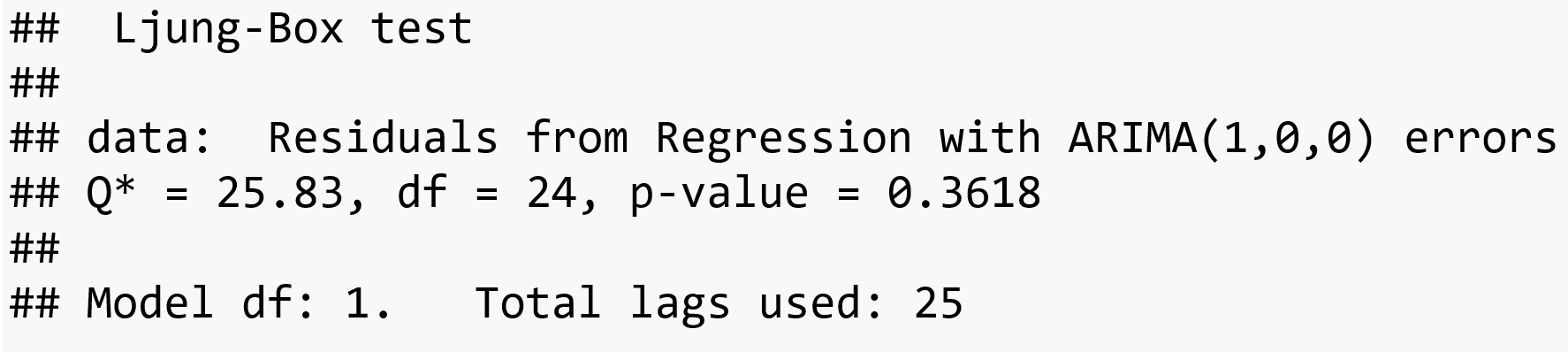 | 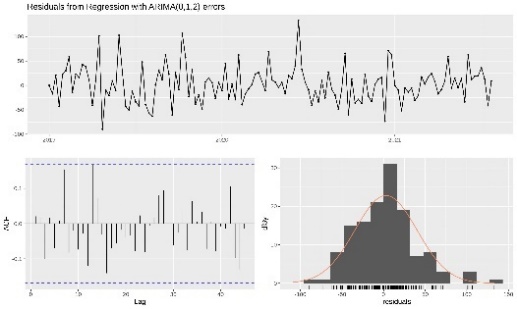 |  |
|  | ITS - C | 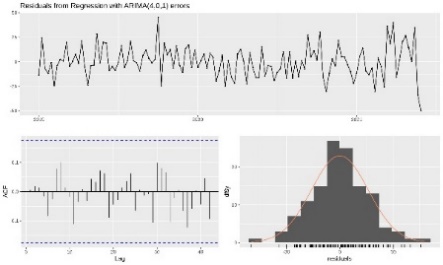 | 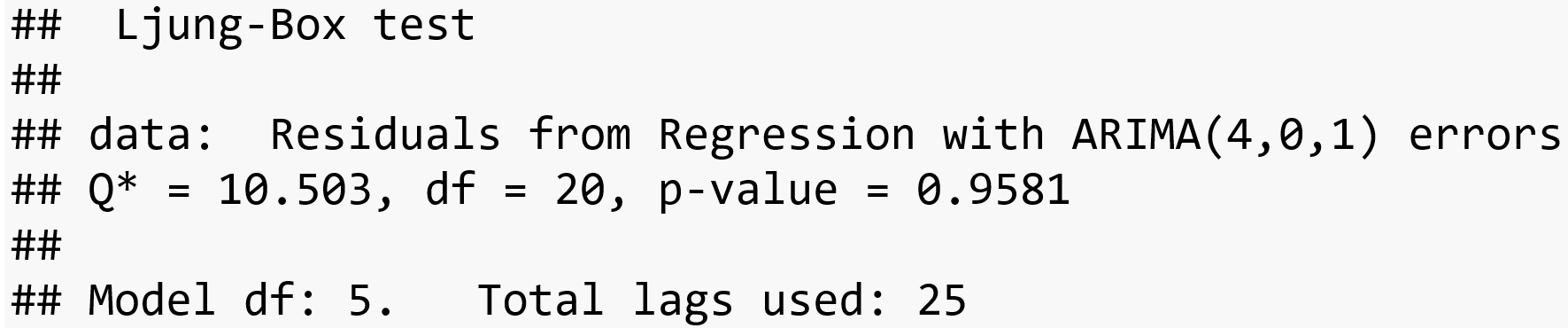 | 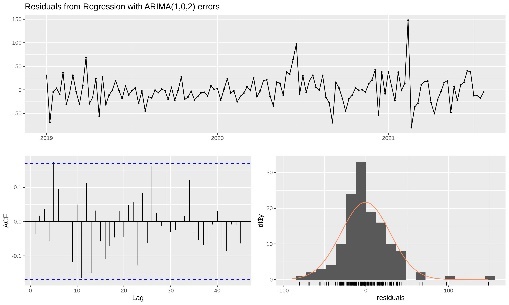 |  |
|  | CITS - I | 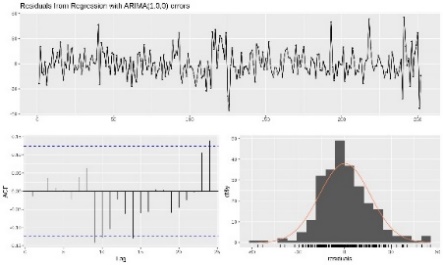 | 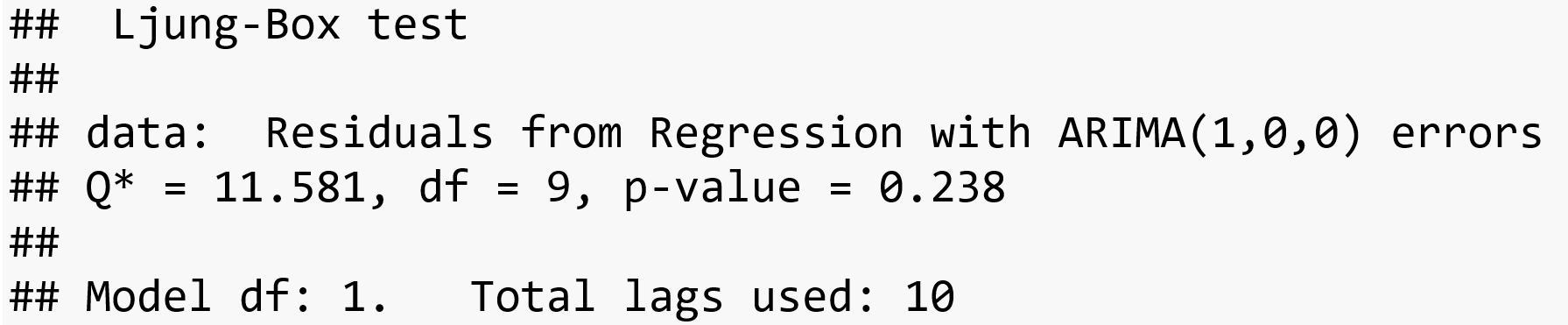 | 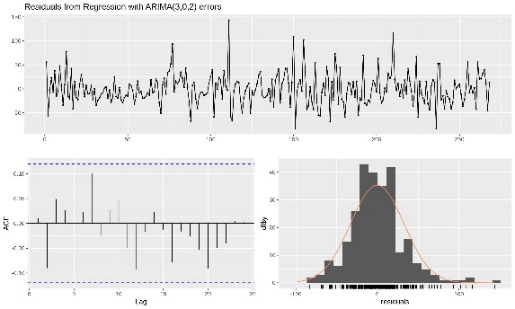 | 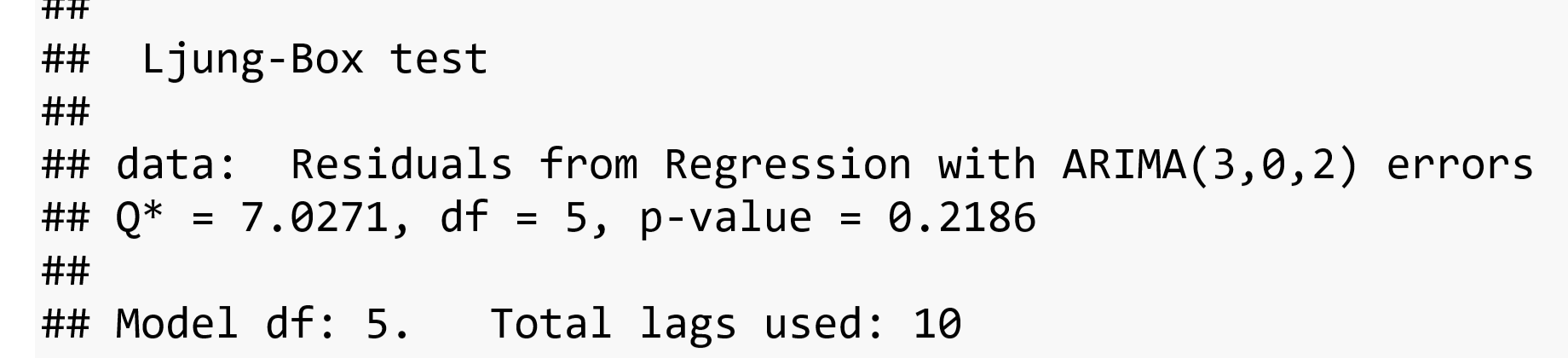 |
| Beta-lactamase resistant penicillin | ITS - I | 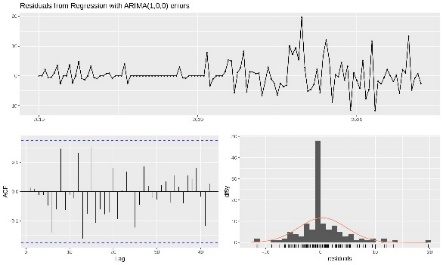 | 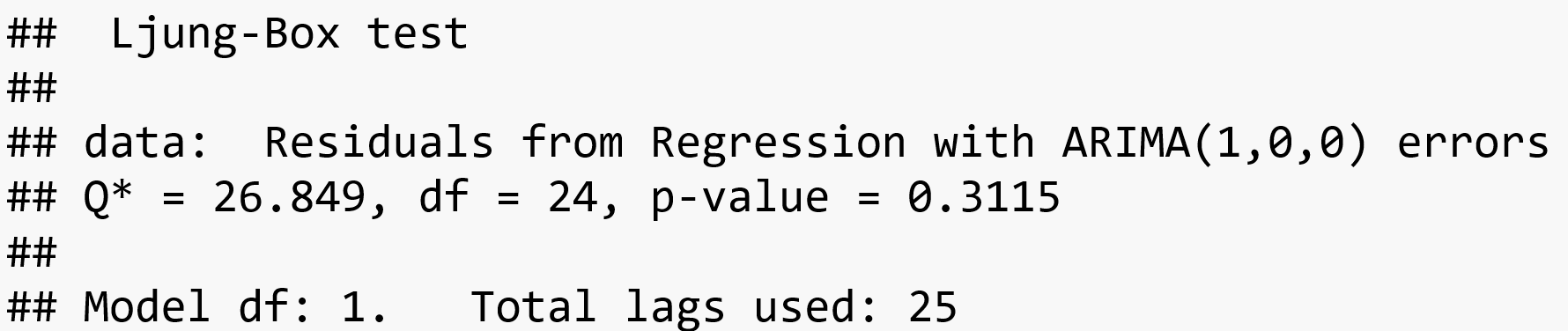 | 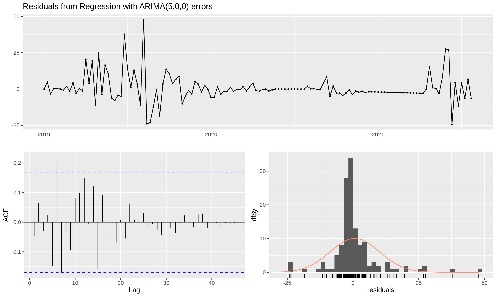 |  |
|  | ITS - C | 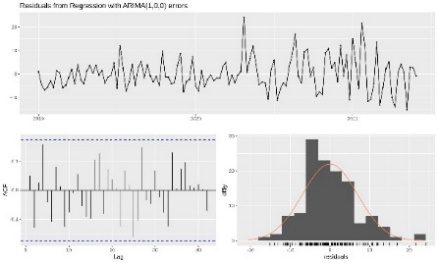 |  |  |  |
|  | CITS - I |  |  |  |  |
| Aminoglycosides | ITS - I |  |  |  |  |
|  | ITS - C |  |  |  |  |
|  | CITS - I |  |  |  |  |
| Glycopeptide antibacterials | ITS - I |  |  |  |  |
|  | ITS - C |  |  |  |  |
|  | CITS - I |  |  |  |  |
| Polymyxins | ITS - I |  |  |  |  |
|  | ITS - C |  |  |  |  |
|  | CITS - I |  |  |  |  |
| Imidazole derivatives | ITS - I |  |  |  |  |
|  | ITS - C |  |  |  |  |
|  | CITS - I |  |  |  |  |
| Macrolides | ITS - I |  |  |  |  |
|  | ITS - C |  |  |  |  |
|  | CITS - I |  |  |  |  |
| ***By study ward pairs*** |  |  |  |  |  |
| ICU pair | ITS - I |  |  |  |  |
|  | ITS - C |  |  |  |  |
|  | CITS - I |  |  |  |  |
| Surgical pair | ITS - I |  |  |  |  |
|  | ITS - C |  |  |  |  |
|  | CITS - I |  |  |  |  |
| Internal pair 1 | ITS - I |  |  |  |  |
|  | ITS - C |  |  |  |  |
|  | CITS - I |  |  |  |  |
| Internal pair 2 | ITS - I |  |  |  |  |
|  | ITS - C |  |  |  |  |
|  | CITS - I |  |  |  |  |

## Table S21. Diagnostic plots for ITS/CITS ARIMA models for antibiotic non-susceptibility outcomes

Note: ITS – I models are ITS models for the intervention time-series only, ITS – C models are ITS models for the control time-series only, CITS-I model estimates intervention group adjusting for control. Segmented regression model assumptions were checked by examining the residuals, particularly temporal correlation using ACF/PACF plots and tests for residuals. Below are the results of checking the residuals. Models are considered to be valid when the residuals are approximately normally distributed and the residuals test returns a non-significant p value. All residual and test for autocorrelation of residuals were generated using checkresiduals() from the package forecast in R.

| **Models and data series** | | **Hospital 1** | | **Hospital 2** | |
| --- | --- | --- | --- | --- | --- |
|  |  | **Residuals from regression with ARIMA errors** | **Tests for residuals** | **Residuals from regression with ARIMA errors** | **Tests for residuals** |
| ***E.coli*** | | | | | |
| Aminoglycoside | ITS - I |  |  |  |  |
|  | ITS - C |  |  |  |  |
|  | CITS - I |  |  |  |  |
| Carbapenem | ITS - I |  |  |  |  |
|  | ITS - C |  |  |  |  |
|  | CITS - I |  |  |  |  |
| Ciprofloxacin | ITS - I |  |  |  |  |
|  | ITS - C |  |  |  |  |
|  | CITS - I |  |  |  |  |
| Third-generation cephalosporin | ITS - I |  |  |  |  |
|  | ITS - C |  |  |  |  |
|  | CITS - I |  |  |  |  |
| ***Klebsiella* spp.** | | | | | |
| Aminoglycoside | ITS - I |  |  |  |  |
|  | ITS - C |  |  |  |  |
|  | CITS - I |  |  |  |  |
| Carbapenem | ITS - I |  |  |  |  |
|  | ITS - C |  |  |  |  |
|  | CITS - I |  |  |  |  |
| Ciprofloxacin | ITS - I |  |  |  |  |
|  | ITS - C |  |  |  |  |
|  | CITS – I |  |  |  |  |
| Third-generation cephalosporin | ITS - I |  |  |  |  |
|  | ITS - C |  |  |  |  |
|  | CITS - I |  |  |  |  |
| ***Pseudomonas aeruginosa*** | | | | | |
| Aminoglycoside | ITS - I |  |  |  |  |
|  | ITS - C |  |  |  |  |
|  | CITS - I |  |  |  |  |
| Carbapenem | ITS - I |  |  |  |  |
|  | ITS - C |  |  |  |  |
|  | CITS - I |  |  |  |  |
| Ciprofloxacin | ITS - I |  |  |  |  |
|  | ITS - C |  |  |  |  |
|  | CITS - I |  |  |  |  |
| Ceftazidime | ITS - I |  |  |  |  |
|  | ITS - C |  |  |  |  |
|  | CITS - I |  |  |  |  |
| Piperacillin-tazobactam | ITS - I |  |  |  |  |
|  | ITS - C |  |  |  |  |
|  | CITS - I |  |  |  |  |
| ***Acinetobacter* spp.** | | | | | |
| Aminoglycoside | ITS - I |  |  |  |  |
|  | ITS - C |  |  |  |  |
|  | CITS - I |  |  |  |  |
| Carbapenem | ITS - I |  |  |  |  |
|  | ITS - C |  |  |  |  |
|  | CITS - I |  |  |  |  |
| Ciprofloxacin | ITS - I |  |  |  |  |
|  | ITS - C |  |  |  |  |
|  | CITS - I |  |  |  |  |
| Ceftazidime | ITS - I |  |  |  |  |
|  | ITS - C |  |  |  |  |
|  | CITS - I |  |  |  |  |
| Piperacillin-tazobactam | ITS - I |  |  |  |  |
|  | ITS - C |  |  |  |  |
|  | CITS - I |  |  |  |  |
| ***Staphylococcus aureus*** | | | | | |
| MRSA | ITS - I |  |  |  |  |
|  | ITS - C |  |  |  |  |
|  | CITS - I |  |  |  |  |

## Table S22. Diagnostic plots for ITS/CITS ARIMA models for in-hospital mortality outcomes

Note: ITS – I models are ITS models for the intervention time-series only, ITS – C models are ITS models for the control time-series only, CITS-I model estimates intervention group adjusting for control. Segmented regression model assumptions were checked by examining the residuals, particularly temporal correlation using ACF/PACF plots and tests for residuals. Below are the results of checking the residuals. Models are considered to be valid when the residuals are approximately normally distributed and the residuals test returns a non-significant p value. All residual and test for autocorrelation of residuals were generated using checkresiduals() from the package forecast in R.

| **Models and data series** | | **Hospital 1** | | **Hospital 2** | |
| --- | --- | --- | --- | --- | --- |
|  |  | **Residuals from regression with ARIMA errors** | **Tests for residuals** | **Residuals from regression with ARIMA errors** | **Tests for residuals** |
| All study wards | ITS - I |  |  |  |  |
|  | ITS - C |  |  |  |  |
|  | CITS - I |  |  |  |  |
| ICU pair | ITS – I |  |  |  |  |
|  | ITS – C |  |  |  |  |
|  | CITS - I |  |  |  |  |
| Surgical pair | ITS – I |  |  |  |  |
|  | ITS – C |  |  |  |  |
|  | CITS - I |  |  |  |  |
| Internal pair 1 | ITS – I |  |  |  |  |
|  | ITS – C |  |  |  |  |
|  | CITS - I |  |  |  |  |
| Internal pair 2 | ITS – I |  |  |  |  |
|  | ITS – C |  |  |  |  |
|  | CITS - I |  |  |  |  |

## Table S23. Diagnostic plots for ITS/CITS ARIMA models for costs of hospitalization

Note: ITS – I models are ITS models for the intervention time-series only, ITS – C models are ITS models for the control time-series only, CITS-I model estimates intervention group adjusting for control. Segmented regression model assumptions were checked by examining the residuals, particularly temporal correlation using ACF/PACF plots and tests for residuals. Below are the results of checking the residuals. Models are considered to be valid when the residuals are approximately normally distributed and the residuals test returns a non-significant p value. All residual and test for autocorrelation of residuals were generated using checkresiduals() from the package forecast in R.

| **Models and data series** | | **Hospital 1** | | **Hospital 2** | |
| --- | --- | --- | --- | --- | --- |
|  |  | **Residuals from regression with ARIMA errors** | **Tests for residuals** | **Residuals from regression with ARIMA errors** | **Tests for residuals** |
| All study wards | ITS - I |  |  |  |  |
|  | ITS - C |  |  |  |  |
|  | CITS - I |  |  |  |  |
| ICU pair | ITS – I |  |  |  |  |
|  | ITS – C |  |  |  |  |
|  | CITS - I |  |  |  |  |
| Surgical pair | ITS – I |  |  |  |  |
|  | ITS – C |  |  |  |  |
|  | CITS - I |  |  |  |  |
| Internal pair 1 | ITS – I |  |  |  |  |
|  | ITS – C |  |  |  |  |
|  | CITS - I |  |  |  |  |
| Internal pair 2 | ITS – I |  |  |  |  |
|  | ITS – C |  |  |  |  |
|  | CITS - I |  |  |  |  |

# **References**

1. Huong VTL, Ngan TTD, Thao HP, et al. Assessing feasibility of establishing antimicrobial stewardship programmes in two provincial-level hospitals in Vietnam: an implementation research study. BMJ Open 2021;11(10):e053343. doi: 10.1136/bmjopen-2021-053343 [↑](#endnote-ref-1)
2. Minh LHN KQN, Le TN, Khanh PNQ and Huy NT. COVID-19 Timeline of Vietnam: Important Milestones Through Four Waves of the Pandemic and Lesson Learned. Front Public Health 2021; 9(709067). doi: 10.3389/fpubh.2021.709067 [↑](#endnote-ref-2)
3. Schaffer AL, Dobbins TA, Pearson SA. Interrupted time series analysis using autoregressive integrated moving average (ARIMA) models: a guide for evaluating large-scale health interventions. BMC Med Res Methodol 2021;21(1):58. doi: 10.1186/s12874-021-01235-8 [↑](#endnote-ref-3)
4. Lüdecke D, Ben-Shachar MS, Patil I, et al. performance: An R Package for Assessment, Comparison and Testing of Statistical Models. Journal of Open Source Software 2021;6(60):3139. doi: 10.21105/joss.03139 [↑](#endnote-ref-4)
5. Tay JK, Narasimhan B, Hastie T. Elastic Net Regularization Paths for All Generalized Linear Models. Journal of Statistical Software 2023; 106(1), 1-31. doi:10.18637/jss.v106.i01 [↑](#endnote-ref-5)
6. Magiorakos AP, Srinivasan A, Carey RB, et al. Multidrug-resistant, extensively drug-resistant and pandrug-resistant bacteria: an international expert proposal for interim standard definitions for acquired resistance. Clin Microbiol Infect 2012;18(3):268-81. doi: 10.1111/j.1469-0691.2011.03570.x [↑](#endnote-ref-6)
